# Host cell interactions of outer membrane vesicle-associated virulence factors of enterohemorrhagic *Escherichia coli* O157: Intracellular delivery, trafficking and mechanisms of cell injury

**DOI:** 10.1371/journal.ppat.1006159

**Published:** 2017-02-03

**Authors:** Martina Bielaszewska, Christian Rüter, Andreas Bauwens, Lilo Greune, Kevin-André Jarosch, Daniel Steil, Wenlan Zhang, Xiaohua He, Roland Lloubes, Angelika Fruth, Kwang Sik Kim, M. Alexander Schmidt, Ulrich Dobrindt, Alexander Mellmann, Helge Karch

**Affiliations:** 1 Institute of Hygiene, University of Münster, Münster, Germany; 2 Institute of Infectiology, Center for Molecular Biology of Inflammation (ZMBE), University of Münster, Münster, Germany; 3 Western Regional Research Center, Agricultural Research Service, United States Department of Agriculture (USDA), Albany, California, United States of America; 4 Laboratoire d'Ingenierie des Systemes Macromoleculaires UMR7255, CNRS-Aix-Marseille Université, Marseille, France; 5 National Reference Center for Salmonella and Other Enteric Pathogens, Robert Koch Institute, Branch Wernigerode, Wernigerode, Germany; 6 Division of Pediatric Infectious Diseases, Johns Hopkins University School of Medicine, Baltimore, Maryland, United States of America; 7 Interdisciplinary Center for Clinical Research (IZKF), University of Münster, Münster, Germany; INSERM U1220, FRANCE

## Abstract

Outer membrane vesicles (OMVs) are important tools in bacterial virulence but their role in the pathogenesis of infections caused by enterohemorrhagic *Escherichia coli* (EHEC) O157, the leading cause of life-threatening hemolytic uremic syndrome, is poorly understood. Using proteomics, electron and confocal laser scanning microscopy, immunoblotting, and bioassays, we investigated OMVs secreted by EHEC O157 clinical isolates for virulence factors cargoes, interactions with pathogenetically relevant human cells, and mechanisms of cell injury. We demonstrate that O157 OMVs carry a cocktail of key virulence factors of EHEC O157 including Shiga toxin 2a (Stx2a), cytolethal distending toxin V (CdtV), EHEC hemolysin, and flagellin. The toxins are internalized by cells via dynamin-dependent endocytosis of OMVs and differentially separate from vesicles during intracellular trafficking. Stx2a and CdtV-B, the DNase-like CdtV subunit, separate from OMVs in early endosomes. Stx2a is trafficked, in association with its receptor globotriaosylceramide within detergent-resistant membranes, to the Golgi complex and the endoplasmic reticulum from where the catalytic Stx2a A1 fragment is translocated to the cytosol. CdtV-B is, after its retrograde transport to the endoplasmic reticulum, translocated to the nucleus to reach DNA. CdtV-A and CdtV-C subunits remain OMV-associated and are sorted with OMVs to lysosomes. EHEC hemolysin separates from OMVs in lysosomes and targets mitochondria. The OMV-delivered CdtV-B causes cellular DNA damage, which activates DNA damage responses leading to G2 cell cycle arrest. The arrested cells ultimately die of apoptosis induced by Stx2a and CdtV via caspase-9 activation. By demonstrating that naturally secreted EHEC O157 OMVs carry and deliver into cells a cocktail of biologically active virulence factors, thereby causing cell death, and by performing first comprehensive analysis of intracellular trafficking of OMVs and OMV-delivered virulence factors, we provide new insights into the pathogenesis of EHEC O157 infections. Our data have implications for considering O157 OMVs as vaccine candidates.

## Author summary

Enterohemorrhagic *Escherichia coli* (EHEC) O157, the leading EHEC group causing diarrhea and the life-threatening hemolytic uremic syndrome in humans, produce several virulence factors which play distinct roles in the pathogenesis of these diseases. However, the mechanisms of their secretion and host cell injury are poorly understood. We show here that EHEC O157 strains isolated from patients shed nanostructures termed outer membrane vesicles (OMVs) which contain major EHEC O157 virulence factors including Shiga toxin 2a (Stx2a), cytolethal distending toxin V (CdtV), EHEC hemolysin, and flagellin. The OMVs are taken up by human intestinal epithelial and renal and brain microvascular endothelial cells, which are the major targets during EHEC O157 infections, and deliver the virulence factors intracellularly. Inside cells the virulence factors separate from OMVs and are transported via different pathways to their target compartments including the cytosol (Stx2a), nucleus (CdtV-B subunit), and mitochondria (EHEC hemolysin). Cells exposed to EHEC O157 OMVs develop G2 cell cycle arrest induced by CdtV-B-mediated DNA damage. This is followed by apoptotic cell death triggered by Stx2a and CdtV via caspase-9 activation. OMVs thus serve as novel tools of EHEC O157-mediated host injury and are quite likely involved in the pathogenesis of human diseases.

## Introduction

Enterohemorrhagic *Escherichia coli* (EHEC) O157, the leading EHEC serogroup causing human diseases including life-threatening hemolytic uremic syndrome (HUS) [1], consist of classical non-sorbitol-fermenting (NSF) O157:H7 and sorbitol-fermenting (SF) O157:H^-^ (non-motile) strains [2]. Several molecules contribute to the virulence of these pathogens. Shiga toxins (Stxs), ribosome-inactivating AB_5_ holotoxins composed of a monomeric enzymatically active A subunit and a pentameric receptor-binding B subunit [3, 4], are the major precipitants of the renal and brain microvascular endothelial injury that underlies HUS [1, 3–6]. Stx2a is the most common Stx type associated with HUS [7]. Other EHEC O157 toxins that may trigger the HUS-underlying pathology are the cytolethal distending toxin V (CdtV) [8] and EHEC hemolysin (EHEC-Hly) [9, 10]. CdtV, a heterotrimeric genotoxin and cyclomodulin consisting of CdtV-A, CdtV-B, and CdtV-C subunits [11, 12] is produced by most SF and a subset of NSF EHEC O157 strains [12, 13]. The toxin causes, via DNase-like activity of its B subunit, the DNA damage in human microvascular endothelial cells, which activates G2 checkpoint responses leading to G2 cell cycle arrest and ultimately cell death [8]. EHEC-Hly, a member of the repeats-in-toxin family [14] regularly expressed by NSF EHEC O157 strains [2], injures human microvascular endothelial cells by different mechanisms depending on its form. Free, soluble EHEC-Hly lyses these cells [9], whereas EHEC-Hly bound to bacterial membrane vesicles causes apoptosis [10]. Besides their endothelial cytotoxicity, Stxs and EHEC-Hly induce, alone or together with H7 flagellin and/or O157 lipopolysaccharide (LPS), secretion of proinflammatory cytokines [15–17], which play multiple roles in HUS development [1, 5]. Another virulence factor, the serine protease EspPα produced by NSF O157 strains [18], may contribute to the pathogenesis of HUS by interacting with the coagulation cascade by cleaving factor V [19] and with the complement system by degrading C3 and C5 [20].

Current understanding of pathogenetic mechanisms of EHEC O157 is largely based on studies using free, soluble toxins. The role of outer membrane vesicles (OMVs), bacteria-derived nanostructures [21] used by multiple pathogens for virulence factors secretion and host cell delivery [10, 22–27] in the pathogenesis of EHEC O157 infections is little understood. Although production of OMVs containing Stx has been reported in EHEC O157:H7 [28, 29], there is no information about the presence of non-Stx virulence cargoes in O157 OMVs and about interactions of the OMVs and OMV-associated virulence factors with pathogenetically relevant human cells. Here we characterized OMV production in EHEC O157:H7/H^-^ patients´ isolates and identified major EHEC O157 virulence factors carried by OMVs. We analyzed the OMVs for their abilities to deliver the virulence factors into human intestinal epithelial and renal and brain microvascular endothelial cells, which are the main targets during EHEC infections. We determined intracellular trafficking routes of OMVs and OMV-delivered toxins and toxin subunits, characterized their biological effects and mechanisms of cell injury. Our data identify OMVs as carriers for major bioactive EHEC O157 virulence factors and powerful tools for host cell injury, thereby providing new insights into the pathogenesis of EHEC O157 infections. Moreover, this is to our knowledge the first time that the intracellular trafficking of OMVs and of different virulence factors has been monitored in parallel.

## Results

### EHEC O157:H7/H^-^ strains produce OMVs which carry a cocktail of virulence factors

**Fig 1:**
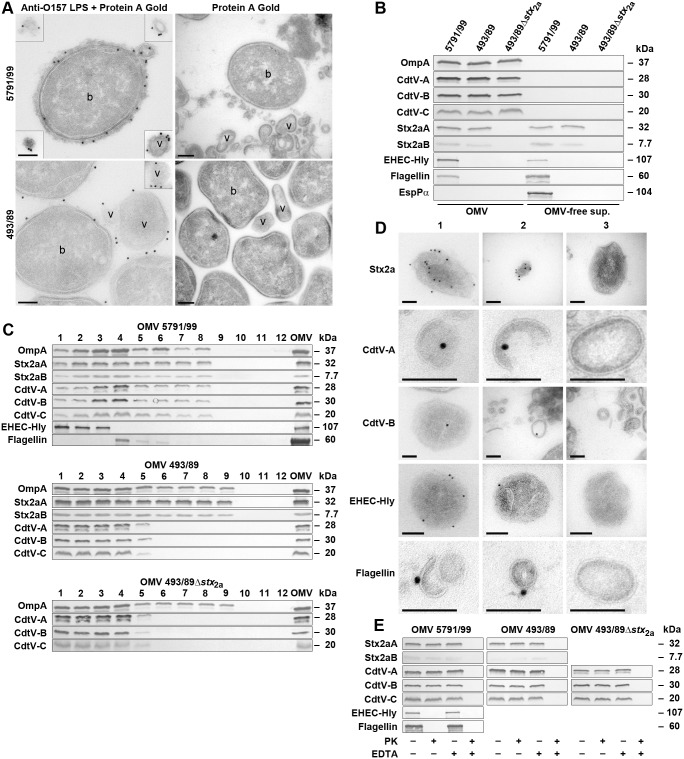
EHEC O157 OMVs carry a cocktail of virulence factors. (A) Electron microscopy of ultrathin cryosections of LB agar cultures of strains 5791/99 and 493/89 stained with anti-*E*. *coli* O157 LPS antibody and Protein A Gold or Protein A Gold alone (control). Examples of OMVs (v) and bacteria (b) are indicated. Frames depict OMVs located in other microscopic fields than the producing bacteria. Scale bars are 150 nm. (B) Distribution of virulence factors in OMVs and OMV-free supernatants determined by immunoblot with antibodies against OmpA (an OMV marker) and the indicated virulence proteins. (C) Distribution of virulence factors in OptiPrep density gradient fractions (1 to 12, collected from top to bottom) of O157 OMVs determined by immunoblot. The lanes designated OMV contain non-fractionated OMVs (positive control). (D) Localizations of virulence factors within 5791/99 OMVs visualized by electron microscopy of ultrathin cryosections of OptipPrep-purified OMVs stained with antibodies against the indicated virulence factors and Protein A Gold (panels 1 and 2) or Protein A Gold alone (panels 3; control). Bars are 100 nm. Note that by electron microscopy of immunostained ultrathin cryosections only 10%–15% of the total antigen present in the section can be detected [31] explaining relatively low numbers of signals observed for most virulence factors. (E) Immunoblots of proteinase K (PK)-untreated (PK-) and PK-treated (PK+) O157 OMVs either intact (EDTA-) or lysed with 0.1 M EDTA (EDTA+) with the indicated antibodies. (Anti-CdtV-C antibody is not suitable for electron microscopy but detects CdtV-C by immunoblot).

EHEC O157 patients´ isolates 5791/99 (NSF O157:H7) [13] and 493/89 (SF O157:H^-^) [30] (S1 Table) produced OMVs both on agar and in liquid media. OMV blebbing from the bacterial surface and OMVs liberated from the bacteria were demonstrated in LB agar cultures using electron microscopy (Fig 1A). Immunostaining of OMVs with anti-O157 LPS antibody (Fig 1A) confirmed that the OMV membrane was derived from the bacterial outer membrane. The kinetics of OMV production in LB broth correlated with bacterial growth and was similar in the EHEC strains and in a *stx*_2a_-negative derivative of strain 493/89 (493/89Δ*stx*_2a_) (S1A–S1C Fig).

Nano-LC-MS/MS analyses of OMV-associated proteins identified major virulence factors of the O157 strains (S1 Table) within OMVs. Specifically, Stx2a and CdtV holotoxins, EHEC-Hly, and H7 flagellin were found in 5791/99 OMVs, Stx2a and CdtV in 493/89 OMVs, and CdtV in 493/89Δ*stx*_2a_ OMVs (S2 and S3 Tables). In addition, > 50 other proteins originating from various bacterial compartments were identified in each OMV preparation (S2 and S3 Tables, S1D Fig).

To determine if the OMV-associated virulence factors also occur as OMV-free proteins, we performed immunoblot analyses of isolated OMVs and OMV-free supernatants. All three CdtV subunits (CdtV-A, -B, -C) were solely identified in OMVs of all strains (Fig 1B) demonstrating that OMVs represent a unique secretion pathway for this genotoxin in EHEC O157. Stx2a was almost equally distributed between OMVs and OMV-free supernatants of strains 5791/99 (56% and 44% of A subunit, and 51% and 49% of B subunit, respectively) and 493/89 (54% and 46% of A subunit, and 55% and 45% of B subunit, respectively) (Fig 1B). EHEC-Hly and H7 flagellin expressed by strain 5791/99 (S1 Table) were also OMV-associated (70% and 37%, respectively) and OMV-free (30% and 63%, respectively) (Fig 1B). A tight OMV-association of each respective virulence factor was confirmed by a dissociation assay, in which the membrane disruptant sodium dodecylsulfate (SDS) was the only chemical capable of releasing the virulence proteins from OMVs (S2 Fig). In contrast to the other virulence factors, the serine protease EspPα produced by strain 5791/99 (S1 Table) showed no OMV association and only occurred as an OMV-free protein (Fig 1B).

OptiPrep density gradient fractionation of O157 OMVs and analyses of the fractions for OmpA (an OMV marker) and the virulence factors by immunoblot demonstrated that the different fractions partially differ by virulence factors cargoes (Fig 1C). Specifically, 5791/99 OMVs in fractions 1 to 3 carry Stx2a, CdtV, and EHEC-Hly, whereas those in fractions 4 to 6 contain Stx2a, CdtV, and H7 flagellin, and those in fractions 7 and 8 Stx2a and CdtV. The 493/89 OMVs in fractions 1 to 5 contain Stx2a together with CdtV, whereas those in fractions 6 to 9 contain Stx2a only. All three CdtV components are also present in fractions 1 to 5 of OMVs 493/89Δ*stx*_2a_ (Fig 1C). Electron microscopy of 5791/99 and 493/89 OMV OptiPrep fractions using negative staining demonstrated that OMVs in all fractions were intact, and visualized OMVs of different sizes (S3 Fig). This was confirmed by dynamic light scattering (DLS) analysis of OMV size (S4A and S4B Fig). The average diameters (Z-averages) of OMVs in 5791/99 and 493/89 fractions determined by DLS ranged from 125.3 to 180.8 nm, and from 92.1 to 159.3 nm, respectively (S4C and S4D Fig). To investigate interactions of OMV-associated virulence factors cocktails with human intestinal epithelial and renal and brain microvascular endothelial cells, we used in next experiments the pool of OMV 5791/99 fractions 1 to 8 and pools of OMV 493/89 or 493/89Δ*stx*_2a_ fractions 1 to 9, respectively. The concentrations of the total protein, Stx2a, CdtV, EHEC-Hly, and flagellin in the OptiPrep-purified OMV pools are shown in S4 Table.

### Virulence factors localize to different sites within O157 OMVs

Electron microscopy of ultrathin cryosections of OptiPrep-purified 5791/99 OMVs using immunogold staining visualized Stx2a, CdtV-A, and CdtV-B mostly inside OMVs, whereas EHEC-Hly and H7 flagellin were located on the OMV surface (Fig 1D). Proteinase K treatment of intact 5791/99 OMVs degraded EHEC-Hly and flagellin, but not Stx2a A, Stx2a B, and CdtV-A, -B, and -C subunits (Fig 1E). This confirmed the intravesicular localization of Stx2a and CdtV holotoxins and the surface localization of EHEC-Hly and H7 flagellin. Localizations of Stx2a and CdtV in 493/89 and 493/89Δ*stx*_2a_ OMVs were identical to those in 5791/99 OMVs (Fig 1E).

### EHEC O157 OMVs are taken up by human intestinal epithelial cells and brain and renal microvascular endothelial cells via dynamin-dependent endocytosis

**Fig 2:**
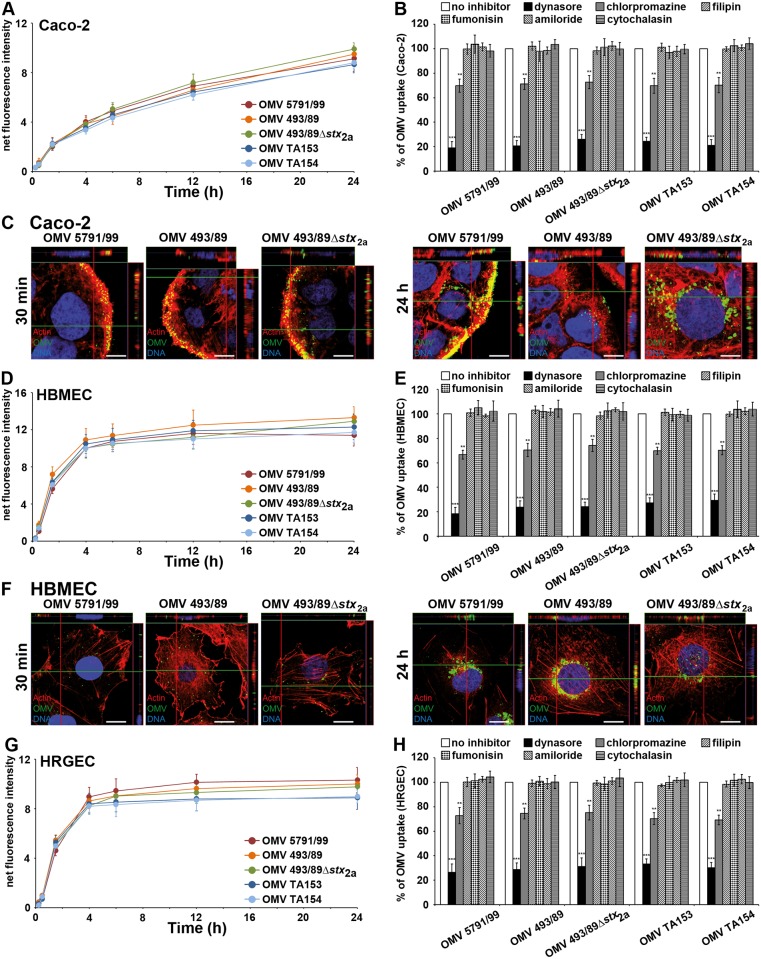
OMVs are taken up by target cells via dynamin-dependent endocytosis. (A, D, G) Kinetics of R-18 OMV uptake by indicated cell types. Fluorescence of cells incubated with OMVs was normalized to that of OMVs without cells (net fluorescence intensity). (C, F) CLSM visualization of OMV uptake by Caco-2 cells (C) and HBMEC (F) after 30 min and 24 h of incubation. Green, OMVs; red, actin; blue, nuclei. Confocal Z-stack projections are included at upper/right sides. Crosshairs show the position of the xy and yz planes. Scale bars are 10 μm. (B, E, H) Uptake of R-18 OMVs by Caco-2 cells (B), HBMEC (E), and HRGEC (H) which were pretreated with the indicated endocytosis inhibitors. OMV uptake in the presence of inhibitors was expressed as the percentage of OMV uptake by inhibitor-untreated cells (100%). Data in A, D, G, and B, E, H are means ± standard deviations from three independent experiments. ** *p* < 0.01, and *** *p* < 0.001 compared to inhibitor-untreated cells (one-way ANOVA).

Next, we asked if O157 OMVs interact with cells involved in the pathogenesis of EHEC-mediated diseases. Rhodamine isothiocyanate B-R18-labeled OMVs (hereafter termed R18-OMVs) were taken up by human intestinal epithelial cells (Caco-2), brain microvascular endothelial cells (HBMEC), and renal glomerular endothelial cells (HRGEC) in a time-dependent manner (Fig 2A, 2D and 2G). There were no significant differences between OMVs from NSF (5791/99) and SF (493/89) EHEC O157 strains, and OMVs containing (5791/99, 493/89) and lacking (493/89Δ*stx*_2a_) Stx2a (Fig 2A, 2D and 2G). The latter observation suggests a Stx2a-independent mechanism of OMV cellular uptake. To gain insight into the role of CdtV in this process, we used as controls CdtV-containing and CdtV-lacking R18-OMVs from recombinant strains TA153 (*E*. *coli* MC1061 harboring *cdt*V*-ABC* operon from strain 493/89 in SuperCos I) and TA154 (vector control), respectively (S1 Table, S5B Fig). OMVs from both strains were taken up by all cell types with similar kinetics and intensities, which were also similar to those of O157 OMVs (Fig 2A, 2D and 2G). This suggests that CdtV is dispensable for OMV cellular uptake. Moreover, the uptake of 5791/99 OMVs, which contain EHEC-Hly and H7 flagellin, was similar to those of 493/89 and 493/89Δ*stx*_2a_ OMVs, which lack these proteins (Figs 1B, 2A, 2D and 2G), suggesting that none of them is essential for OMV uptake.

Confocal laser scanning microscopy (CLSM) of Caco-2 cells and HBMEC exposed to O157, TA153 and TA154 OMVs for increasing durations confirmed that after initial cell binding at 4°C (S5A Fig, panels 0 min) OMVs were internalized when the temperature was raised to 37°C and accumulated perinuclearly in a time-dependent manner (Fig 2C and 2F and S5A and S5C Fig).

The uptake of R18-OMVs was significantly reduced by dynasore, an inhibitor of dynamin [32] (to ≤ 31% of their uptake by inhibitor-untreated cells; *p* < 0.001), and by chlorpromazine, an inhibitor of clathrin-mediated endocytosis [33] (to ≤ 75% of uptake by inhibitor-untreated cells; *p* < 0.01) (Fig 2B, 2E and 2H). Filipin III, a cholesterol-binding agent that disrupts lipid rafts and caveolae [34], and fumonisin B1, which inhibits sphingomyelin incorporation into the lipid rafts [35], had no effects on OMV uptake (Fig 2B, 2E and 2H). Similarly, no reduction of OMV uptake was caused by amiloride, an inhibitor of macropinocytosis [36], and cytochalasin D, an inhibitor of F-actin elongation [37] (Fig 2B, 2E and 2H). The activities of the inhibitors used were verified by their abilities to inhibit the uptake of established markers of the different endocytosis pathways (S6A and S6B Fig). The strong dynasore-mediated inhibition of OMV uptake observed in the fluorometric assay (Fig 2B, 2E and 2H) was confirmed by CLSM using Caco-2 cells and HBMEC. In contrast to dynasore-untreated cells, where multiple OMVs were detected intracellularly after 4 h of incubation (S5A and S5C Fig, panels 4 h), no or only sporadic OMVs were detected in dynasore-treated cells at this time point (S6C and S6E Fig). As a control, dynasore strongly inhibited the uptake of Alexa Fluor 488-conjugated transferrin and Alexa Fluor 488-conjugated cholera toxin B subunit (S6D and S6F Fig) that in both cases involves dynamin. Altogether, these experiments demonstrated that O157, as well as TA153 and TA154 OMVs, are internalized by human intestinal epithelial and brain and renal microvascular endothelial cells via dynamin-dependent and partially clathrin-mediated endocytosis.

### EHEC O157 virulence factors are internalized via OMVs and differentially separate from OMVs during intracellular trafficking

Immunoblot analyses of Caco-2 cells, HBMEC, and HRGEC, which had been treated with O157, TA153 or TA154 OMVs, demonstrated the presence of OMVs and the respective OMV-associated virulence proteins (Fig 1B) in cell lysates mostly after 30 min of exposure, with further increase of signals up to 4 h (S7 Fig). This is in agreement with increasing internalization of OMVs during time as demonstrated by CLSM (Fig 2C and 2F and S5A and S5C Fig) and indicates that OMVs deliver the virulence factors intracellularly.

**Fig 3:**
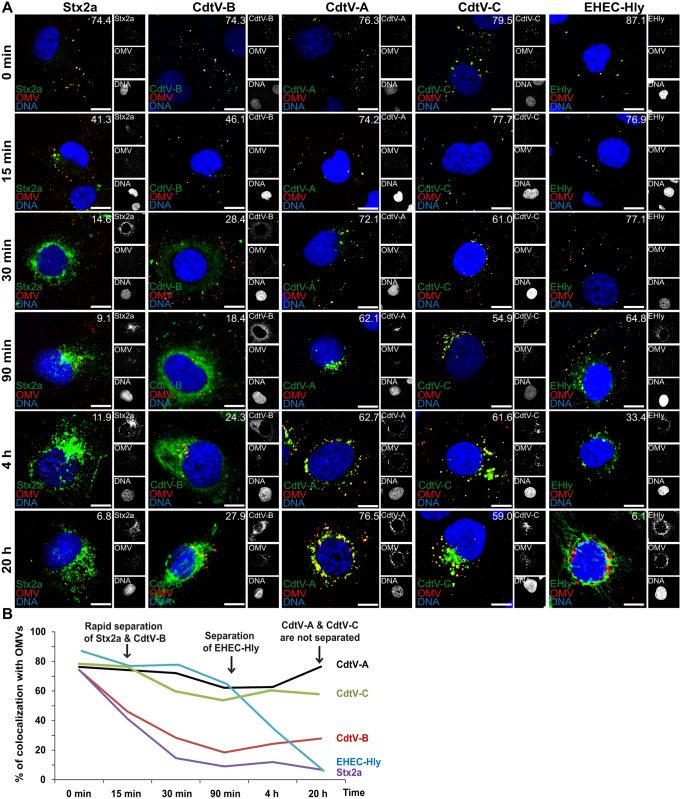
OMV-associated virulence proteins differentially separate from OMVs during intracellular trafficking. (A) CLSM of HBMEC exposed to 5791/99 OMVs for 30 min at 4°C (OMV binding; panels 0 min) followed by 15 min to 20 h at 37°C (OMV internalization). The indicated single fluorescence channels are shown in the right panels (enlargements are displayed in S9 and S10 Figs) and the merged images in the left panels (red, OMVs; green, virulence factors; blue, nuclei; yellow, colocalized red and green signals). The percentages of colocalizations between OMVs and virulence proteins were determined with the BioImageXD6 tool (white numbers). Scale bars are 10 μm. (B) Graphical summary of colocalizations of virulence factors with OMVs during time based on CLSM data shown in A. (Means of colocalizations from at least five different samples are shown in A and B; standard deviations and significance analysis see in S8A Fig).

To confirm the intracellular localization of OMV-delivered toxins and to monitor their association with OMVs during intracellular trafficking, HBMEC were incubated with 5791/99 OMVs, which contain all three major EHEC O157 toxins (Fig 1B) for 30 min at 4°C (OMV binding), followed by 15 min to 20 h at 37°C (OMV internalization), and analyzed by CLSM. Association of each toxin/toxin subunit with OMVs was quantified by calculating colocalization rates of the respective signals. Upon OMV cell binding, Stx2a, CdtV-A, -B, -C, and EHEC-Hly were all associated with OMVs as demonstrated by their high colocalization rates with vesicles (74.3%–87.1%) (Fig 3A, panels 0 min, and S8A Fig). After OMV internalization, Stx2a and CdtV-B rapidly separated from the vesicles as evidenced by a significant decrease of their colocalizations with OMVs until 30 min and further to 90 min of incubation at 37°C (Fig 3A and 3B and S8A Fig). After 4 h and 20 h, only minor subsets of Stx2a and CdtV-B remained OMV-associated (Fig 3A and 3B and S8A Fig). In contrast to CdtV-B, the CdtV-A and CdtV-C subunits strongly colocalized with OMVs throughout the 20 h experimental period indicating that the vast majorities of these proteins did not separate from OMVs (Fig 3A and 3B and S8A Fig). EHEC-Hly was associated with OMVs until 90 min of incubation and then promptly separated (Fig 3A and 3B and S8A Fig).

### Intracellular trafficking of O157 OMVs and OMV-delivered toxins

To determine the intracellular trafficking pathways of O157 OMVs and OMV-delivered toxins, we preincubated HBMEC with 5791/99 OMVs for 30 min at 4°C, postincubated for 30 min to 20 h at 37°C, and quantified colocalizations of OMVs and each toxin/toxin subunit with marker proteins of subcellular compartments and with nuclear DNA by CLSM. To confirm and extend the CLSM data, we isolated subcellular fractions from HBMEC which had been postincubated with 5791/99 OMVs for 30 min to 72 h and analyzed them for the presence of OMVs and the respective toxins by immunoblot. The quality of the subcellular fractions and the lack of cross-contamination were verified by the detection of compartment-specific marker proteins (S11A and S11B Fig).

**Fig 4:**
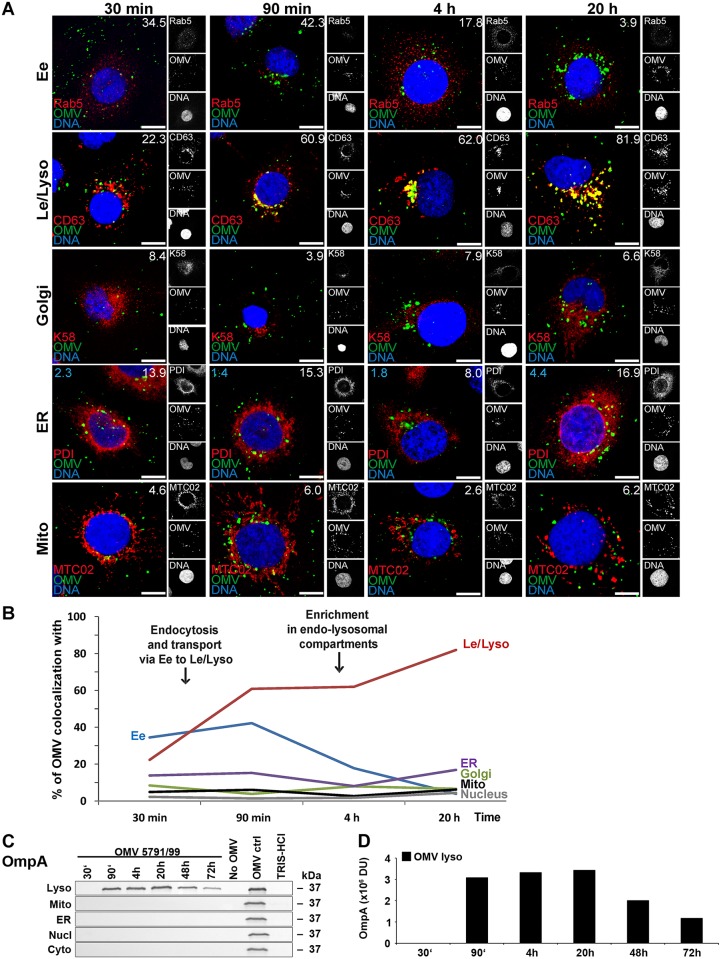
Intracellular trafficking of O157 OMVs. (A) CLSM of HBMEC preincubated with OMVs 5791/99 for 30 min at 4°C, and postincubated at 37°C for 30 min to 20 h. The indicated single fluorescence channels are shown in the right panels (enlargements are displayed in S12 and S13 Figs) and the merged images in the left panels (green, OMVs; red, compartment-specific marker proteins; blue, nuclei; yellow, colocalized green and red signals). The percentages of OMV colocalizations with compartment-specific marker proteins (white numbers) and with nucleus (blue numbers in panels ER) were calculated with the BioImageXD6 tool. Scale bars are 10 μm. (B) Graphical summary of CLSM data shown in A. (Means of colocalizations from at least five different samples are shown in A and B; for standard deviations and significance analysis see S8B Fig). (C) Immunoblot detection of OMVs (anti-OmpA antibody) in isolated subcellular fractions of HBMEC which were postincubated for the times indicated with 5791/99 OMVs or for 72 h without OMVs or with TRIS-HCl OMV buffer (negative controls); 5791/99 OMVs without cells (OMV ctrl) were a positive control. (D) Densitometric quantification of OmpA lysosomal signals shown in C. Ee, early endosomes; Le/Lyso, late endosomes/lysosomes; ER, endoplasmic reticulum; Mito, mitochondria; Nucl, nucleus; Cyto, cytosol; DU, densitometric unit.

The 5791/99 OMVs increasingly colocalized with early endosomes between 30 min and 90 min (colocalization rates 34.5% and 42.3%, respectively), and with late endosomes/lysosomes between 90 min and 20 h of incubation (colocalization rates 60.9%–81.9%) (Fig 4A and 4B and S8B Fig). No significant OMV colocalizations with the Golgi complex, endoplasmic reticulum, mitochondria, and nucleus were observed during 20 h (Fig 4A and 4B and S8B Fig). In accordance with the CLSM data, slightly increasing OmpA signals indicative of OMVs were detected in lysosomal fractions between 90 min and 20 h by immunoblotting (Fig 4C and 4D). OMVs were still present in lysosomes, in decreasing amounts, after 48 h and 72 h (Fig 4C and 4D), but they were absent from all the other subcellular compartments during the whole experiment (Fig 4C). Together, these data demonstrate that after endocytosis by HBMEC, 5791/99 OMVs follow the endocytic pathway from early endosomes to lysosomes where they accumulate and are apparently degraded during time.

### Intracellular trafficking of OMV-delivered Stx2a

**Fig 5:**
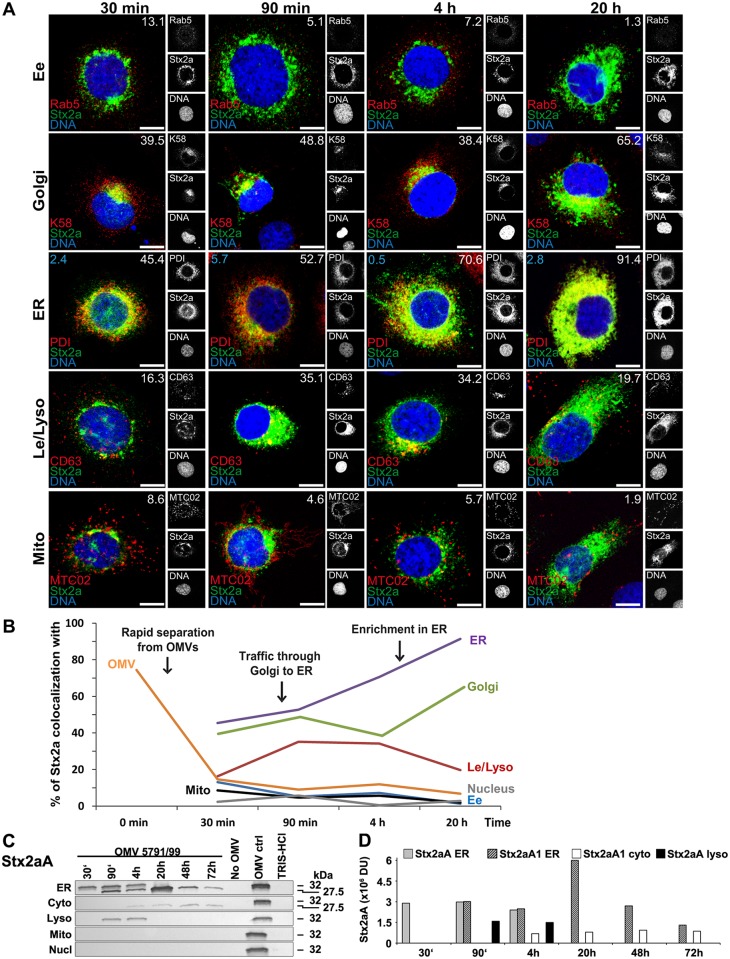
Intracellular trafficking of OMV O157-delivered Stx2a. (A) CLSM of HBMEC preincubated with OMVs 5791/99 for 30 min at 4°C, and postincubated at 37°C for 30 min to 20 h. The indicated single fluorescence channels are shown in the right panels (enlargements are displayed in S15 and S16 Figs) and the merged images in the left panels (green, Stx2a; red, compartment-specific marker proteins; blue, nuclei; yellow, colocalized green and red signals). The percentages of Stx2a colocalizations with compartment-specific marker proteins (white numbers) and with nucleus (blue numbers in panels ER) were calculated with the BioImageXD6 tool. Scale bars are 10 μm. (B) Graphical summary of Stx2a colocalizations with subcellular compartments based on CLSM data shown in A, and with OMVs (based on data shown in Fig 3A). (Means of colocalizations from at least five different samples are shown in A and B; for standard deviations and significance analysis see S8C Fig). (C) Immunoblot detection of Stx2a A subunit in isolated subcellular fractions of HBMEC which were postincubated for the times indicated with 5791/99 OMVs or for 72 h without OMVs or with TRIS-HCl OMV buffer (negative controls); 5791/99 OMVs without cells (OMV ctrl) were a positive control. Molecular weights of Stx2a A subunit (32 kDa) and Stx2a A1 fragment (27.5 kDa) are shown on the right side. (D) Densitometric quantification of Stx2a A/A1 signals shown in C. For abbreviations see legend to Fig 4.

After its rapid separation from OMVs (Figs 3A, 3B and 5B), Stx2a colocalized, in a time-dependent manner, between 30 min and 90 min of incubation with the Golgi complex (colocalization rates 39.5% and 48.8%, respectively), and between 30 min and 20 h with the endoplasmic reticulum (colocalization rates 45.4%–91.4%) (Fig 5A and 5B and S8C Fig). Moreover, a transient mild colocalization of Stx2a with late endosomes/lysosomes was observed after 90 min and 4 h, but there was no colocalization with mitochondria and the nucleus during 20 h (Fig 5A and 5B and S8C Fig). Although the colocalization of OMV-separated Stx2a with early endosomes is low after 30 min (13.1%) (Fig 5A and 5B), the toxin association with OMVs at earlier times (Fig 3A, panel 15 min; colocalization rate 41.3%), when endocytosed OMVs are trafficked via early endosomes (Fig 4A and 4B) suggests that early endosomes are the compartment where Stx2a separates from OMVs before its retrograde transport to the Golgi complex and the endoplasmic reticulum. Immunoblot analyses of isolated subcellular compartments identified two Stx2aA immunoreactive bands in the endoplasmic reticulum (Fig 5C) corresponding by sizes to the intact Stx2a A subunit (~32 kDa) and the enzymatically active A1 fragment (~27.5 kDa), which results from furin-mediated A subunit cleavage [38]. The gradual appearance of the A1 fragment in the endoplasmic reticulum between 90 min and 20 h (Fig 5C and 5D) is consistent with a gradual cleavage of Stx2a A subunit after Stx2a separation from OMVs. The A1 fragment was detected, in low amounts, in the cytosolic fraction between 4 h and 72 h of incubation (Fig 5C and 5D) suggesting that a subset of the cleaved A1 fragment was released in the endoplasmic reticulum and translocated to the cytosol. The Stx2a A subunit was also found in lysosomal fractions after 90 min and 4 h of incubation (Fig 5C and 5D), but it was absent from mitochondrial and nuclear fractions in all time points (Fig 5C). The Stx2a B subunit was identified by immunoblotting in the endoplasmic reticulum between 30 min and 72 h, and in lysosomal fractions after 90 min and 4 h (S14A and S14B Fig). It was absent from all the other compartments during the whole experiment (S14A Fig). Altogether, these data indicate that after its separation from OMVs in early endosomes, Stx2a holotoxin follows a retrograde transport via the Golgi complex to the endoplasmic reticulum, from where a portion of the enzymatically active A1 fragment is translocated to the cytosol to reach its target structures, the ribosomes. A subset of Stx2a, which did not separate from OMVs, is transported with OMVs to lysosomes, likely for degradation.

To gain insight into the mechanism(s) involved in the separation of Stx2a from OMVs in early endosomes we tested the effect of pH. After exposure of 5791/99 OMVs to a pH range from 8.0 to 5.0, a pH-dependent separation of Stx2a occurred. It started at pH 6.5 and reached the maximum at pH 6.0 (S17A Fig), the pH range encountered in early endosomes [39]. This suggested that the slight pH drop in early endosomes facilitates the separation of Stx2a from OMVs in target cells. Accordingly, pretreatment of HBMEC with bafilomycin A1, which inhibits endosomal acidification by inhibiting the vacuolar-type H^+^-ATPase, largely reduced Stx2a trafficking to the endoplasmic reticulum (S17B Fig).

### Globotriaosylceramide (Gb3) associated with detergent-resistant membranes is required for retrograde transport of OMV-delivered Stx2a

**Fig 6:**
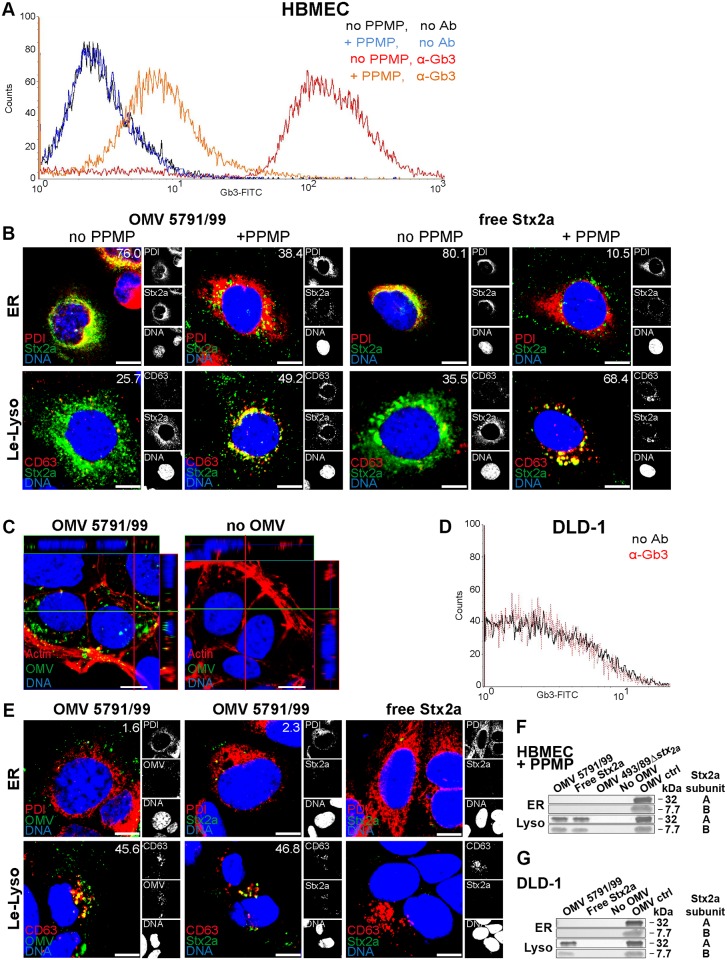
The role of Gb3 in the uptake and retrograde transport of OMV-delivered Stx2a. (A, D) FACS analysis of Gb3 content in (A) PPMP-untreated (no PPMP) and PPMP-treated (+PPMP) HBMEC and (D) DLD-1 cells stained with fluorescein isothiocyanate (FITC)-conjugated anti-CD77/Gb3 antibody or unstained (control). One representative experiment is shown for each cell line (geometric mean fluorescence ± standard deviations from three independent experiments are shown in S18A and S18B Fig). (B, E) CLSM analysis of trafficking of OMV-delivered and free Stx2a (control) into the endoplasmic reticulum (ER) and late endosomes/lysosomes (Le-Lyso) in (B) PPMP-untreated and PPMP-treated HBMEC (20 h) and (E) DLD-1 cells (4 h). The indicated single fluorescence channels are shown in the right panels (enlargements are displayed in S19 Fig) and the merged images in the left panels (green, Stx2a or OMVs, as indicated; red, PDI or CD63, as indicated; blue, nuclei; yellow, colocalized green and red signals). The percentages of colocalizations of the respective signals (white numbers) were calculated with the BioImageXD6 tool (means of colocalizations from three different samples are shown). (C) Uptake of 5791/99 OMVs by DLD-1 cells after 4 h of incubation. Untreated cells (no OMV) were a negative control. Green, OMVs; red, actin; blue, nuclei. Confocal Z-stack projections are included at upper/right sides. Crosshairs show the position of the xy and yz planes. Scale bars in all panels are 10 μm. (F, G) Immunoblot detection of Stx2a in isolated endoplasmic reticulum (ER) and lysosomal (Lyso) fractions of (F) PPMP-treated HBMEC and (G) DLD-1 cells after 20 h and 4 h of incubation, respectively, with the indicated samples or left untreated (no OMV); 5791/99 OMVs without cells (OMV ctrl) were a positive control.

The association with its receptor Gb3 clustered within membrane microdomains termed lipid rafts or detergent-resistant membranes (DRMs) is required for the retrograde transport of StxB and free purified Stx into the Golgi complex and the endoplasmic reticulum, and for Stx-mediated cytotoxicity [40–42]. To determine if OMV-delivered Stx2a requires DRM-associated Gb3 for its retrograde transport, we first analyzed Stx2a trafficking in HBMEC where the content of Gb3, which is predominantly associated with DRMs [43], had been significantly reduced (to ~6% of that in control cells) by treatment with the glucosylceramide synthase inhibitor PPMP (1-phenyl-2-hexadecanoyl-amino-3-morpholino-1-propanol). This was demonstrated by FACS analysis (Fig 6A and S18A Fig) and visualized by CLSM (S18C Fig). In contrast to PPMP-untreated HBMEC, where OMV-delivered Stx2a strongly colocalized with the endoplasmic reticulum after 4 h and 20 h of incubation (Figs 5A and 6B), in PPMP-treated cells most of the toxin was retained in the endosomal pathway and colocalized with late endosomes/lysosomes after 20 h (Fig 6B and S18E and S18H Fig). The same was observed for free purified Stx2a used as a control (Fig 6B and S18E and S18H Fig). The PPMP-mediated shift of Stx2a trafficking from the endoplasmic reticulum to the lysosomes was confirmed by immunoblotting of the isolated fractions (Fig 6F; compare with Fig 5C, 20 h).

To confirm the requirement of Gb3 for the retrograde transport of OMV-delivered Stx2a, we used the intestinal epithelial cell line DLD-1, which was reported to contain no detectable Gb3 [44]. This was verified in our study by FACS and CLSM analyses (Fig 6D and S18B and S18D Fig). OMVs 5791/99 were internalized by DLD-1 cells (Fig 6C) and were trafficked to lysosomes (Fig 6E, first set of panels). Stx2a was detected in lysosomes, but not in the endoplasmic reticulum, after 4 h of incubation by both CLSM (Fig 6E, second set of panels) and immunoblot (Fig 6G). Free Stx2a (control) was found neither in lysosomes nor in the endoplasmic reticulum (Fig 6E and 6G), which is in agreement with the reported lack of StxB internalization by DLD-1 cells [44]. Altogether, these experiments demonstrated that Gb3 is not required for the cellular uptake of OMV-associated-Stx2a, but it is essential for directing the OMV-delivered Stx2a into the retrograde trafficking pathway.

**Fig 7:**
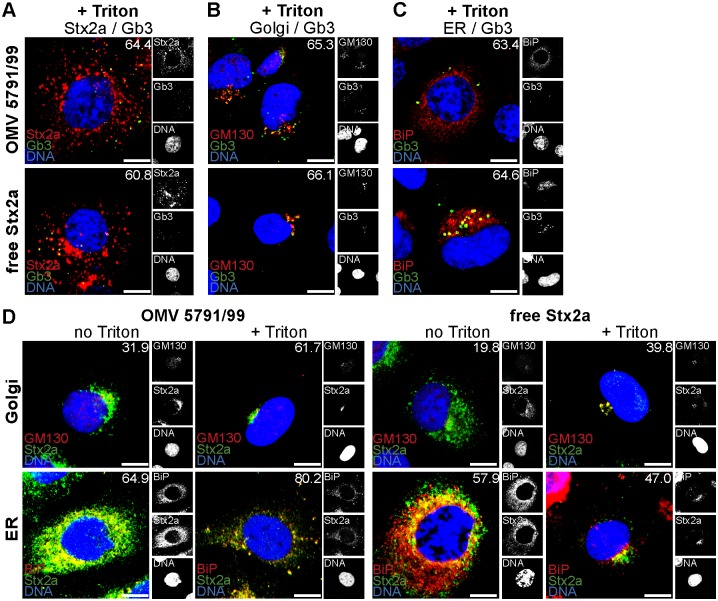
Retrograde transport of OMV-delivered Stx2a involves the toxin´s interactions with DRM-associated Gb3. (A, B, C) Colocalization of Stx2a with Gb3 (A), and colocalization of Gb3 with the Golgi marker GM130 (B), and the endoplasmic reticulum marker BiP (C) in cells exposed to 5791/99 OMVs or free Stx2a (control) for 4 h (A, C) or 90 min (B) and processed for CLSM after extraction with a Triton X-100-containing buffer (1 min on ice). Green, Gb3; red, Stx2a (A) or GM130 (B) or BiP (C); blue, nuclei; yellow, colocalized green and red signals. (D) Colocalization of OMV-delivered Stx2a and free Stx2a (control) with the Golgi complex (90 min) and the endoplasmic reticulum (4 h) in HBMEC untreated (no Triton) or pretreated with Triton X-100 (+Triton) before being processed for CLSM. Green, Stx2a; red, GM130 or BiP, as indicated; blue, nuclei; yellow, colocalized green and red signals. In all pictures, the indicated single fluorescence channels are shown in the right panels (enlargements are displayed in S20 Fig) and the merged images in the left panels. The percentages of colocalizations of the respective signals (white numbers) were calculated with the BioImageXD6 tool (means of colocalizations from three different samples are shown). Scale bars are 10 μm.

To determine if the DRM-associated Gb3 pool [40, 41] is involved in the retrograde transport of OMV-delivered Stx2a in HBMEC, we analyzed by CLSM: i) the interaction of OMV-delivered Stx2a with DRM-associated Gb3, ii) the presence of DRM-associated Gb3 within the Golgi complex and the endoplasmic reticulum of cells exposed to 5791/99 OMVs, and iii) the association of OMV-delivered Stx2a with DRMs in these compartments during intracellular trafficking. To this end, HBMEC were incubated with 5791/99 OMVs or free Stx2a (used as a control) for 90 min or 4 h and processed for CLSM either directly or after extraction with a detergent (Triton X-100)-containing buffer [40]. The OMV-delivered Stx2a as well as free Stx2a strongly colocalized with Gb3 in Triton X-100-treated cells (colocalization rates 64.4% and 60.8%, respectively) (Fig 7A) indicating that the toxin interacts with DRM-associated Gb3. Moreover, Gb3 strongly colocalized in Triton X-100-extracted cells with the Golgi marker GM130 (Fig 7B) and the endoplasmic reticulum marker BiP (Fig 7C), both of which were reported to be associated with DRMs [40, 45]; this demonstrated the presence of DRM-associated Gb3 in these compartments. Finally, both OMV-delivered Stx2a and free Stx2a colocalized with the Golgi complex (after 90 min) and the endoplasmic reticulum (after 4 h) in a Triton X-100-resistant manner (Fig 7D). Altogether, these experiments demonstrated that the retrograde transport of OMV-delivered Stx2a in HBMEC involves its interactions with DRM-associated Gb3 in the Golgi complex and the endoplasmic reticulum.

### Intracellular trafficking of OMV-delivered CdtV-A, CdtV-B, and CdtV-C

**Fig 8:**
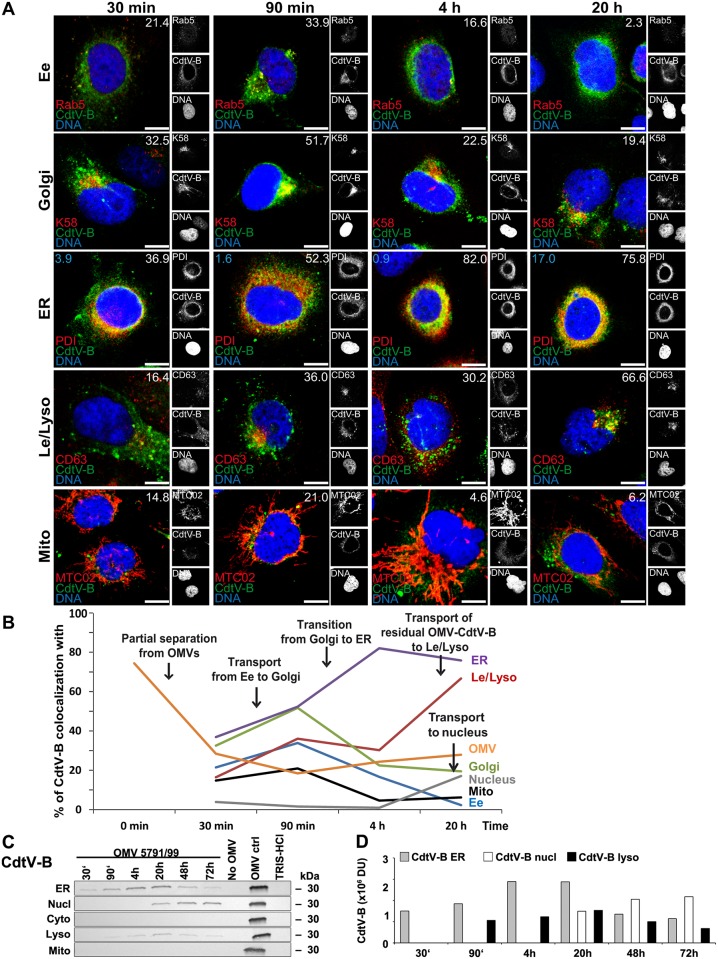
Intracellular trafficking of OMV O157-delivered CdtV-B. (A) CLSM of HBMEC preincubated with OMVs 5791/99 for 30 min at 4°C, and postincubated at 37°C for 30 min to 20 h. The indicated single fluorescence channels are shown in the right panels (enlargements are displayed in S21 and S22 Figs) and the merged images in the left panels (green, CdtV-B; red, compartment-specific marker proteins; blue, nuclei; yellow, colocalized green and red signals). The percentages of CdtV-B colocalizations with compartment-specific marker proteins (white numbers) and with nucleus (blue numbers in panels ER) were calculated with the BioImageXD6 tool. Scale bars are 10 μm. (B) Graphical summary of CdtV-B colocalizations with subcellular compartments based on CLSM data shown in A, and with OMVs (based on data shown in Fig 3A). (Means of colocalizations from at least five different samples are shown in A and B; for standard deviations and significance analysis see S8D Fig). (C) Immunoblot detection of CdtV-B in isolated subcellular fractions of HBMEC which were postincubated for the times indicated with 5791/99 OMVs, or for 72 h without OMVs or with TRIS-HCl OMV buffer (negative controls); 5791/99 OMVs without cells (OMV ctrl) were a positive control. (D) Densitometric quantification of CdtV-B signals shown in C. For abbreviations see legend to Fig 4.

As suggested by their different associations with OMVs during intracellular trafficking (Fig 3A and 3B), the A, B, and C subunits of the CdtV holotoxin utilized different trafficking pathways. Based on CLSM analyses, the enzymatically active CdtV-B subunit was transported, after its separation from OMVs (Figs 3A, 3B and 8B), to the Golgi complex (colocalization 32.5%–51.7% between 30 min and 90 min) and further to the endoplasmic reticulum (colocalization 52.3%–82% between 90 min and 4 h, with a plateau until 20 h) (Fig 8A and 8B and S8D Fig). No obvious CdtV-B colocalization with the nucleus, its target structure, was observed until 4 h, but a significant increase (to 17%) occurred after 20 h (Fig 8A and 8B and S8D Fig). Immunoblot analyses of subcellular fractions identified CdtV-B in the endoplasmic reticulum between 30 min and 72 h, with a time-dependent increase until 20 h followed by a decrease until 72 h (Fig 8C and 8D). The peak in the endoplasmic reticulum after 20 h correlated with the appearance of CdtV-B in the nucleus, where the CdtV-B amount steadily increased until 72 h, while decreasing in the endoplasmic reticulum (Fig 8C and 8D). No CdtV-B was found in the cytosol during the whole experiment (Fig 8C). Weak CdtV-B signals were detected in lysosomal fractions between 90 min and 72 (Fig 8C and 8D), which is in accordance with CdtV-B detection in lysosomes between 90 min and 20 h using CLSM (Fig 8A and 8B and S8D Fig). Taken together, the CLSM and immunoblot analyses demonstrated that the OMV-delivered CdtV-B follows two different trafficking pathways, likely depending on its separation from OMVs after internalization. The major part of CdtV-B, which separates from OMVs (Figs 3A, 3B and 8B), is trafficked via the Golgi complex to the endoplasmic reticulum from where it is translocated to its target organelle, the nucleus (Fig 8A–8D). A residual subset of CdtV-B which remains OMV-associated (Figs 3A, 3B and 8B) is trafficked to lysosomes where it is apparently degraded during time (Fig 8A–8D). The observation that most of CdtV-B separates from OMVs within 30 min after internalization (Figs 3A, 3B and 8B and S8A Fig) when OMVs are located in early endosomes (Fig 4A and 4B) suggests that similar to Stx2a, the separation of CdtV-B from OMVs takes place in this compartment. Moreover, like with Stx2a, the CdtV-B separation from OMVs in vitro occurred at the pH range between 6.5 and 6.0 (S17A Fig), suggesting the role of the early endosomal pH drop in this process in host cells. This was supported by the ability of bafilomycin A1 to substantially reduce the CdtV-B trafficking to the endoplasmic reticulum in HBMEC (S17B Fig).

**Fig 9:**
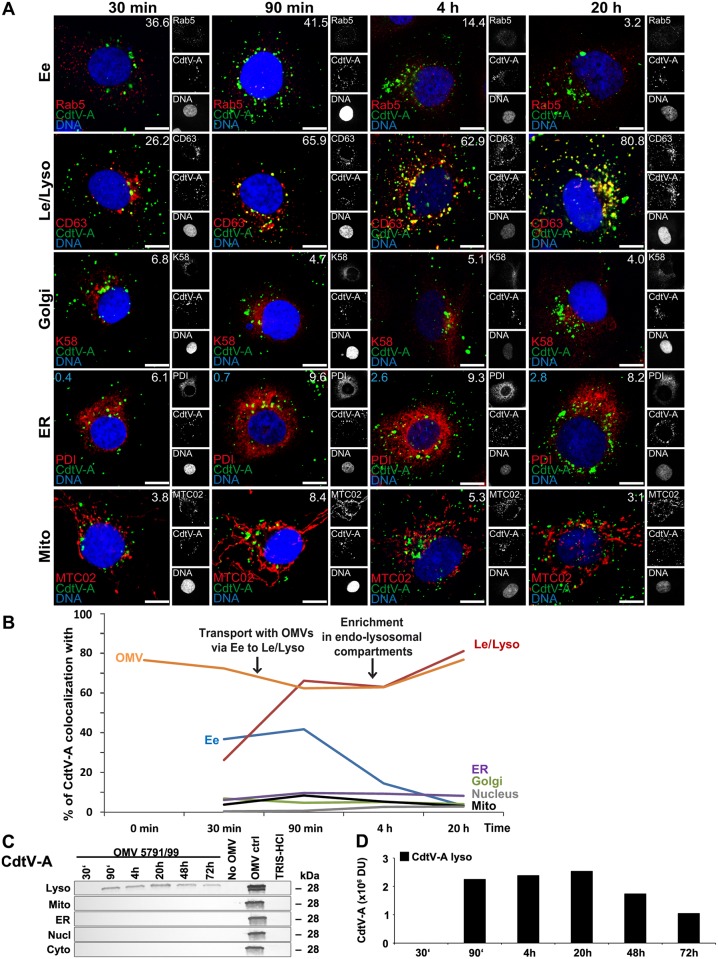
Intracellular trafficking of OMV O157-delivered CdtV-A. (A) CLSM of HBMEC preincubated with OMVs 5791/99 for 30 min at 4°C, and postincubated at 37°C for 30 min to 20 h. The indicated single fluorescence channels are shown in the right panels (enlargements are displayed in S23 and S24 Figs) and the merged images in the left panels (green, CdtV-A; red, compartment-specific marker proteins; blue, nuclei; yellow, colocalized green and red signals). The percentages of CdtV-A colocalizations with compartment-specific marker proteins (white numbers) and with nucleus (blue numbers in panels ER) were calculated with the BioImageXD6 tool. Scale bars are 10 μm. (B) Graphical summary of CdtV-A colocalizations with subcellular compartments based on CLSM data shown in A, and with OMVs (based on data shown in Fig 3A). (Means of colocalizations from at least three different samples are shown in A and B; for standard deviations and significance analysis see S8E Fig). (C) Immunoblot detection of CdtV-A in isolated subcellular fractions of HBMEC which were postincubated for the times indicated with 5791/99 OMVs or for 72 h without OMVs or with TRIS-HCl OMV buffer (negative controls); 5791/99 OMVs without cells (OMV ctrl) were a positive control. (D) Densitometric quantification of CdtV-A signals shown in C. For abbreviations see legend to Fig 4.

**Fig 10:**
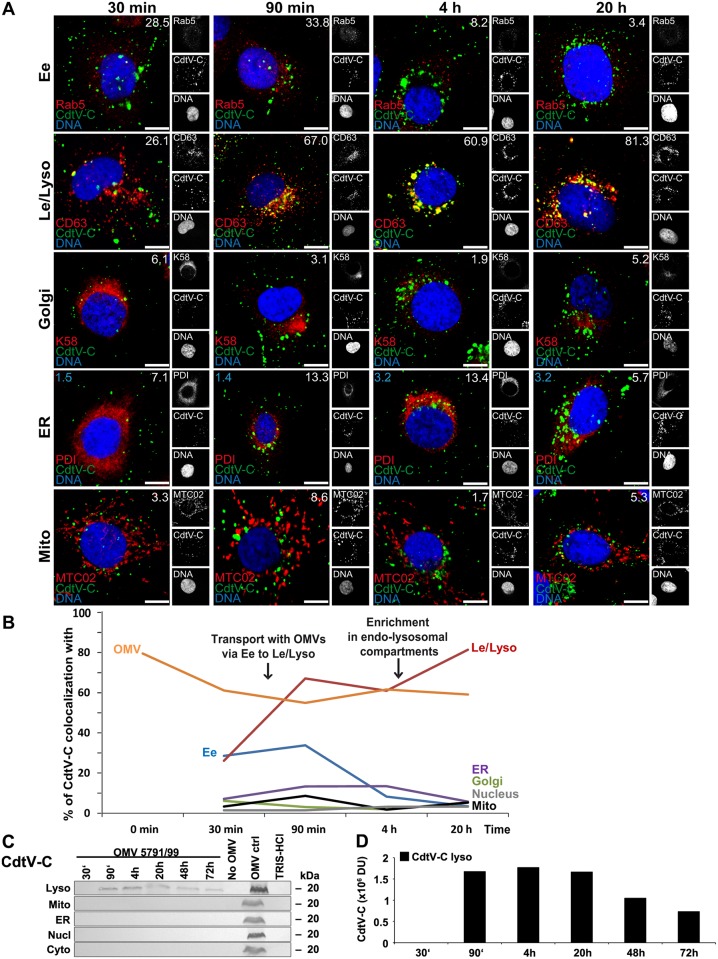
Intracellular trafficking of OMV O157-delivered CdtV-C. (A) CLSM of HBMEC preincubated with OMVs 5791/99 for 30 min at 4°C, and postincubated at 37°C for 30 min to 20 h. The indicated single fluorescence channels are shown in the right panels (enlargements are displayed in S25 and S26 Figs) and the merged images in the left panels (green, CdtV-C; red, compartment-specific marker proteins; blue, nuclei; yellow, colocalized green and red signals). The percentages of CdtV-C colocalizations with compartment-specific marker proteins (white numbers) and with nucleus (blue numbers in panels ER) were calculated with the BioImageXD6 tool. Scale bars are 10 μm. (B) Graphical summary of CdtV-C colocalizations with subcellular compartments based on CLSM data shown in A, and with OMVs (based on data shown in Fig 3A). (Means of colocalizations from at least three different samples are shown in A and B; for standard deviations and significance analysis see S8F Fig). (C) Immunoblot detection of CdtV-C in isolated subcellular fractions of HBMEC which were postincubated for the times indicated with 5791/99 OMVs, or for 72 h without OMVs or with TRIS-HCl OMV buffer (negative controls); 5791/99 OMVs without cells (OMV ctrl) served as a positive control. (D) Densitometric quantification of CdtV-C signals shown in C. For abbreviations see legend to Fig 4.

The CdtV-A and CdtV-C subunits, which remained mostly associated with OMVs after intracellular delivery (Figs 3A, 3B, 9B and 10B), were trafficked, as demonstrated by CLSM, via early endosomes to late endosomes/lysosomes where they accumulated between 90 min and 20 h (colocalization rates 65.9%–80.8%, and 67%–81.3%, respectively) (Figs 9A, 9B, 10A and 10B and S8E and S8F Fig). There were no obvious CdtV-A or CdtV-C colocalizations with the Golgi complex, endoplasmic reticulum, mitochondria and nucleus during 20 h (Figs 9A, 9B, 10A and 10B and S8E and S8F Fig). In accordance with CLSM, immunoblot analyses identified both CdtV-A and CdtV-C solely in lysosomal fractions, where their amounts slightly increased (CdtV-A) or remained stable (CdtV-C) between 90 min and 20 h and subsequently decreased until 72 h (Figs 9C, 9D, 10C and 10D). The kinetics of the CdtV-A and CdtV-C appearance in early endosomes and lysosomes (Figs 9B–9D and 10B–10D), was very similar to that of OMVs (Fig 4B–4D). These data demonstrate that CdtV-A and CdtV-C subunits are trafficked, together with OMVs, via early endosomes to lysosomes where they are likely degraded.

### Retrograde transport of OMV-delivered CdtV-B does not require CdtV-A and CdtV-C

**Fig 11:**
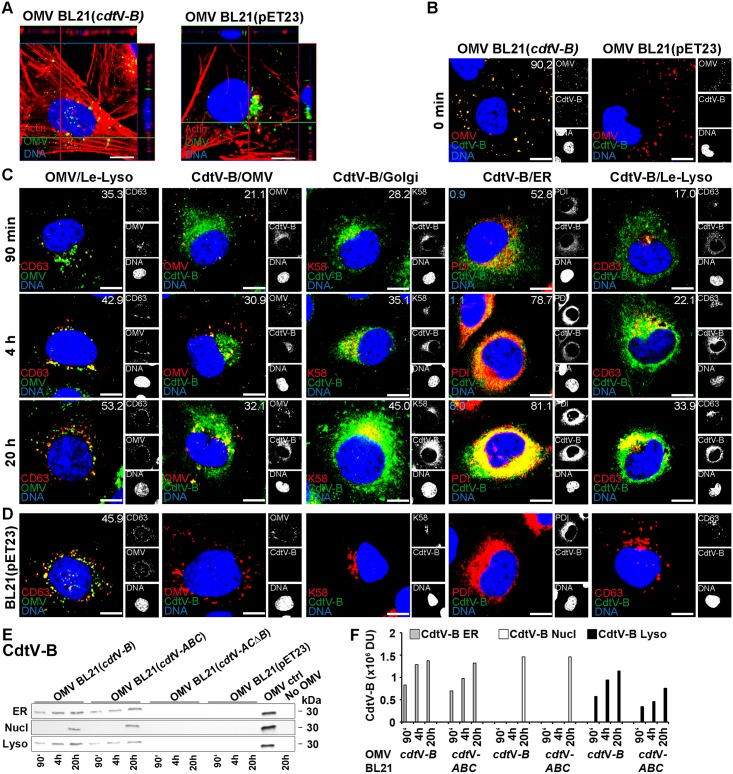
Retrograde transport of OMV-delivered CdtV-B does not require CdtV-A and CdtV-C. HBMEC were preincubated (30 min, 4°C) with recombinant CdtV-B-containing OMVs from strain BL21(*cdt*V-*B*) or with CdtV-B-lacking OMVs from BL21(pET23) vector control and postincubated at 37°C for 90 min to 20 h. (A) OMV uptake was determined by CLSM after 4 h. Green, OMVs; red, actin; blue, nuclei. Confocal Z-stack projections are included at upper/right sides. Crosshairs show the position of the xy and yz planes. (B, C) Trafficking of BL21(*cdt*V-*B*) OMVs and OMV-delivered CdtV-B, and separation of CdtV-B from the vesicles during the trafficking were determined by CLSM. (B) Colocalization of CdtV-B with OMVs upon OMV cellular binding (time 0 min). (C) Colocalizations of BL21(*cdt*V-*B*) OMVs with late endosomes/lysosomes (panels OMV/Le-Lyso), of CdtV-B with OMVs (panels CdtV-B/OMV), and of CdtV-B with the indicated subcellular compartments after postincubation at 37°C for 90 min to 20 h. (D) CLSM of control cells postincubated for 20 h with BL21(pET23) OMVs. The indicated single fluorescence channels in B to D are shown in the right panels (enlargements are displayed in S29 and S30 Figs) and the merged images in the left panels (green, OMV or CdtV-B, as indicated; red, OMV or compartment-specific marker proteins, as indicated; blue, nuclei; yellow, colocalized green and red signals). The percentages of colocalizations of the respective signals (white numbers) were calculated with the BioImageXD6 tool; blue numbers in panels ER indicate colocalization of CdtV-B with nucleus (means of colocalizations from three different samples are shown). Scale bars in A to D are 10 μm. (E) Immunoblot detection of CdtV-B in isolated subcellular fractions of HBMEC which were postincubated with the indicated OMVs for 90 min to 20 h. BL21(*cdt*V-*ABC*) OMVs were a positive control and BL21(*cdt*V-*ACΔB*) OMVs, BL21(pET23) OMVs and untreated cells (no OMV) negative controls; lanes OMV ctrl contain 5791/99 OMVs without cells. (F) Densitometric quantification of CdtV-B signals shown in E. For abbreviations see legend to Fig 4.

The lysosomal trafficking and degradation of CdtV-A and CdtV-C subunits (Figs 9A–9D and 10A–10D) raised a question whether or not they are required for the retrograde transport of OMV-delivered CdtV-B. To gain insight into this issue, we cloned the *cdt*V*-A*, *cdt*V*-B*, and *cdt*V*-C* genes (for the clones see S1 Table), isolated OMVs containing the recombinant subunit proteins (S27A and S27B Fig), and compared the intracellular trafficking of OMV-delivered single subunits with that of the respective subunits delivered within CdtV holotoxin. OMVs from a *cdt*V-*B* deletion mutant and from *E*. *coli* BL21 carrying the cloning vector served as controls. OMVs from all recombinant strains, regardless if and which CdtV subunit they contained, were internalized by HBMEC after 4 h of incubation (Fig 11A and S28A Fig). As expected, the OMVs were trafficked into late endosomes/lysosomes where they remained until 20 h (Fig 11C and 11D and S28B and S28C Fig). The recombinant CdtV-B delivered via BL21(*cdt*V-*B*) OMVs mostly separated from OMVs after internalization (compare Fig 11B and 11C, panels CdtV-B/OMV), and was retrogradely transported to the Golgi complex and the endoplasmic reticulum, reaching the nucleus after 20 h (Fig 11C). Moreover, partial colocalization of CdtV-B with late endosomes/lysosomes was observed between 90 min and 20 h (Fig 11C), which presumably resulted from its incomplete separation from OMVs (Fig 11C). The CdtV-B trafficking determined by CLSM was confirmed by immunoblot analysis of isolated subcellular fractions (Fig 11E and 11F). No CdtV-B signals were observed in cells exposed to OMVs from the *cdt*V-*B* deletion mutant BL21(*cdt*V-*ACΔB*) which lack CdtV-B (S27A Fig) or OMVs from the vector control BL21(pET23) (Fig 11D and 11E and S28D Fig) confirming the specificity of the CdtV-B signals in cells exposed to BL21(*cdt*V-*B*) OMVs (Fig 11C and 11E). The intracellular trafficking of the OMV-delivered CdtV-B alone was similar to that of the CdtV-B subunit delivered into cells as a part of wild-type (Fig 8A and 8C) or recombinant (Fig 11E and S28D Fig) CdtV holotoxin. The OMV-delivered recombinant CdtV-A and CdtV-C, expressed either separately (strains BL21(*cdt*V-*A*) and BL21(*cdt*V-*C*), respectively) or together (strain BL21(*cdt*V-*ACΔB*)), were solely detected in lysosomes during 20 h of incubation using both CLSM and immunoblot (S28B, S28C, S28E and S28F Fig), similar to these subunits delivered within CdtV holotoxin (Figs 9A, 9C, 10A and 10C and S28C, S28E and S28F Fig). This was in agreement with the lack of separation of the recombinant CdtV-A and CdtV-C from OMVs until 20 h (S28B Fig, panels CdtV/OMV), as also observed for the holotoxin-associated CdtV-A and CdtV-C (Fig 3A and 3B). These experiments demonstrated that OMV-delivered CdtV-B can be retrogradely transported in the absence of CdtV-A and CdtV-C indicating that the latter subunits are not essential for this process.

### Intracellular trafficking of OMV-delivered EHEC-Hly

Similar to our previous observation for EHEC-Hly associated with non-O157 OMVs [10], EHEC-Hly delivered into HBMEC via OMVs from EHEC O157:H7 strain 5791/99 was trafficked via early endosomes to late endosomes/lysosomes, where it separated from OMVs, escaped from the lysosomes, and was transported to mitochondria (S31A–S31D and S8G Figs).

### OMV-delivered CdtV activates DNA damage signaling cascade and causes G2 cell cycle arrest

**Fig 12:**
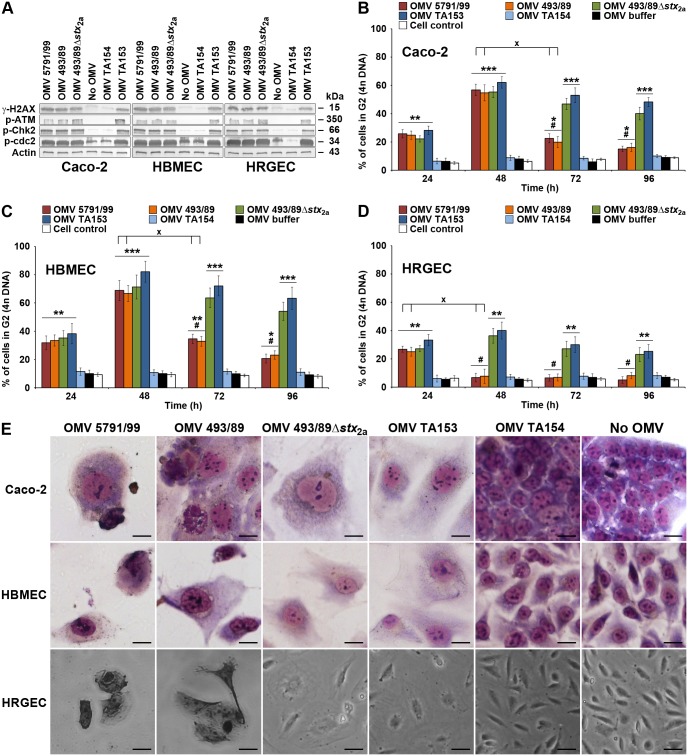
OMV-delivered CdtV triggers DNA damage signaling and G2 arrest in epithelial and microvascular endothelial cells. (A) Activation of DNA damage G2 checkpoint signaling in Caco-2 cells, HBMEC and HRGEC incubated with O157, TA153 (CdtV-positive control), or TA154 (vector control) OMVs for 20 h or left untreated (no OMV) as demonstrated by immunoblotting of cell lysates with antibodies against phosphorylated forms of the indicated proteins. Actin served as a loading control. (B, C, D) G2 arrest induced in the indicated cell cultures by O157, TA153, or TA154 OMVs during 24 h to 96 h demonstrated by flow cytometric detection of cells with 4n DNA content. Untreated cells and cells treated with OMV buffer (20 mM TRIS-HCl, pH 8.0) were negative controls. Data are means ± standard deviations from three independent experiments. *p < 0.05, **p < 0.01 or ***p < 0.001 for G2 arrest caused by the indicated OMVs compared to OMV buffer; ^#^*p* < 0.01 for G2 arrest caused by Stx2a-containing versus Stx2a-lacking OMVs; ^×^*p* < 0.01 for G2 arrest caused by 5791/99 and 493/89 OMVs after 24 h compared to 48 h (HRGEC), or after 48 h compared to 72 h (Caco-2, HBMEC). Calculations were performed using one-way ANOVA. (E) Cell distension after 72 h of incubation with CdtV-containing O157 or TA153 OMVs; cells treated with CdtV-negative TA154 OMVs and untreated cells (no OMV) were negative controls. Cells were stained with Giemsa (Caco-2, HBMEC) or photographed native (HRGEC). Scale bars are 20 μm. Note the presence of apoptotic cells (condensed nuclei, reduced cytoplasm) in cultures treated with Stx2a-containing OMVs (5791/99, 493/89).

To determine if the OMV-delivered virulence factors are biologically active we first focused on CdtV, which was present in OMVs of all three *E*. *coli* O157 strains (Fig 1B). The Cdt cytotoxicity results from CdtB-mediated DNA double-strand breaks, which activate DNA damage checkpoint responses that arrest the cell cycle in G1 or G2 phase [46, 47]. The G2 checkpoint signaling starts with activation of the ataxia telangiectasia-mutated (ATM) protein kinase, the central DNA damage sensor [46, 47], which triggers a downstream phosphorylation cascade resulting in activation of protein kinase Chk2, inactivation of Cdc25C phosphatase, and, eventually, accumulation of hyperphosphorylated (inactive) cyclin-dependent kinase cdc2 [46, 47]; the last event directly causes G2 arrest [48]. Formation of DNA double-strand breaks in Caco-2 cells, HBMEC and HRGEC incubated for 20 h with O157 or control TA153 OMVs (all containing ~340 ng/ml of CdtV) was indicated by the detection of phosphorylated histone protein H2AX (γ-H2AX), a marker of DNA double-strand breaks [49] (Fig 12A). Accordingly, ATM, Chk2, and cdc2 were phosphorylated in all cell types exposed to these OMVs (Fig 12A) demonstrating that the DNA damage G2 checkpoint response was activated. The cells were arrested in the G2 phase, as demonstrated by flow cytometric detection of 4n DNA content (S32A and S32C Fig). The kinetics of the G2 arrest differed in cells treated with OMVs containing CdtV alone or together with Stx2a. The G2 arrest elicited by Stx2a-lacking OMVs (493/89Δ*stx*_2a_, TA153) increased between 24 h and 48 h of incubation and then slowly decreased until 96 h (Fig 12B–12D and S32A and S32C Fig). In contrast, the G2 arrest caused by Stx2a-containing OMVs (5791/99, 493/89) (~460 ng/ml of Stx2a) peaked after 24 h (HRGEC) or 48 h (Caco-2, HBMEC) and then rapidly decreased during the next 24 h, remaining significantly lower than that caused by Stx2a-lacking OMVs up to 96 h (Fig 12B–12D and S32A and S32C Fig). The G2 arrest was dose-dependent; the lowest dose of OMV-associated CdtV capable of eliciting a significant G2 arrest at the time of its peak was 21.25 ng/ml in Caco-2 cells and HBMEC (48 h) and 42.5 ng/ml in HRGEC (24 h) (S33A–S33C Fig). The arrested cells underwent a progressive distension during 72 h of OMV exposure (Fig 12E); in addition to typical giant cells, numerous cells displaying apoptotic morphology (condensed nuclei, reduced cytoplasm) were found in cultures treated with Stx2a-containing OMVs 5791/99 and 493/89 (Fig 12E). No phosphorylation of H2AX and proteins of the G2 checkpoint cascade (Fig 12A), no G2 arrest (Fig 12B–12D and S32B and S32D Fig), and no cell distension (Fig 12E) were elicited by CdtV-negative (S5B Fig) OMVs from TA154 vector control strain indicating that OMV-delivered CdtV caused these effects.

### OMV-delivered CdtV-B subunit is responsible for CdtV-mediated biological effects

**Fig 13:**
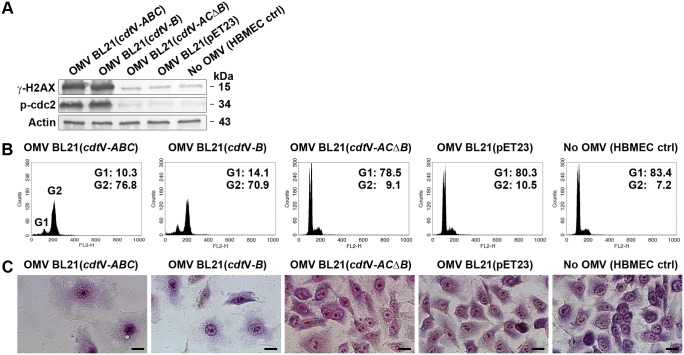
CdtV-B is responsible for the biological effects caused by OMV-delivered CdtV holotoxin. HBMEC were incubated with OMVs from the indicated recombinant strains containing the CdtV holotoxin (BL21(*cdt*V-*ABC*) OMVs), CdtV-B subunit (BL21(*cdt*V-*B*) OMVs), or CdtV-AC subunits (BL21(*cdt*V-*AC*Δ*B*) OMVs). OMVs from BL21(pET23) vector control and untreated cells (no OMV) were negative controls. (A) DNA damage response was determined by immunoblotting of cell lysates with antibodies against phosphorylated forms of H2AX (γ-H2AX) and cdc2 (p-cdc2) after 20 h of exposure. Actin served as a loading control. (B) G2 arrest was measured by flow cytometric detection of cells with 4n DNA content after 48 h of exposure. Positions of the G1 (2n DNA) and G2 (4n DNA) peaks are indicated in the first histogram, and the proportions (%) of cells in the G1 and G2 cell cycle phase, respectively, are shown in all histograms. (C) Cell distension was visualized by light microscopy of Giemsa-stained cells after 72 h of exposure. Scale bars are 20 μm.

We used two different approaches to determine if, like in free Cdts [46, 47], the CdtB subunit is responsible for the biological activity of OMV-associated CdtV holotoxin. First, using OMVs from *cdt*V-*B* deletion mutant BL21(*cdt*V-*ACΔB*), which contain CdtV-A and CdtV-C but not CdtV-B (S27A Fig), we showed that the *cdt*V-*B* deletion abolished the ability of OMV-delivered CdtV to cause the DNA damage response, G2 cell cycle arrest, and cell distension (Fig 13A–13C). Second, CdtV-B subunit alone delivered intracellularly via BL21(*cdt*V-*B*) OMVs was able to reproduce all the biological effects of OMV-delivered CdtV holotoxin (Fig 13A–13C). These experiments demonstrated that CdtV-B is the biologically active component of OMV-associated CdtV.

### CdtV-mediated G2 arrest is followed by apoptosis caused by Stx2a and CdtV

**Fig 14:**
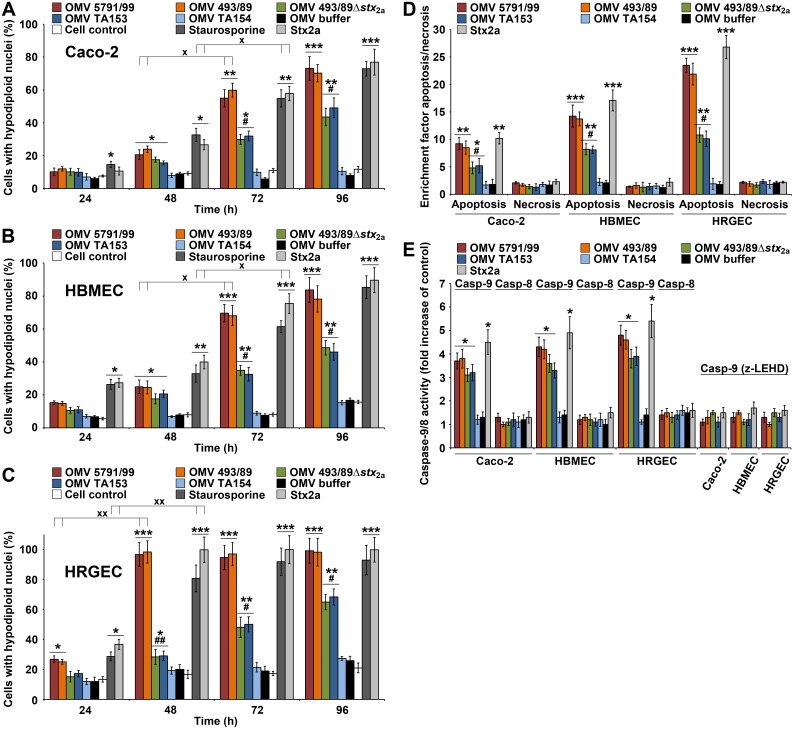
CdtV-mediated G2 arrest is followed by apoptosis induced by Stx2a and CdtV. (A, B, C) Time course of apoptosis induced in (A) Caco-2 cells, (B) HBMEC, and (C) HRGEC by CdtV-containing O157 OMVs possessing (5791/99, 493/89) or lacking (493/89Δ*stx*_2a_) Stx2a, by control CdtV-containing or CdtV-lacking OMVs TA153 or TA154, respectively, and by purified Stx2a (460 ng/ml) as determined by flow cytometric quantification of cells with hypodiploid nuclei. Staurosporine (1 μM) was a positive and OMV buffer and untreated cells negative controls. *p < 0.05, **p < 0.01 or ***p < 0.001 for apoptosis caused by the indicated OMVs, purified Stx2a or staurosporine compared to OMV buffer; ^#^*p* < 0.01 or ^##^*p* < 0.001 for apoptosis caused by Stx2a-lacking versus Stx2a-containing OMVs; ^×^*p* < 0.01 or ^××^*p* < 0.001 for apoptosis caused by OMVs 5791/99, 493/89, purified Stx2a or staurosporine after 48 h compared to 24 h (HRGEC), or after 72 h compared to 48 h (Caco-2, HBMEC). (D) Apoptosis and necrosis caused by O157 OMVs and controls in the indicated cell types after 96 h as determined by the Cell Death Detection ELISA. Enrichment factors were calculated by dividing OD_405_ absorbance values of sample-treated cells with those of untreated cells; *p < 0.05, **p < 0.01 or ***p < 0.001 for apoptosis caused by OMVs or purified Stx2a compared to OMV buffer; ^#^*p* < 0.01 for apoptosis caused by Stx2a-lacking compared to Stx2a-containing OMVs. (E) Activities of caspase-9 and caspase-8 in the indicated cell lysates after 48 h of incubation with O157 OMVs or with positive (purified Stx2a, OMV T153) or negative (TA154 OMVs, OMV buffer) controls determined with colorimetric substrates (LEHD-pNA and IETD-pNA, respectively). The caspase activities in O157 OMV-treated or control-treated cells were expressed as a fold-increase of those in untreated cells. Inhibitor of caspase-9 (z-LEHD-fmk) was added to cells 30 min before samples; *p < 0.05 compared to untreated cells. Data in all panels are means ± standard deviations from three independent experiments; calculations were performed using one-way ANOVA.

The appearance of prominent sub-G1 apoptotic peaks in histograms of HBMEC and Caco-2 cells after 72 h of incubation with Stx2a-containing OMVs 5791/99 or 493/89 (S32A and S32C Fig), which coincides with the rapid drop of G2 arrested cells (Fig 12B and 12C and S32A and S32C Fig) led us to hypothesize that the G2 arrested cells die of apoptosis caused by Stx2a. To test this hypothesis, we determined proportions of apoptotic cells in Caco-2, HBMEC and HRGEC cultures exposed to 5791/99 or 493/89 OMVs for 24 h to 96 h and compared them with those elicited by Stx2a-negative OMVs and purified Stx2a. The 5791/99 and 493/89 OMVs caused apoptosis in each cell type. The proportions of apoptotic cells sharply increased between 24 h and 48 h (HRGEC) or between 48 h and 72 h (Caco-2, HBMEC) and then remained stable (HRGEC) or slightly increased (Caco-2, HBMEC) until 96 h (Fig 14A–14C). The sharp increase of apoptotic cells correlated with the sharp decrease of G2 arrested cells in the respective cultures and time intervals (Fig 12B–12D). A similar time course of apoptosis was elicited by purified Stx2a (460 ng/ml present in 5791/99 and 493/89 OMVs), and by staurosporine, an apoptosis-inducing agent used as a positive control (Fig 14A–14C). The CdtV-positive, Stx2a-negative OMVs (493/89Δ*stx*_2a_, TA153) also caused apoptosis, which increased gradually between 48 h and 96 h and was significantly lower than that caused by the Stx2a-positive OMVs between 48 h and 96 h in HRGEC and between 72 h and 96 h in Caco-2 and HBMEC (Fig 14A–14C). The slow and gradual increase of apoptotic cells in OMV 493/89Δ*stx*_2a_-treated or OMV TA153-treated cultures between 48 h and 96 h (Fig 14A–14C) correlated with the slow and gradual decrease of G2 arrested cells during this time (Fig 12B–12D). No apoptosis was elicited by CdtV-negative TA154 OMVs (Fig 14A–14C), indicating that the apoptosis caused by TA153 and 493/89Δ*stx*_2a_ OMVs was largely mediated by CdtV.

To determine if apoptosis was the sole mode of the death of G2 arrested cells, we tested Caco-2 cells, HBMEC, and HRGEC exposed for 96 h to O157 OMVs for apoptosis and necrosis using the Cell Death Detection ELISA. OMVs from each strain induced significant apoptosis but not necrosis in each cell type (Fig 14D). The same effect was caused by CdtV-containing TA153 OMVs (but not by CdtV-lacking TA154 OMVs) and by purified Stx2a (Fig 14D). Altogether, these data demonstrate that EHEC O157 OMVs cause G2 cell cycle arrest followed by apoptosis in human intestinal epithelial and brain and renal microvascular endothelial cells. CdtV, specifically its B subunit, is the OMV component responsible for the G2 arrest, whereas both CdtV and Stx2a contribute to the apoptosis, with Stx2a being the major apoptosis inducer.

### Apoptosis caused by EHEC O157 OMVs is induced by activation of caspase-9

To identify the apoptotic pathway(s) triggered by O157 OMVs, we determined the activities of caspase-8 and caspase-9, the major initiator caspases of the extrinsic and intrinsic apoptotic pathway, respectively [50]. Caspase-9, but not caspase-8, was activated in Caco-2 cells, HBMEC and HRGEC after 48 h of exposure to O157 OMVs (Fig 14E) demonstrating that they trigger the intrinsic apoptotic pathway. Purified Stx2a (460 ng/ml) and CdtV-containing TA153 OMVs (but not CdtV-lacking TA154 OMVs) also activated caspase-9 in each cell culture (Fig 14E) confirming the involvement of both Stx2a and CdtV in the apoptosis caused by EHEC O157 OMVs.

### Gb3 is required for apoptosis caused by OMV-delivered Stx2a

To determine if the Stx receptor Gb3 is required for apoptosis induced by OMV-delivered Stx, we used the Gb3-negative DLD-1 cells and OMVs from a *cdt*V-negative EHEC O157 strain EDL933 [51] (S1 Table) to eliminate the contribution of CdtV to the apoptosis. EDL933 OMVs, which contain Stx2a and Stx1a (S34A Fig), were internalized by DLD-1 cells (S34B and S34C Fig), but did not cause apoptosis until 96 h of incubation (S34F Fig). An insight into the trafficking of the OMV-delivered Stx2a revealed that similar to Stx2a delivered to DLD-1 cells via 5791/99 OMVs (Fig 6E and 6G), the OMV EDL933-delivered toxin was trafficked to lysosomes, not reaching the endoplasmic reticulum (S34C and S34D Fig). The same was observed for OMV-delivered Stx1a (S34E Fig). These findings demonstrate that although OMV-associated Stx is internalized via OMVs by a Gb3-independent mechanism, the absence of Gb3 results in the failure of the toxin to enter the retrograde trafficking pathway and, consequently, to exert cytotoxicity.

## Discussion

The identification and characterization of a cocktail of key virulence molecules including Stx2a, CdtV, EHEC-Hly, and flagellin within EHEC O157 OMVs substantially extend previous reports [28, 29] about the presence of Stx in OMVs of EHEC O157:H7. Notably, for CdtV the OMVs represent the exclusive secretion pathway. Moreover, we explored for the first time interactions of O157 OMVs and OMV-associated toxins with pathogenetically relevant human cells including the mechanisms of cellular uptake, intracellular trafficking pathways, and biological effects leading to cell injury. By their uptake via dynamin-dependent and partially clathrin-mediated endocytosis the O157 OMVs resemble OMVs from non-O157 EHEC [10, 27] and from non-pathogenic *E*. *coli* strains [52, 53], but differ from OMVs from enterotoxigenic *E*. *coli*, *Pseudomonas aeruginosa*, and *Aggregatibacter actinomycetemcomitans*, where internalization mostly depends on caveolin and cholesterol-rich lipid rafts [23–25, 54]. Similar to other OMVs whose endocytosis involves dynamin and clathrin [10, 27, 52, 53], the O157 OMVs are internalized by target cells gradually, in a time-dependent manner (Fig 2A, 2D and 2G). Notably, the cellular uptake of O157 OMVs is apparently independent of the OMV-associated virulence factors, as we also observed previously for non-O157 OMVs containing Stx2a or EHEC-Hly [10, 27], and as was reported for Cdt-carrying OMVs from *A*. *actinomycetemcomitans* [25]. We are currently investigating OMV membrane components as candidates for OMV cell-binding ligands. Interestingly, the delivery of virulence factors into human glomerular endothelial cells via bacterial OMVs parallels the recent observation on Stx2a delivery into these cells via microvesicles derived from human blood cells [55].

**Fig 15:**
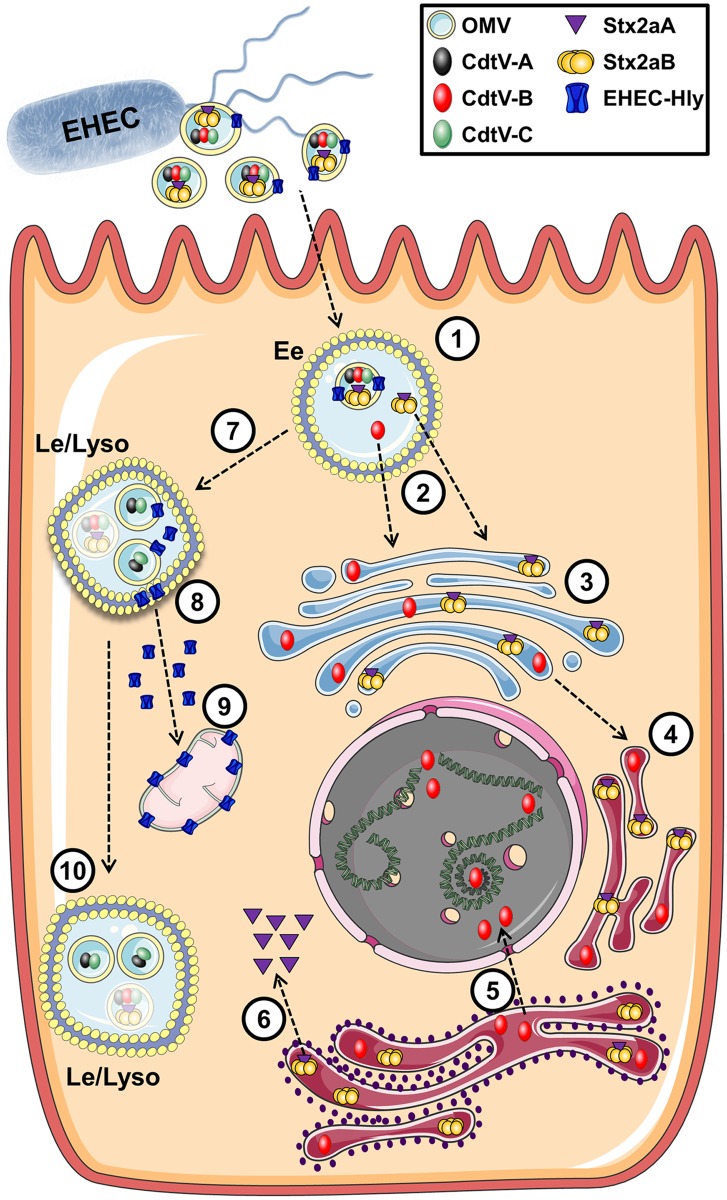
Summary of intracellular trafficking of O157 OMVs and OMV-delivered toxins. After uptake via dynamin-dependent endocytosis, O157 OMVs carrying the toxin cocktail enter the endosomal compartments of target cells (1). Stx2a holotoxin and CdtV-B subunit separate from OMVs in early endosomes (2) and are retrogradely transported to the Golgi complex (3) and the endoplasmic reticulum (4). From the endoplasmic reticulum, CdtV-B is translocated to the nucleus to target DNA (5), and Stx2a A1 catalytic fragment to the cytosol to reach ribosomes (6). CdtV-A and CdtV-C subunits and EHEC-Hly are sorted with OMVs to late endosomes/lysosomes (7). Here EHEC-Hly separates from OMVs, escapes from the lysosomes (8), and is transported to the mitochondria (9). CdtV-A and CdtV-C remain OMV-associated and are degraded with OMVs in lysosomes (10). Moreover, residual subsets of CdtV-B and Stx2a, which did not separate from OMVs in early endosomes, are sorted with OMVs to lysosomes for degradation (depicted by semi-transparent symbols).

Whereas intracellular trafficking of free, soluble Stxs and Cdts from various pathogens has been extensively studied [for review see 3, 46, 56–59], the trafficking pathways of OMV-delivered toxins are largely unknown. No such information is available for Stx and CdtV of EHEC, and only limited data exist for *A*. *actinomycetemcomitans* Cdt [25] and the CdtB-containing typhoid toxin produced by *Salmonella enterica* serovar Typhi [26]. We monitored, for the first time, in parallel the intracellular trafficking of O157 OMVs and three different OMV-delivered toxins and their subunits (for summary see Fig 15). We show that after OMV-mediated intracellular delivery, the toxins and their subunits differentially separate from OMVs to reach their cellular targets. Stx2a and CdtV-B, the DNase-like component of CdtV, mostly separate from OMVs in early endosomes and are retrogradely transported via the Golgi complex to the endoplasmic reticulum; from there the catalytic Stx2a A1 fragment is translocated to the cytosol to reach ribosomes, and CdtV-B to the nucleus to target DNA. The CdtV-A and CdtV-C subunits do not separate from OMVs and are sorted with vesicles to the late endocytic pathway, likely for degradation. EHEC-Hly releases from OMVs in late endosomes/lysosomes and subsequently targets mitochondria.

Intracellular trafficking of OMV-delivered Stx2a is in major aspects similar to that reported for free Stxs or Stx B subunit (used as a model) in toxin-sensitive cells [3, 40, 56, 57, 60–62], but several findings warrant discussion. First, in contrast to studies which identified Stx of *Shigella dysenteriae* in association with the nuclear envelope of butyric acid-sensitized epidermoid carcinoma cells [60], and Stx1 and Stx2 in nuclear fractions of human hepatoma cells [63], we did not find OMV-delivered Stx2a in the nuclei of HBMEC. This might be due to a low amount of the nuclear toxin undetectable with the methods used or cell type-specific differences in the toxin trafficking. Although Stx2a depurinated DNA and caused single-strand breaks in macrovascular endothelial cells [64], nuclear localization of the toxin in these cells has not been shown. Second, whereas free Stx enters the target cells via interaction of its B subunit with its membrane receptor Gb3 [3, 41, 42], Gb3 is not required for the cellular uptake of OMV-associated Stx2a. This is supported by the uptake of OMV-associated Stx2a, but not of free toxin, by Gb3-negative DLD-1 cells (Fig 6C and 6E). However, similar to free Stx [40, 41, 65, 66], the interaction with Gb3, in particular with its DRM-associated pool, is the prerequisite for the retrograde transport of OMV-delivered Stx2a after its liberation from OMVs, and for its cytotoxicity. This was demonstrated i) by interactions of OMV-delivered Stx2a with DRM-associated Gb3 in the Golgi complex and the endoplasmic reticulum during its retrograde transport in HBMEC (Fig 7); (ii) by converting the retrograde transport of OMV-delivered Stx2a to its lysosomal sorting by reducing the Gb3 content in HBMEC by PPMP treatment (Fig 6B); iii) by the failure of OMV-delivered Stx2a to enter the retrograde trafficking pathway, and, consequently, to cause apoptosis, in Gb3-negative DLD-1 cells (Fig 6E and 6G and S34C, S34D and S34F Fig). Third, although lysosomal sorting is a common pathway followed by free Stx and Stx B subunit in Stx-resistant cells [40, 65], the free toxin has usually not been found in lysosomes of Stx-sensitive cells [61, 67]. We show that a subset of OMV-delivered Stx2a was sorted to lysosomes in toxin-sensitive HBMEC, likely as a result of its incomplete separation from OMVs in early endosomes. Although the mechanisms that govern the separation of Stx2a from OMVs are not fully understood, our data suggest that the slight pH drop in early endosomes may facilitate this process in target cells (S17A and S17B Fig). The subsequent interaction of the OMV-liberated Stx2a with DRM-associated Gb3 is apparently the key mechanism that directs the toxin to the retrograde trafficking pathway and is thus essential for its cytotoxicity. Acidic pH in lysosomes was previously shown by our group to enable release of EHEC-Hly from OMVs and thus its transport to mitochondria [10].

Intracellular trafficking of OMV-delivered CdtV and its subunits demonstrates both similarities and differences compared to that of free Cdts or recombinant Cdt subunits from various pathogens. As with free Cdts [46, 58, 59, 68, 69], the OMV-delivered CdtV-B subunit is, after its pH-facilitated separation from OMVs in early endosomes, transported to the nucleus via the Golgi complex and the endoplasmic reticulum. The kinetics of CdtV-B appearance in the Golgi complex and late endosomes/lysosomes (Fig 8B) indicates that similar to *E*. *coli* CdtIII-B, but in contrast to *Hemophilus ducreyi* CdtB [68], CdtV-B is sorted from early endosomes directly to the Golgi complex, bypassing late endosomes. The CdtV-B signal detected in late endosomes/lysosomes, which peaked after the OMV-liberated CdtV-B had been translocated via the Golgi complex to the endoplasmic reticulum (Fig 8B), represents a subset of CdtV-B, which remained OMV-associated and was transported with OMVs (and CdtV-A/CdtV-C subunits) to lysosomes for degradation (Fig 15). This is supported by the similar kinetics of CdtV-B (Fig 8B–8D) and OMV (Fig 4B–4D) appearance in late endosomes/lysosomes. Similar to *E*. *coli* CdtIII-B [68] and *H*. *ducreyi* CdtB [68, 69], OMV-delivered CdtV-B was not found in the cytosol (Fig 8C) suggesting that it might be translocated to the nucleus directly from the endoplasmic reticulum. This is in contrast to *A*. *actinomycetemcomitans* CdtB which was observed in the cytosol using live cell imaging [70]. It is yet unclear whether or not CdtB is retranslocated from the endoplasmic reticulum to the cytosol prior to its nuclear entry since some studies demonstrated the involvement of the endoplasmic reticulum-associated degradation (ERAD) pathway in CdtB nuclear translocation and subsequent cell intoxication [71] whereas other did not [69]. The nucleus was recently identified as a target for CdtB of OMV-delivered *A*. *actinomycetemcomitans* Cdt [25] but the pathway used by this protein to gain the nuclear access was not determined.

In accordance with studies of free Cdts [68, 70], neither OMV-delivered CdtV-A nor CdtV-C entered the nucleus. However, unlike free Cdts from various pathogens whose CdtA and/or CdtC subunits are trafficked together with CdtB to the Golgi complex and the endoplasmic reticulum and support the CdtB retrograde transport [58, 59, 72, 73], OMV-delivered CdtV-C and CdtV-A were directly sorted to lysosomes where they were apparently degraded (Figs 9A–9D and 10A–10D). The lysosomal sorting of CdtV-A and CdtV-C was confirmed using OMV-delivered recombinant subunits (S28B, S28C, S28E and S28F Fig) making them less likely to be directly involved in the CdtV-B retrograde transport. However, we cannot rule out based on our data that CdtV-A and/or CdtV-C subunits might facilitate, by yet unknown mechanism(s), the CdtV-B release from the holotoxin complex and thus its liberation from OMVs in early endosomes, hereby enabling the CdtV-B entry into the retrograde trafficking pathway. In contrast to free Cdt holotoxins [11, 46, 58, 59], CdtV-A and CdtV-C are apparently not required for cellular binding of OMV-associated CdtV, as suggested by their intravesicular localization and by an efficient cellular binding and uptake of OMVs carrying recombinant CdtV-B alone (Fig 11A).

Surprisingly, but consistent with the lack of an apparent contribution of OMV-associated CdtV-A and CdtV-C to the CdtV-B trafficking, the OMV-delivered recombinant CdtV-B subunit was retrogradely transported via the Golgi complex and the endoplasmic reticulum to the nucleus (Fig 11C and 11E), and reproduced all biological effects of CdtV holotoxin including the activation of the DNA damage response, G2 arrest, and cell distension (Fig 13A–13C). Although these findings contradict the generally accepted model of cytotoxicity of free Cdts, which requires collaboration of all three Cdt subunits [74, 75], they are in agreement with the ability of *C*. *jejuni* CdtB to cause the G2 arrest and cell distension when microinjected into the cytoplasm of target cells [76], a mode of delivery which circumvents the binding roles of CdtA and CdtC, and, plausibly, also their contribution to CdtB retrograde transport. However, the requirement of both CdtB and the accessory proteins PltA and PltB for cellular intoxication by OMV-delivered typhoid toxin [26] suggests that the accessory subunits may play different roles in biological activities of different OMV-associated Cdts. The ability of the recombinant CdtV-B to produce typical biological effects of CdtV holotoxin demonstrates that similar to free Cdts [11, 46, 58, 59, 76], CdtB is the biologically active component of OMV-delivered CdtV. This was further confirmed by abolishing all the CdtV-mediated effects by *cdt*V-*B* deletion (Fig 13A–13C) as was previously also reported for OMV-associated typhoid toxin [26].

The apoptosis that followed the G2 arrest in cells exposed to O157 OMVs was largely mediated by Stx2a with the contribution of CdtV. Activation of caspase-9, but not of caspase-8, is in accordance with our previous observation that OMV-associated Stx2a triggers the intrinsic apoptotic pathway in Caco-2 cells [27], and with reports that the intrinsic pathway is the major mechanism of Cdt-induced apoptosis in intestinal epithelial and various endothelial cells [11, 77]. The mechanisms of caspase-9 activation by OMV-delivered Stx2a and CdtV remain to be determined. Although OMVs from EHEC O157:H7 can elicit HUS-like symptoms in a mouse model [78], the underlying pathogenetic mechanisms have not been studied.

In conclusion, by introducing a novel concept of OMVs as powerful tools of EHEC O157 to injure the human host, our study brings new insights into the pathogenesis of EHEC O157 infections and provides a rationale for considering O157 OMVs as vaccine candidates. The inflammatory potential of EHEC O157 OMVs and the roles of OMV-associated toxins as well as H7 flagellin and O157 LPS in this process warrant further investigations.

## Materials and methods

### Ethics statement

The strains used in this study were obtained from the Strain Collection of the Institute of Hygiene, University of Muenster, Muenster, Germany. The study was approved by the Ethical Committee of the Medical Faculty of the University of Muenster and of the Aerztekammer Westfalen-Lippe, Germany. The informed consent of the participants was not required because the data were analyzed anonymously.

### Bacterial strains used for OMV preparation

The patients´ *E*. *coli* O157 isolates and recombinant *E*. *coli* strains used in this study and their relevant genotypic and phenotypic virulence characteristics are listed in S1 Table. The cloning of the *cdt*V*-A*, *-B*, and *-C* genes and the *cdt*V*-ABC* operon into the pET23b(+) vector (Novagen) was performed using restriction-free cloning procedure [79, 80]. Briefly, the genes were PCR amplified from plasmid DNA of strain TA153 (*E*. *coli* MC1061 harboring *cdt*V*-ABC* operon from EHEC O157 strain 493/89 in SuperCos I) (S1 Table) using primer pairs listed in S5 Table. The PCR products were used to insert the genes into pET23b(+) via linear amplification [79, 80]. After treatment with D*pn*I to cleave the parental methylated plasmid, the constructs were transformed into *E*. *coli* DH5α electrocompetent cells by electroporation (MicroPulser, Bio-Rad) and the clones were selected on LB agar with ampicillin (100μg/ml). After confirmation of the inserts´ identities and correct orientation by sequencing (Seqlab, Göttingen, Germany), plasmid DNA isolated from *E*. *coli* DH5α (Zippy Plasmid Miniprep kit, Epigenetics) was electroporated into *E*. *coli* BL21(DE3) expression host (New England Biolabs) as above. The *cdt*V-*B* deletion mutant (*cdt*V*-ACΔB*) was constructed by inverse PCR using primer pair F-del-cdtB and R-del-cdtB (S5 Table) and plasmid DNA from strain BL21(DE3)/pET23b(+)*cdt*V*-ABC* (S1 Table) as a template. After confirmation of *cdt*V*-B* deletion by sequencing, the construct was electroporated into *E*. *coli* BL21(DE3).

### Antibodies

Rabbit antibodies against *E*. *coli* O157 LPS and H7 flagellin were produced [81] using EHEC O157:H7 strain EDL933 [51] and reference strain U5-41 (O1:K1:H7), respectively. Anti-Stx2a, anti-EHEC-Hly, anti-OmpA (all rabbit), and anti-CD63 (mouse) antibodies were described [10, 82, 83]. The ability of the anti-Stx2a antibody [82] to detect both Stx2a A and Stx2a B subunits in O157 OMVs was verified by comparison the immunoblot signals of OMVs with that of purified Stx2a (S35 Fig). Antibodies against CdtV-A, CdtV-B, and CdtV-C were produced by Aptum Biologics (Southampton, UK) and anti-EspPα antibody by Davids Biotechnologie (Regensburg, Germany) (all rabbit). Commercial antibodies were as follows: anti-*E*. *coli* LPS (recognizing all *E*. *coli* LPS types) (rabbit) (Biomol); anti-*E*. *coli* LPS (mouse) (Abcam); anti-*E*. *coli* O157 (mouse) (Santa Cruz); anti-phospho-Cdc2 p34 (Tyr 15), anti-phospho-Chk2 (Thr 68), anti-phospho-histone H2AX (Ser 139), anti-actin (all rabbit) (Santa Cruz); anti-phospho-ATM (Ser 1981) (mouse), anti-LAMP-1 (rabbit) (Cell Signaling Technology); anti-Rab5, anti-58K Golgi protein, anti-PDI, anti-GRP78 BiP, anti-mitochondria (MTC02) (all mouse), anti-nuclear matrix protein p84, anti-GAPDH (both rabbit) (Abcam); anti-GM130 (mouse) (Santa Cruz); anti-porin-2 (rabbit) (Novus Biological); anti-CD77/Gb3 (rat IgM) (Beckman Coulter); FITC-conjugated anti-CD77/Gb3 (mouse) (BioLegend). Alexa Fluor 488-conjugated goat anti-rabbit IgG (Molecular Probes), Alexa Fluor 488-conjugated goat anti-rat IgM (ThermoFisher Scientific), Cy3-conjugated goat anti-mouse IgG, Cy3-conjugated goat anti-rabbit IgG, and alkaline-phosphatase-conjugated goat anti-rabbit or anti-mouse IgG (all Dianova) were secondary antibodies.

### Immunoblot

Samples were separated by sodium dodecylsulfate polyacrylamide gel electrophoresis (SDS-PAGE) in a Mini-Protean TGX stain-free gel (BioRad), transferred to a polyvinylidenfluoride membrane using Trans-Blot Turbo (BioRad), blocked with 5% skimmed milk, and incubated with first antibody and alkaline-phosphatase-conjugated goat anti-rabbit or goat anti-mouse IgG. Signals were developed with NBT/BCIP substrate (Roche), visualized with Chemi Doc XRS imager (BioRad), and, if required, quantified by densitometry (Quantity One, BioRad) and expressed in arbitrary densitometric units (DU).

### Preparation of OMVs and OMV-free supernatants, detection of virulence factors, and kinetics of OMV production

OMVs from O157, TA153 and TA154 strains were isolated from 500 ml of LB broth cultures (supplemented with 100 μg/ml of ampicillin for the latter strains) as described [10] and resuspended in 1 ml of 20 mM TRIS-HCl (pH 8.0). OMV-free supernatants after ultracentrifugation were 500-fold concentrated using Vivaspin 20 concentrators, molecular weight cut-off 3,000 or 10,000 (GE Healthcare). To detect OMVs and virulence factors, OMV preparations and OMV-free supernatants (5 μg of protein/lane) were analyzed by immunoblot with antibodies against OmpA, Stx2a, CdtV-A, -B, -C, EHEC-Hly, EspPα, and H7 flagellin. Kinetics of OMV production was determined as described earlier [10, 27]. OMVs from *E*. *coli* BL21 recombinant strains were isolated from 500 ml of ampicillin-supplemented overnight LB broth cultures, which had been induced with 0.1 mM isopropyl β-D-1-thiogalactopyranoside (IPTG) (Sigma-Aldrich). The presence or absence of the respective CdtV subunits in OMV preparations resuspended in 1 ml of 20 mM TRIS-HCl (pH 8.0) was determined by immunoblot.

### OMV fractionation, dissociation assay, proteinase K (PK) assay

OMVs were fractionated by OptiPrep (Sigma-Aldrich) density gradient ultracentrifugation [10] and gradient fractions were analyzed by immunoblot with anti-OmpA, anti-Stx2a, anti-CdtV-A, -B, and -C, anti-EHEC-Hly, and anti-H7 antibodies. Dissociation assay and PK assay were performed as described [10, 25, 27] and the resulting probes were analyzed by immunoblot with antibodies against the above virulence proteins.

### Protein composition of OMVs

Pools of OmpA-containing OptiPrep gradient fractions of 5791/99, 493/89, or 493/89Δ*stx*_2a_ OMVs (5 μg of protein/lane) were separated by SDS-PAGE. OMV-associated proteins were identified in collaboration with Alphalyse (Odense, Denmark) as described [27] using in-gel tryptic digestion of total proteins from each OMV preparation followed by Q-TOF nano-LC-MS/MS. The MS/MS spectra were used for Mascot (www.matrixscience.com/) searching in the NBCI database. Protein subcellular localizations were determined with PsortB (www.psort.org/psortb/).

### Concentrations of total protein and virulence factors in OMVs

Protein concentration in OMVs was determined with Roti-Nanoquant (Carl Roth). Stx2a, EHEC-Hly, and H7 concentrations were determined as described [10, 27] using calibration curves generated from purified Stx2a [6], purified EHEC-Hly [9] or purified H7 flagellin prepared from EHEC O157:H7 strain EDL933 according to Steiner et al. [84]. To determine CdtV concentration, CdtV-B from strain 493/89 was expressed in *E*. *coli* M15[pREP4](pQE60/*cdt*V*-B*_493/89_) and purified (S1 Text). CdtV-B concentration in OMVs was determined using a calibration curve constructed from serial dilutions of the purified CdtV-B and recalculated to the concentration of CdtV holotoxin.

### Electron microscopy

Electron microscopy of ultrathin cryosections of LB agar cultures of strains 5791/99 and 493/89, or of ultrathin cryosections of OptiPrep-purified 5791/99 OMVs was performed as described [10]. Briefly, overnight LB agar cultures harvested into PBS or a pool of OptipPrep fractions 1 to 8 of 5791/99 OMVs were fixed with 2% paraformaldehyde and 0.2% glutaraldehyde and processed using the method of Tokuyasu [85]. Ultrathin (50 nm) frozen sections were cut and immunogold-stained with anti-*E*. *coli* O157 LPS antibody (culture sections), or with anti-Stx2a, anti-CdtV-A, anti-CdtV-B, anti-EHEC-Hly, and anti-H7 antibody (OMV sections) and Protein A Gold (15 nm) (Department of Cell Biology, University Medical Center, Utrecht, The Netherlands). Staining with Protein A Gold alone, without first antibodies, served as a control of specificity of the immunogold staining. For negative staining, OMVs in OptiPrep fractions of 5791/99 and 493/89 OMVs were fixed and stained with 0.5% uranyl acetate. Samples were analyzed at 80 kV on a FEI-Tecnai 12 electron microscope (FEI, Eindhoven, The Netherlands) and photographed (Ditabis imaging plates).

### Analysis of OMV size with dynamic light scattering (DLS)

The size of OMVs in OptiPrep fractions was determined with DLS [86] using a Malvern Nano Zetasizer ZS (Malvern Instruments Ltd., Worcestershire, UK) equipped with a 4 mW 633 nm He-Ne laser. Triplicate measurements of 0.5 ml OMV samples diluted 1:10 in PBS and equilibrated at 4°C for 30 s were performed with a minimum of 15 runs at a fixed 173° backscatter angle (non-invasive backscatter) using low volume disposable polymethyl methacrylate cuvettes. Data were analyzed with the Zetasizer software (Version 7.11). The average diameter (Z-average) of OMVs and the index of the particle size distribution (polydispersity index) were calculated by the method of cumulants [87].

### Cell cultures

Caco-2 and DLD-1 cells (ACC 169 and ACC 278, respectively; German collection of microorganisms and cell cultures, Braunschweig, Germany) were cultured in Quantum 286 epithelial medium (PAA), HBMEC [88] in Endothelial medium (PAA), and HRGEC (ScienCell Research Laboratories) in Endothelial cell medium with 5% of fetal bovine serum and 1% of endothelial cell growth supplement (all from ScienCell). The passages used in experiments were: Caco-2, 18 to 34; DLD-1, 8 to 15; HBMEC, 17 to 30; HRGEC, 3 to 5.

### OMV cellular uptake

To determine the kinetics of OMV cellular uptake, cells grown in 96-well plates with black frames (Greiner Diagnostics) were incubated for 15 min to 24 h at 37°C with rhodamine isothiocyanate B-R18-labeled [10] OMVs (R18-OMVs) (4 μg/ml of OMV protein). Fluorescence was measured with a fluorescence plate reader (FLUOstar OPTIMA; BMG Labtech) (excitation 560 nm, emission 590 nm) and normalized to fluorescence of R18-OMVs incubated without cells. The endocytosis inhibition assay was performed as described [10]. Briefly, cells were pretreated (1 h, 37°C) with inhibitors of endocytosis including dynasore (80 μM), chlorpromazine (15 μg/ml), filipin III (10 μg/ml), amiloride (10 mM) or cytochalasin D (1μg/ml) (all Sigma-Aldrich) or remained untreated (control). Fumonisin B1 (100 μM) was added to cells 48 h before the assay [35]. After the pretreatment, cells were incubated with R18-OMVs in the absence (control cells) or the presence of inhibitors for 4 h and analyzed for fluorescence as above. OMV uptake in the presence of each inhibitor was expressed as the percentage of OMV uptake by inhibitor-untreated cells. Tetramethylrhodamine (TMR)-conjugated transferrin (20 μg/ml), Alexa Fluor 647-conjugated cholera toxin B subunit (10 μg/ml), and TMR-conjugated dextran 10.000 (1 mg/ml) (Molecular Probes) incubated for 4 h with untreated or inhibitor-pretreated cells served as positive controls.

For CLSM, cells grown in 8-well chamber slides (ibidi) were incubated with OMVs (4 μg/ml of protein) for 30 min at 4°C (binding) followed by 30 min, 90 min, 4 h, or 24 h at 37°C (internalization). Cells exposed to OMV buffer (20 mM TRIS-HCl, pH 8.0) served as controls. After washing, fixation (3.7% paraformaldehyde), quenching (0.2 M glycine pH 7.2), permeabilization (0.25% Triton X-100) and blocking (5% bovine serum albumin) (all Sigma-Aldrich), OMVs were stained with anti-*E*. *coli* O157 LPS antibody (O157 OMVs) or anti-*E*. *coli* LPS antibody (non-O157 OMVs) and Alexa Fluor 488-conjugated goat anti-rabbit IgG. Actin was counterstained with phalloidin-tetramethyl rhodamine (TRITC) (Sigma-Aldrich) and nuclei with DRAQ5 (Cell Signaling Technology). Preparations were mounted in fluorescence mounting medium (Dako) and analyzed with a confocal laser-scanning microscope (LSM 510 META microscope, equipped with a Plan-Apochromat 63x/1.4 oil immersion objective; Carl Zeiss). To determine the effect of dynasore on OMV uptake by CLSM, cells were pretreated with 80 μM dynasore for 1 h, and without removing the inhibitor, exposed to OMVs for 4 h and processed as above. Alexa Fluor 488-conjugated transferrin (20 μg/ml) and Alexa Fluor 488-conjugated cholera toxin B subunit (10 μg/ml) (Molecular Probes) incubated with dynasore-untreated and dynasore-treated cells for 4 h served as positive controls.

### OMV-mediated intracellular delivery of virulence factors

Cell monolayers in 12-well microtiter plates were incubated for 30 min or 4 h with OMVs 5791/99 (40 μg/ml of protein containing ~3.4 μg/ml of CdtV, ~4.6 μg/ml of Stx2a, ~0.18 μg/ml of EHEC-Hly, and ~6.5 μg/ml of H7 flagellin), 493/89 (40 μg/ml of protein containing ~3.4 μg/ml of CdtV and ~4.6 μg/ml of Stx2a), 493/89Δ*stx*_2a_ (40 μg/ml of protein containing ~3.4 μg/ml of CdtV), TA153 (35 μg/ml of protein containing ~3.4 μg/ml of CdtV), TA154 (35 μg/ml of protein) or without OMVs (negative control). The cells were extensively washed and lysed with SDS-PAGE loading buffer. After heating (10 min, 99°C), cell lysates were centrifuged (16,900 x g, 15 min, 4°C) and supernatants (cytoplasmic proteins; 50 μg/lane) were analyzed by immunoblot with antibodies against OmpA, Stx2a, CdtV-A, -B, and -C, EHEC-Hly, H7 flagellin, and actin (loading control).

### Intracellular trafficking of OMVs and OMV-delivered toxins/toxin subunits in HBMEC detected by CLSM

To analyze intracellular trafficking of O157 OMVs and OMV-delivered virulence factors, HBMEC grown in 8-well ibidi slides were preincubated with 5791/99 OMVs (4 μg/ml of protein containing ~340 ng/ml of CdtV, ~460 ng/ml of Stx2a, and ~18 ng/ml of EHEC-Hly) for 30 min at 4°C (OMV cell binding), and without washing, postincubated for 15 min, 30 min, 90 min, 4 h, and 20 h at 37°C (OMV internalization). Cells were then washed, fixed and quenched as above, and permeabilized/blocked with PBS containing 5% goat serum, 1% bovine serum albumin and 1 mg/ml saponin (Sigma-Aldrich). To monitor colocalizations of OMV-delivered toxins/toxin subunits with OMVs, OMVs were stained with mouse anti-*E*. *coli* O157 antibody and Cy3-conjugated goat anti-mouse IgG, and Stx2a, CdtV-A, -B, -C, and EHEC-Hly with the respective rabbit antibodies and Alexa Fluor 488-conjugated goat anti-rabbit IgG. To monitor intracellular trafficking of OMVs and OMV-delivered toxins/toxin subunits, OMVs were stained with rabbit anti-*E*. *coli* O157 LPS antibody, and the toxins/toxin subunits with the respective toxin-specific antibodies, followed by Alexa Fluor 488-conjugated goat anti-rabbit IgG. Early endosomes, late endosomes/lysosomes, Golgi complex, endoplasmic reticulum, and mitochondria were stained with mouse anti-Rab5, anti-CD63, anti-K58 Golgi protein, anti-PDI, and anti-mitochondria (MTC02) antibodies, respectively, and Cy3-conjugated goat anti-mouse IgG. Nuclei were stained with DRAQ5. Preparations were analyzed with a confocal laser-scanning microscope (LSM 510 META microscope equipped with a Plan-Apochromat 63x/1.4 oil immersion objective). Single channels and the merged images (consisting of one optical section of a z-series with a pinhole of 1 airy unit) are shown. To quantify colocalizations between signals of interest, digital colocalization images were imported into BioImageXD6 [89], signals were manually thresholded, and the percentage of colocalized signals was calculated using the BioImageXD6 colocalization tool. The specificity of the OMV/virulence factors-related signals observed in HBMEC treated with 5791/99 OMVs was verified i) by incubating the cells for 20 h with OMV buffer instead of OMVs and staining them for OMVs and OMV-delivered virulence factors as above; and ii) by incubating the cells with 5791/99 OMVs for 20 h and staining them only with secondary antibodies in the absence of primary antibodies. In no case, either OMVs or any of the virulence proteins were detected (S36A and S36B Fig).

To analyze intracellular trafficking of OMV-delivered recombinant CdtV subunits, HBMEC were preincubated for 30 min at 4°C, and, without washing, postincubated (90 min to 20 h, 37°C) with OMVs (4 μg/ml of protein) from strains BL21(*cdt*V-*B*) (containing ~140 ng/ml of CdtV-B), BL21(*cdt*V-*A*), or BL21(*cdt*V-*C*); OMVs from BL21(*cdt*V-*ABC*) (containing ~142 ng/ml of CdtV-B and ~370 ng/ml of CdtV), BL21(*cdt*V-*ACΔB*) or from the vector control BL21(pET23) served as controls. To monitor colocalizations of CdtV subunits with OMVs, OMVs were stained with mouse anti-*E*. *coli* LPS antibody and Cy3-conjugated goat anti-mouse IgG, and CdtV-A, -B, and -C with the respective rabbit antibodies and Alexa Fluor 488-conjugated goat anti-rabbit IgG. To monitor intracellular trafficking of OMVs and OMV-delivered CdtV subunits, OMVs were stained with rabbit anti-*E*. *coli* LPS antibody, and CdtV-A, -B, and -C with their respective antibodies, followed by Alexa Fluor 488-conjugated goat anti-rabbit IgG. The Golgi complex, endoplasmic reticulum, and late endosomes/lysosomes were stained with primary antibodies described above and Cy3-conjugated goat anti-mouse IgG. Colocalizations were quantified as above.

### Intracellular trafficking of O157 OMVs and OMV-delivered and free Stx2a in PPMP-treated HBMEC, Triton X-100-extracted HBMEC, and DLD-1 cells detected by CLSM

PPMP treatment was performed by growing HBMEC for 6 days in Endothelial medium containing 5 μM PPMP (Sigma-Aldrich) as described previously for HeLa cells [41]. PPMP-treated (and control PPMP-untreated) HBMEC or DLD-1 cells were incubated with 5791/99 OMVs (for concentrations of the total protein and Stx2a see above) or Stx2a-negative 493/89Δ*stx*_2a_ OMVs (4 μg/ml of protein) for 30 min at 4°C followed by postincubation at 37°C for 20 h (HBMEC) or 4 h (DLD-1). Free purified Stx2a [6] (~460 ng/ml) was added to PPMP-treated and DLD-1 cells directly at 37°C to allow its endocytosis [41]. Cells were fixed, quenched, permeabilized and stained for OMVs, Stx2a and intracellular compartments (endoplasmic reticulum, lysosomes) as described above. In the Triton X-100 extraction experiments, HBMEC were cooled on ice (5 min) at the end of incubation with OMVs or free Stx2a, washed with ice-cold PBS, and incubated for 1 min on ice with 1% Triton X-100/PIPES buffer (80 mM PIPES, pH 6.8, 5 mM EGTA, 1 mM MgCl_2_) [40] before fixation. Gb3 was stained with rat anti-CD77/Gb3 IgM and Alexa Fluor 488-conjugated goat anti-rat IgM, Stx2a with rabbit anti-Stx2a antibody and Cy3-conjugated or Alexa Fluor 488-conjugated goat anti-rabbit IgG, and the Golgi complex and the endoplasmic reticulum with mouse anti-GM130 and anti-Bip antibody, respectively, and Cy3-conjugated goat anti-mouse IgG. Preparations were analyzed by CLSM as above. To determine the trafficking of Stx2a delivered by EDL933 OMVs in DLD-1 cells, cells were exposed to the OMVs (4 μg/ml of protein containing ~430 ng/ml of Stx2a and ~ 210 ng/ml of Stx1a) for 30 min at 4°C followed by 4 h at 37°C, and then fixed, quenched, permeabilized and stained for OMVs, Stx2a and intracellular compartments (endoplasmic reticulum, lysosomes) as described above.

### Detection and quantification of Gb3

Gb3 in HBMEC, PPMP-treated HBMEC and DLD-1 cells was visualized by CLSM of permeabilized cells using rat anti-CD77/Gb3 IgM antibody and Alexa Fluor 488-conjugated goat anti-rat IgM. Quantification of Gb3 was performed by FACS analysis. Cells (1 x 10^7^) were fixed with 3.7% paraformaldehyde, permeabilized with 0.2% Triton X-100, and stained with FITC-conjugated anti-CD77/Gb3 antibody (1 h on ice). After washing, the fluorescence of at least 10^4^ cells was acquired with FACSCalibur (Becton Dickinson) using green channel. The data were analyzed with CellQuest Pro (Becton Dickinson) and expressed as geometric means of fluorescence from 10,000 events. Unstained cells served as controls.

### Effect of pH and bafilomycin A1 on the separation of Stx2a and CdtV-B from OMVs

To investigate the effect of pH, 5791/99 OMVs (~10 μg of OMV protein) were incubated (1 h, 37°C) in 20 mM TRIS-HCl buffer with pH ranging from 8.0 to 5.0. The samples were then ultracentrifuged (235,000 x g, 2 h, 4°C), and the pellets (OMVs) and supernatants (OMV-released proteins) were analyzed for Stx2a and CdtV-B by immunoblot. Signals were quantified densitometrically and the percentage of each protein present in the pellet and supernatant at each particular pH was calculated from the total signal. To determine the effect of bafilomycin A1, HBMEC were pretreated with 100 nM bafilomycin A1 (Sigma-Aldrich) for 1 h at 37°C, and without removing the inhibitor, exposed (30 min at 4°C followed by 4 h at 37°C) to OMVs 5791/99. The presence of Stx2a and CdtV-B in the endoplasmic reticulum was analyzed by CLSM as described above.

### Isolation and immunoblot analyses of subcellular fractions of HBMEC, PPMP-treated HBMEC and DLD-1 cells

Confluent HBMEC monolayers (~8 x 10^6^ cells) were incubated with 5791/99 OMVs (4 μg/ml of protein containing ~340 ng/ml of CdtV, ~460 ng/ml of Stx2a, and ~18 ng/ml of EHEC-Hly) for 30 min at 4°C, and postincubated for 30 min to 72 h at 37°C. Untreated cells and cells treated for 72 h with OMV buffer instead of OMVs served as controls. Lysosomal and mitochondrial fractions were isolated using the Lysosome Enrichment Kit for Tissue and Cultured Cells and the Mitochondria Isolation Kit for Cultured Cells (both Thermo Scientific), respectively, as described previously [10]. Total (smooth and rough) endoplasmic reticulum fraction was prepared with the Endoplasmic Reticulum Enrichment Kit (Novus Biological) according to the manufacturer´s instructions. Nuclear fraction, cytosolic fraction, and whole cell extracts were prepared using the Nuclear Extraction Kit (Active Motif) following the manufacturer´s protocols. Protein concentrations in isolated fractions and whole cell extracts were determined with Roti-Nanoquant. To verify the quality of the fractions and exclude their cross-reactivity, samples (~50 μg of protein/lane) were separated by SDS-PAGE and analyzed by immunoblot with antibodies against compartment-specific marker proteins including LAMP-1 (lysosomes), porin-2 (mitochondria), PDI (endoplasmic reticulum), nuclear matrix protein p84 (nucleus), and GAPDH (cytosol). The presence of OMVs and virulence factors (Stx2a, CdtV-A, -B, -C, EHEC-Hly) within the fractions was analyzed by immunoblot with the respective antibodies.

To determine the presence of recombinant CdtV-A, -B and -C in subcellular fractions, HBMEC were exposed to OMVs (4 μg/ml of OMV protein) from strains BL21(*cdt*V-*A*), BL21(*cdt*V-*B*) (containing ~140 ng/ml of CdtV-B), BL21(*cdt*V-*C*), BL21(*cdt*V-*ABC*) (containing ~142 ng/ml of CdtV-B and ~370 ng/ml of CdtV) (positive control), BL21(*cdt*V-*ACΔB*), or from the vector control BL21(pET23) (negative control) for 30 min at 4°C followed by 90 min to 20 h at 37°C. Endoplasmic reticulum, nuclear and lysosomal fractions isolated as above were analyzed for the respective CdtV subunits by immunoblot.

To analyze the presence of Stx2a in subcellular fractions of PPMP-treated HBMEC or DLD-1 cells, cells were incubated with 5791/99 OMVs (concentrations of the total protein and Stx2a as above), Stx2a-negative 493/89Δ*stx*_2a_ OMVs (4 μg/ml of protein), EDL933 OMVs (4 μg/ml of protein containing ~430 ng/ml of Stx2a and ~ 210 ng/ml of Stx1a) or free Stx2a (~460 ng/ml) as described for CLSM. Endoplasmic reticulum and lysosomal fractions were isolated as above and analyzed for Stx2a by immunoblot.

### CdtV-mediated DNA damage signaling, cell cycle arrest and cell distension

To detect the DNA damage signaling, Caco-2 cells, HBMEC and HRGEC were incubated for 20 h with OMVs 5791/99 (4 μg/ml of protein containing ~340 ng/ml of CdtV, ~460 ng/ml of Stx2a, and ~18 ng/ml of EHEC-Hly), 493/89 (4 μg/ml of protein containing ~340 ng/ml of CdtV and ~460 ng/ml of Stx2a), 493/89Δ*stx*_2a_ (4 μg/ml of protein containing ~340 ng/ml of CdtV), TA153 (3.5 μg/ml of protein containing ~340 ng/ml of CdtV) or TA154 (3.5 μg/ml of protein) or remained untreated. Cells were washed and lysed with SDS-PAGE loading buffer. Cell lysates were heated (10 min, 99°C), centrifuged (16,900 x g, 15 min, 4°C), and the supernatants (cytoplasmic proteins; 50 μg/lane) were analyzed by immunoblot with anti-γ-H2AX, anti-phospho-ATM, anti-phospho-Chk2, anti-phospho-cdc2 or anti-actin (loading control) antibodies. To detect the DNA damage signaling induced by recombinant CdtV-B, HBMEC were incubated for 20 h with OMVs (4 μg/ml of protein) from strains BL21(*cdt*V-*B*) (containing ~140 ng/ml of CdtV-B), BL21(*cdt*V-*ABC*) (containing ~142 ng/ml of CdtV-B and ~370 ng/ml of CdtV) (positive control), BL21(*cdt*V-*ACΔB*), or the vector control BL21(pET23) (negative controls); cell lysates prepared as above were analyzed for γ-H2AX and p-cdc2 by immunoblot.

The cell cycle was analyzed as described previously [8] using cells exposed to O157, TA153 or TA154 OMVs (concentrations as above), OMV buffer or left untreated for 24 h to 96 h. The effect of OMVs from the recombinant strains BL21(*cdt*V-*B*), BL21(*cdt*V-*ACΔB*), BL21(*cdt*V-*ABC*), and the vector control BL21(pET23) was determined after 48 h of exposure. After staining of nuclei with propidium iodide (Sigma-Aldrich), G2 arrested cells (4n DNA content) were quantified by flow cytometry (FACSCalibur, Becton Dickinson) (emission 570 nm, FL-2 channel). Data from 10^4^ nuclei were analyzed by CellQuest software (Becton Dickinson). To determine dose-dependence of the G2 arrest, cells were exposed to O157 or TA153 OMVs containing CdtV in doses ranging from 340 ng/ml to 5.3125 ng/ml for 24 h (HRGEC) or 48 h (Caco-2, HBMEC) and analyzed for DNA content as above.

In the distension assay, freshly seeded Caco-2 cells, HBMEC or HRGEC were exposed to O157, TA153 or TA154 OMVs (concentrations as above) for 72 h or remained untreated. OMVs from the recombinant strains BL21(*cdt*V-*B*), BL21(*cdt*V-*ACΔB*), BL21(*cdt*V-*ABC*), and BL21(pET23) were incubated for 72 h with HBMEC. Morphology was examined in native cells (HRGEC) or cells fixed with 70% ethanol and stained with 10% Giemsa using Axio Imager A1 microscope (Carl Zeiss).

### Quantification of apoptosis, caspase activation, and Cell Death Detection ELISA

To measure apoptosis, cells were exposed for 24 h to 96 h to 5791/99, 493/89, 493/89Δ*stx*_2a_, TA153, or TA154 OMVs (for concentrations of protein and virulence factors see above), purified Stx2a [6] (460 ng/ml), 1 μM staurosporine (Sigma) (positive control), OMV buffer or left untreated (negative controls). Apoptosis was quantified by flow cytometric detection (FACSCalibur) of hypodiploid nuclei after propidium iodide staining as described [8, 90] and the data from 10^4^ nuclei were analyzed by CellQuest software.

Caspase-9 and caspase-8 activities were assayed in cells exposed to the above OMVs, OMV buffer or purified Stx2a for 48 h or left untreated using Caspase Colorimetric Substrate Kit I (Biozol Diagnostica) [10]. The color intensity, which is proportional to the level of caspase enzymatic activity, was measured spectrophotometrically and the caspase activities in sample-treated cells were expressed as a fold-increase of their activities in untreated cells. Inhibitor of caspase-9 (z-LEHD-fmk) (R & D Systems) (100 μM) was added to cells 30 min prior to the samples.

The Cell Death Detection ELISA^PLUS^ (Roche) was performed as described [6] using cells incubated for 96 h with O157, TA153 or TA154 OMVs, purified Stx2a (460 ng/ml), OMV buffer or left untreated. Enrichment factors of apoptosis and necrosis were calculated by dividing OD_405_ absorbance values of sample-treated cells with those of untreated cells. To determine apoptotic potential of EDL933 OMVs in DLD-1 cells, the Cell Death Detection ELISA was performed as above after 96 h incubation of the cells with EDL933 OMVs (4 μg/ml of protein containing ~430 ng/ml of Stx2a and ~ 210 ng/ml of Stx1a), free Stx2a (460 ng/ml), staurosporine (1 μM) (positive control) and OMV buffer (negative control).

### Statistical analysis

Data were analyzed with one-way ANOVA (analysis of variance), two-tailed unpaired Student´s *t*-test or paired Student´s *t*-test. *p* values < 0.05 were considered significant.

### Accession numbers for genes and proteins mentioned in the text

GenBank NC_002695.1 (1266965..1267924) Shiga toxin 2 subunit A gene (ECs1205)

GenBank NC_002695.1 (1267936..1268205) Shiga toxin 2 subunit B gene (ECs1206)

UniProtKB/Swiss-Prot Q7DI68 Shiga toxin 2 subunit A *Escherichia coli* O157:H7

UniProtKB/Swiss-Prot A7UQX3 Shiga toxin 2 subunit B *Escherichia coli* O157:H7

GenBank AJ508930.1 *Escherichia coli cdtA* gene, *cdtB* gene and *cdtC* gene

UniProtKB/Swiss-Prot Q8GJ13 Cytolethal distending toxin-V A subunit

UniProtKB/Swiss-Prot O32586 Cytolethal distending toxin-V B subunit

UniProtKB/Swiss-Prot Q8GJ12 Cytolethal distending toxin-V C subunit

GenBank X79839.1 EHEC-*hlyA* gene

UniProtKB/Swiss-Prot Q47262 Hemolysin *Escherichia coli* (EHEC-Hly)

GenBank NC_002695.1 (2624379..2626136) *fliC* gene *Escherichia coli* O157:H7 (ECs2662)

UniProtKB/SwissProt Q7AD06 Flagellin *Escherichia coli* O157:H7

GenBank NC_002695.1 (1148484..1149524) *ompA* gene (ECs1041)

UniProtKB/SwissProt P0A911 Outer membrane protein A *Escherichia coli* O157:H7

GenBank X97542.1 (2571..6473) *espP* gene

UniProtKB/SwissProt Q7BSW5 Serine protease EspP *Escherichia coli* O157:H7

## Supporting information

S1 Fig Kinetics of OMV production by *E*. *coli* O157 strains and protein composition of OMVs. (A, B, C) Strains were grown in LB broth, OMVs were isolated at indicated times, subjected to immunoblot with anti-OmpA antibody, and quantified by densitometry of OmpA signals (expressed in arbitrary densitometric units; DU). OMV-free supernatants served as controls. Bacterial growth was monitored by measuring OD_600_. Data are means ± standard deviations from three independent experiments. (D) Distribution of OMV-associated proteins identified with nano-LC-MS/MS according to their subcellular localization determined by PsortB prediction tool.
(TIF)

S2 Fig Virulence factors are tightly associated with O157 OMVs as evidenced by dissociation assay. OptiPrep-purified OMVs from strains 5791/99, 493/89, and 493/89Δ*stx*_2a_ were incubated in HEPES buffer alone (control), or in HEPES buffer with the indicated chemicals. After ultracentrifugation, pellets (P; containing OMVs) and supernatants (S; containing proteins released from OMVs) were analyzed by immunoblot with antibodies against the virulence factors or their subunits.
(TIF)

S3 Fig Electron microscopic analysis of OptiPrep-fractionated OMVs from EHEC O157 strains 5791/99 and 493/89. OptiPrep gradient fractions of 5791/99 OMVs (F1 –F8) and 493/89 OMVs (F1 –F9) were negatively stained with 0.5% uranyl acetate and analyzed with a FEI-Tecnai 12 electron microscope. Scale bars are 200 nm.
(TIF)

S4 Fig Size of OptiPrep-fractionated OMVs from EHEC O157 strains 5791/99 (A) and 493/89 (B) determined by dynamic light scattering (DLS). The x-axis displays the OMV size distribution in each fraction and the y-axis the scattered light intensity of the OMVs. In (C) and (D) the average diameter (Z-average) of OMVs and the index of the particle size distribution (polydispersity index; PDI) in each fraction is shown (d.nm, diameter in nm). The Z-averages and PDIs were calculated by the method of cumulants using the Zetasizer software.
(TIF)

S5 Fig OMV uptake by Caco-2 and HBMEC and presence of CdtV in TA153 OMVs. (A, C) CLSM visualization of binding (panels 0 min) and uptake of O157 (A) and TA153 and TA154 OMVs (C) by Caco-2 cells and HBMEC after 90 min, 4 h and 24 h of incubation. OMVs (green) were detected with anti-*E*. *coli* O157 LPS (A) or anti-*E*. *coli* LPS (C) antibody and Alexa Fluor 488-conjugated goat anti-rabbit IgG, actin (red) with phalloidin-TRITC and nuclei (blue) with DRAQ5. Confocal Z-stack projections are included at upper/right sides. Crosshairs show the position of the xy and yz planes. Scale bars are 10 μm. (D) CLSM of control cells incubated with OMV buffer instead of OMVs for 24 h and stained and processed as described in A and C. (B) Distribution of CdtV-A, CdtV-B, and CdtV-C proteins in OMVs and OMV-free supernatants of strains TA153 (containing the *cdt*V*-ABC* operon from strain 493/89 in SuperCos I) and TA154 (vector control) determined by immunoblot with antibodies against OmpA (an OMV marker) and the respective CdtV subunits.
(TIF)

S6 Fig Activities of inhibitors of endocytosis and effect of dynasore on cellular uptake of OMVs and controls demonstrated by CLSM. (A, B) Activities of inhibitors of endocytosis used in this study against markers of different endocytosis pathways including clathrin-mediated endocytosis (tetramethylrhodamine-conjugated transferrin; Tf-TMR), lipid rafts/caveolae-mediated endocytosis (Alexa Fluor 647-conjugated cholera toxin B subunit; CT-B-AF647), and macropinocytosis (TMR-conjugated Dextran 10.000; Dextran-TMR). Caco-2 cells (A) and HBMEC (B) either untreated (no inhibitor) or pretreated with the indicated inhibitors were incubated with Tf-TMR, CT-B-AF647 or Dextran-TMR for 4 h and fluorescence was measured with FLUOstar OPTIMA fluorometer. The uptake of each marker in the presence of inhibitors was expressed as the percentage of its uptake by inhibitor-untreated cells (100%). Data are means ± standard deviations from three independent experiments. ** *p* < 0.01, and *** *p* < 0.001 compared to inhibitor-untreated cells (one-way ANOVA). (C-F) Effect of dynasore on the uptake of OMVs (C, E) and control endocytosis markers (D, F) by Caco-2 cells (C, D) and HBMEC (E, F) visualized by CLSM after 4 h of incubation of cells with the indicated samples. Panels marked Dynasore show dynasore-pretreated cells. Green, OMVs (C, E) or Alexa Fluor 488-conjugated transferrin (Tf-AF488) or Alexa Fluor 488-conjugated cholera toxin B subunit (CT-B-AF488) (D, F); red, actin; blue, nuclei. Confocal Z-stack projections are included at upper/right sides. Crosshairs show the position of the xy and yz planes. Scale bars are 10 μm. For evaluation of the effect of dynasore on OMV uptake, compare the OMV amounts in dynasore-treated cells (C, E, panels Dynasore) with those of the respective OMVs in dynasore untreated cells (S5A and S5C Fig, panels 4 h).
(TIF)

S7 Fig EHEC O157 virulence factors are internalized via OMVs. Immunoblot detection of OMVs (anti-OmpA antibody) and OMV-associated virulence factors in lysates of Caco-2 cells, HBMEC, and HRGEC which were incubated with O157 OMVs (A, B) or control CdtV-containing (TA153) or CdtV-lacking (TA154) OMVs (C) for 30 min and 4 h. Untreated cells (no OMV) were negative controls. Actin served as a loading control. (The CdtV-C signal in lane no OMV in the HRGEC lysate in panel A is an artifact resulting from contamination by sample from the previous lane).
(TIF)

S8 Fig Quantification and statistical analysis of colocalizations of CLSM signals in immunofluorescence images shown in Figs 3–5 and 8–10, and S31 Fig. Graphical presentations of CLSM colocalizations between (A) 5791/99 OMVs and OMV-delivered virulence factors, (B) 5791/99 OMVs and subcellular compartments, and (C-G) the indicated OMV-delivered virulence proteins and subcellular compartments during time. The subcellular compartments investigated and their markers were: early endosomes (Rab5), late endosomes/lysosomes (CD63), Golgi complex (K58), endoplasmic reticulum (PDI), mitochondria (MTC02), and nucleus (DNA). The percentages of colocalizations between signals of interest were determined with the BioImageXD6 tool. Data are shown as means ± standards deviations from measurements of at least five (for CdtV-A/CdtV-C of at least three) different samples. *significantly increased or decreased (*p* < 0.05; two-tailed unpaired Student’s *t*-test) compared to the previous time interval or between the spanned time intervals.
(TIF)

S9 Fig Enlarged single fluorescence channels of CLSM images shown in Fig 3A (time points 0 min to 30 min). Scale bars are 10 μm.
(TIF)

S10 Fig Enlarged single fluorescence channels of CLSM images shown in Fig 3A (time points 90 min to 20 h). Scale bars are 10 μm.
(TIF)

S11 Fig Immunoblot analyses of isolated HBMEC subcellular fractions with antibodies against compartment-specific marker proteins. (A) Lysosomal (lyso), mitochondrial (mito), endoplasmic reticulum (ER), nuclear (nucl), and cytosolic (cyto) fractions were isolated from HBMEC which had been incubated for the indicated times with 5791/99 OMVs or for 72 h with OMV buffer (20 mM TRIS-HCl, pH 8.0) or left untreated (no OMV). The fractions were analyzed by immunoblot with antibodies against the indicated compartment-specific marker proteins. 5791/99 OMVs without cells (OMV ctrl) served as a negative control. (B) Whole cell lysates (WCL) (positive control) and subcellular fractions prepared from HBMEC exposed to 5791/99 OMVs for 20 h were analyzed by immunoblot with antibodies against homologous and heterologous compartment-specific marker proteins.
(TIF)

S12 Fig Enlarged single fluorescence channels of CLSM images shown in Fig 4A (time points 30 min and 90 min). Scale bars are 10 μm. Ee, early endosomes; Le/Lyso, late endosomes/lysosomes; ER, endoplasmic reticulum; Mito, mitochondria.
(TIF)

S13 Fig Enlarged single fluorescence channels of CLSM images shown in Fig 4A (time points 4 h and 20 h). Scale bars are 10 μm. Ee, early endosomes; Le/Lyso, late endosomes/lysosomes; ER, endoplasmic reticulum; Mito, mitochondria.
(TIF)

S14 Fig Intracellular trafficking of Stx2a B subunit detected by immunoblot. (A) Immunoblot detection of Stx2a B subunit in isolated subcellular fractions of HBMEC which were incubated for the times indicated with 5791/99 OMVs or for 72 h without OMVs or with OMV buffer (20 mM TRIS-HCl) (negative controls); OMVs without cells (OMV ctrl) served as a positive control. (B) Densitometric quantification of Stx2aB signals in endoplasmic reticulum and lysosomal fractions shown in A. Abbreviations used: ER, endoplasmic reticulum; Cyto, cytosol; Lyso, lysosomes; Mito, mitochondria; Nucl, nucleus; DU, densitometric unit.
(TIF)

S15 Fig Enlarged single fluorescence channels of CLSM images shown in Fig 5A (time points 30 min and 90 min). Scale bars are 10 μm. Ee, early endosomes; ER, endoplasmic reticulum; Le/Lyso, late endosomes/lysosomes; Mito, mitochondria.
(TIF)

S16 Fig Enlarged single fluorescence channels of CLSM images shown in Fig 5A (time points 4 h and 20 h). Scale bars are 10 μm. Ee, early endosomes; ER, endoplasmic reticulum; Le/Lyso, late endosomes/lysosomes; Mito, mitochondria.
(TIF)

S17 Fig pH facilitates separation of Stx2a and CdtV-B from OMVs. (A) 5791/99 OMVs were incubated in TRIS-HCl with the indicated pH range for 1 h and then ultracentrifuged. The pellets (P) containing OMV-associated proteins and supernatants (S) containing proteins that separated from OMVs were analyzed by immunoblot with antibodies against Stx2a or CdtV-B. The Stx2a and CdtV-B signals were quantified densitometrically and the percentage of each protein present in the P and S fraction at each particular pH was calculated from the total signal. (B) HBMEC were not (no BafA1) or were pretreated (+ BafA1) for 1 h with 100 nM bafilomycin A1 (BafA1), incubated for 4 h with 5791/99 OMVs, and analyzed for the presence of Stx2a or CdtV-B in the endoplasmic reticulum by CLSM. The right panels show the indicated single fluorescence channels, and the left panels the merged images (green, Stx2a or CdtV-B; red, PDI; blue, nuclei; yellow, colocalized green and red signals). Scale bars are 10 μm. Note the differences between the intensities of Stx2a and CdtV-B signals, respectively, in BafA1-untreated and BafA1-treated cells.
(TIF)

S18 Fig Gb3 content in HBMEC, PPMP-treated HBMEC, and DLD-1 cells, and graphs and negative controls to CLSM data shown in Figs 6 and 7. (A, B) Graphical presentations of FACS analyses of Gb3 content in PPMP-untreated (PPMP-) and PPMP-treated (PPMP+) HBMEC (A) and in DLD-1 cells (B) stained with anti-CD77/Gb3-FITC antibody or unstained (control). Geometric mean fluorescence ± standard deviations from three independent experiments are shown. ***p < 0.001 (paired Student´s *t*-test) for Gb3 content in PPMP-treated compared to PPMP-untreated HBMEC. (C, D) Visualization of Gb3 in PPMP-untreated and PPMP-treated HBMEC (C) and in DLD-1 cells (D) by CLSM. Green, Gb3; blue, nuclei. (F, G) Negative controls to Fig 6B (F) and Fig 6E (G). Note the absence of Stx2a signals (green) in cells exposed to OMVs from Stx2a-negative strain 493/89Δ*stx*_2a_ for 20 h (F) or left untreated (no OMV) (G). (I) Negative controls to Fig 7D. Note the absence of Stx2a signals (green) in cells which had been exposed to OMVs from Stx2a-negative strain 493/89Δ*stx*_2a_ for 90 min or 4 h or left untreated (no OMV) before they were processed for CLSM directly (no Triton) or after 1 min extraction with Triton X-100-containing buffer (+ Triton). In C, D, F, G and I, the indicated single fluorescence channels are shown in the right panels and the merged images in the left panels. Scale bars are 10 μm. (E, H) Graphical presentations of CLSM data shown in Fig 6B. Means ± standard deviations of colocalizations from three different samples are shown. ***p < 0.001 (paired Student´s *t*-test) for colocalization rates in PPMP-treated compared to PPMP-untreated HBMEC.
(TIF)

S19 Fig Enlarged single fluorescence channels of CLSM images shown in Fig 6. (A) HBMEC (Fig 6B), and (B) DLD-1 cells (Fig 6E). Scale bars are 10 μm. No PPMP, PPMP-untreated HBMEC; +PPMP, PPMP-treated HBMEC. ER, endoplasmic reticulum; Le-Lyso, late endosomes/lysosomes.
(TIF)

S20 Fig Enlarged single fluorescence channels of CLSM images shown in Fig 7. (A) Images shown in Fig 7A, 7B and 7C. (B) Images shown in Fig 7D. HBMEC were processed for CLSM either untreated (no Triton) or after pretreatment with Triton X-100 (+Triton). Scale bars are 10 μm. ER, endoplasmic reticulum.
(TIF)

S21 Fig Enlarged single fluorescence channels of CLSM images shown in Fig 8A (time points 30 min and 90 min). Scale bars are 10 μm. Ee, early endosomes; ER, endoplasmic reticulum; Le/Lyso, late endosomes/lysosomes; Mito, mitochondria.
(TIF)

S22 Fig Enlarged single fluorescence channels of CLSM images shown in Fig 8A (time points 4 h and 20 h). Scale bars are 10 μm. Ee, early endosomes; ER, endoplasmic reticulum; Le/Lyso, late endosomes/lysosomes; Mito, mitochondria.
(TIF)

S23 Fig Enlarged single fluorescence channels of CLSM images shown in Fig 9A (time points 30 min and 90 min). Scale bars are 10 μm. Ee, early endosomes; Le/Lyso, late endosomes/lysosomes; ER, endoplasmic reticulum; Mito, mitochondria.
(TIF)

S24 Fig Enlarged single fluorescence channels of CLSM images shown in Fig 9A (time points 4 h and 20 h). Scale bars are 10 μm. Ee, early endosomes; Le/Lyso, late endosomes/lysosomes; ER, endoplasmic reticulum; Mito, mitochondria.
(TIF)

S25 Fig Enlarged single fluorescence channels of CLSM images shown in Fig 10A (time points 30 min and 90 min). Scale bars are 10 μm. Ee, early endosomes; Le/Lyso, late endosomes/lysosomes; ER, endoplasmic reticulum; Mito, mitochondria.
(TIF)

S26 Fig Enlarged single fluorescence channels of CLSM images shown in Fig 10A (time points 4 h and 20 h). Scale bars are 10 μm. Ee, early endosomes; Le/Lyso, late endosomes/lysosomes; ER, endoplasmic reticulum; Mito, mitochondria.
(TIF)

S27 Fig Expression and localization of CdtV-A, CdtV-B, and CdtV-C in OMVs from *E*. *coli* BL21 recombinant strains harboring the single subunit genes, the *cdt*V operon, and *cdt*V*-B* deletion. (A) Immunoblot analyses of OMVs from the indicated strains with anti-CdtV-A, anti-CdtV-B, and anti-CdtV-C antibodies. OMVs from BL21(pET23) (vector control) served as a negative control. OmpA is an OMV marker. (B) Intravesicular localization of the recombinant CdtV subunit proteins demonstrated by the proteinase K (PK) assay. PK-untreated (PK-) or PK-treated (PK+) OMVs from the indicated strains, either intact (EDTA-) or lysed with 0.1 M EDTA (EDTA+), were separated by SDS-PAGE and analyzed by immunoblot with the indicated antibodies. BL21(*cdt*V-*ABC*) OMVs carrying CdtV holotoxin were used as a control.
(TIF)

S28 Fig Cellular uptake and trafficking of OMV-associated recombinant CdtV-A and CdtV-C, and CLSM controls for trafficking of recombinant CdtV-B. HBMEC were preincubated (30 min, 4°C) with OMVs from the indicated recombinant strains and postincubated at 37°C for 90 min to 20 h. (A) OMV uptake was determined by CLSM after 4 h. Green, OMVs; red, actin; blue, nuclei. Confocal Z-stack projections are included at upper/right sides. Crosshairs show the position of the xy and yz planes. Scale bars are 10 μm. (B, C) Colocalization of OMVs and OMV-delivered CdtV-A and CdtV-C expressed either singly (OMVs BL21(*cdt*V-*A*) and BL21(*cdt*V-*C*), respectively) or together (OMVs BL21(*cdt*V-*ACΔB*)) with the endoplasmic reticulum (ER) and late endosomes/lysosomes (Le-Lyso), and association of CdtV-A and CdtV-C with OMVs after 20 h determined by CLSM. The indicated single fluorescence channels are shown in the right panels and the merged images in the left panels (green, OMV or CdtV-A or CdtV-C, as indicated; red, OMVs or compartment-specific marker proteins, as indicated; blue, nuclei; yellow, colocalized green and red signals). The percentages of colocalizations of the respective signals (white numbers) were calculated with the BioImageXD6 tool (means of colocalizations from three different samples are shown). Scale bars are 10 μm. (D) Controls to CLSM data shown in Fig 11C. Separation of CdtV-B from OMVs, and detection of CdtV-B in the Golgi complex, endoplasmic reticulum (ER) and late endosomes/lysosomes (Le-Lyso) of HBMEC postincubated for the times indicated with OMVs BL21(*cdt*V-*ABC*) carrying CdtV holotoxin (positive control), or with CdtV-B lacking OMVs from *cdt*V-*B* deletion mutant BL21(*cdt*V-*ACΔB*) (negative control). The CLSM data were analyzed and are presented as given in B and C. Green, CdtV-B; red, OMV or compartment-specific marker proteins, as indicated; blue, nuclei; yellow, colocalized green and red signals (the percentages of colocalizations are shown by white numbers). (E, F) Immunoblot detection of CdtV-A (E) and CdtV-C (F) in isolated endoplasmic reticulum (ER) and lysosomes (Lyso) of HBMEC incubated with the indicated OMVs for 20 h. BL21(*cdt*V-*ABC*) OMVs were a positive control and BL21(pET23) OMVs and untreated cells (no OMV) negative controls; lanes OMV ctrl contain 5791/99 OMVs without cells.
(TIF)

S29 Fig Enlarged single fluorescence channels of CLSM images shown in Fig 11. (A) Images shown in Fig 11B. (B) Images shown in Fig 11C, time points 90 min and 4 h. Scale bars are 10 μm. Le-Lyso, late endosomes/lysosomes; ER, endoplasmic reticulum.
(TIF)

S30 Fig Enlarged single fluorescence channels of CLSM images shown in Fig 11. (A) Images shown in Fig 11C, time point 20 h. (B) Images shown in Fig 11D. Scale bars are 10 μm. Le-Lyso, late endosomes/lysosomes; ER, endoplasmic reticulum.
(TIF)

S31 Fig Intracellular trafficking of OMV O157-delivered EHEC-Hly. (A) CLSM of HBMEC preincubated with OMVs 5791/99 for 30 min at 4°C, and postincubated at 37°C for the times indicated. The indicated single fluorescence channels are shown in the right panels and the merged images in the left panels (green, EHEC-Hly (EHly); red, compartment-specific marker proteins; blue, nuclei; yellow, colocalized green and red signals). The percentages of EHEC-Hly colocalizations with compartment-specific marker proteins (white numbers) and with nucleus (blue numbers in panels ER) were calculated with the BioImageXD6 tool. Scale bars are 10 μm. (B) Graphical summary of EHEC-Hly colocalizations with subcellular compartments based on CLSM data shown in A, and with OMVs (based on data shown in Fig 3A). (Means of colocalizations from at least five different samples are shown in A and B; for standard deviations and significance analysis see S8G Fig). (C) Immunoblot detection of EHEC-Hly in isolated subcellular fractions of HBMEC which were incubated for the times indicated with 5791/99 OMVs, or for 72 h without OMVs or with TRIS-HCl OMV buffer (negative controls); 5791/99 OMVs without cells were a positive control. (D) Densitometric quantification of EHEC-Hly signals shown in C. Abbreviations: Ee, early endosomes; Le/Lyso, late endosomes/lysosomes; ER, endoplasmic reticulum; Mito, mitochondria; Nucl, nucleus; Cyto, cytoplasm.
(TIF)

S32 Fig OMV-delivered CdtV causes G2 arrest of HBMEC and Caco-2 cells. Flow cytometry histograms of (A) HBMEC and (C) Caco-2 cells treated for 24 h to 96 h with O157 OMVs or OMVs from CdtV-positive control strain TA153. (B) HBMEC and (D) Caco-2 cells treated for 24 h or 96 h with CdtV-negative OMVs from strain TA154 (vector control) or left untreated (negative controls). Positions of the G1 (2n DNA) and G2 (4n DNA) peaks are indicated in the first histograms in A and C. The proportions (%) of cells in G1 and G2 cell cycle phase, respectively, are shown in all histograms. Arrows depict sub-G1 populations (apoptotic cells).
(TIF)

S33 Fig Dose-dependence of G2 arrest caused by CdtV-containing OMVs. (A) Caco-2 cells, (B) HBMEC, and (C) HRGEC were incubated for 24 h (HRGEC) or 48 h (Caco-2, HBMEC) with two-fold dilutions of O157 (5791/99, 493/89, 493/89Δ*stx*_2a_) or TA153 OMVs containing the indicated amounts of CdtV. Proportions of cells in G2 arrest (4n DNA content) were determined by flow cytometry. Data are means ± standard deviations from three independent experiments. *p < 0.05, **p < 0.01 or ***p < 0.001 (one-way ANOVA) for G2 arrest caused by the indicated CdtV doses compared to untreated cells (cell control).
(TIF)

S34 Fig Gb3 is required for apoptosis caused by OMV-delivered Stx2a. (A) Presence of Stx2a and Stx1a in EDL933 OMVs as detected by immunoblot. (B) EDL933 OMVs are internalized by Gb3-negative DLD-1 cells after 4 h of incubation as determined by CLSM. Green, OMVs; red, actin; blue, nuclei. Confocal Z-stack projections are included at upper/right sides. Crosshairs show the position of the xy and yz planes. (C) Colocalizations of EDL933 OMVs and OMV-delivered Stx2a with late endosomes/lysosomes (Le-Lyso) and the endoplasmic reticulum (ER) after 4 h of incubation of the OMVs with DLD-1 cells. The indicated single fluorescence channels are shown in the right panels and the merged images in the left panels (green, Stx2a or OMVs, as indicated; red, compartment-specific marker proteins, as indicated; blue, nuclei; yellow, colocalized green and red signals). The percentages of colocalization of the respective signals (white numbers) were calculated with the BioImageXD6 tool (means of colocalizations from three different samples are shown). Scale bars in B and C are 10 μm. (D, E) Immunoblot detection of Stx2a (D) and Stx1a (E) in isolated endoplasmic reticulum (ER) and lysosomal (Lyso) fractions of DLD-1 cells which were incubated for 4 h with EDL933 OMVs or left untreated (no OMV) (negative control). EDL933 OMVs without cells were a positive control (OMV ctrl). (F) Apoptosis caused in DLD-1 cells by the indicated samples after 96 h of incubation as determined by Cell Death Detection ELISA. Enrichment factors were calculated by dividing OD_405_ absorbance values of sample-treated cells with those of untreated cells. Staurosporine (1 μM) was a positive control and OMV buffer and free Stx2a which is not internalized by DLD-1 cells (Fig 6E) negative controls. *p < 0.05, apoptosis significantly higher than that caused by OMV buffer (one-way ANOVA).
(TIF)

S35 Fig Immunoblot detection of Stx2a A and B subunits in O157 OMVs and purified Stx2a. Samples were separated by SDS-PAGE and subjected to immunoblot with rabbit anti-Stx2a antibody (He et al., 2013). Sizes of Stx2a A and B subunits are indicated on the right side.
(TIF)

S36 Fig CLSM controls. (A) HBMEC were incubated for 20 h with OMV buffer (20 mM TRIS-HCl) instead of OMVs. Cells were stained with rabbit anti-*E*. *coli* O157 LPS antibody and Alexa Fluor 488-conjugated goat anti-rabbit IgG or with mouse anti-*E*. *coli* O157 antibody and Cy3-conjugated goat anti-mouse IgG to detect OMVs. To detect virulence proteins, cells were stained with anti-Stx2a, anti-CdtV-A, -B, -C, or anti-EHEC-Hly rabbit antibodies and Alexa Fluor 488-conjugated goat anti-rabbit IgG. (B) HBMEC were incubated with 5791/99 OMVs for 20 h and stained with Cy3-conjugated goat anti-mouse IgG and with Alexa Fluor 488-conjugated goat anti-rabbit IgG, or with Alexa Fluor 488-conjugated goat anti-rat IgM in the absence of first antibodies. (C) DLD-1 cells were incubated for 4 h with 5791/99 OMVs and stained with Alexa Fluor 488-conjugated goat anti-rat IgM or with Cy3-conjugated goat anti-mouse IgG and Alexa Fluor 488-conjugated goat anti-rabbit IgG in the absence of first antibodies. Nuclei in all panels were stained with DRAQ5. Scale bars are 10 μm.
(TIF)

S1 Table Wild-type and recombinant *E*. *coli* strains used in this study. (PDF)

S2 Table Proteins identified in OMVs from NSF *E*. *coli* O157:H7 strain 5791/99 using nano-LC-MS/MS. (PDF)

S3 Table Proteins identified in OMVs from SF *E*. *coli* O157:H^-^ strains 493/89 and 493/89Δ*stx*_2a_ using nano-LC-MS/MS. (PDF)

S4 Table Concentrations of the total protein and OMV-associated virulence factors in OptiPrep-purified OMVs from *E*. *coli* O157:H7/H^-^ strains. (PDF)

S5 Table PCR primers used for restriction-free cloning of *cdt*V genes and construction of *cdt*V*-B* deletion mutant. (PDF)

S1 Text Expression and purification of free CdtV-B. (PDF)
