# Supplementary figures and images for: Host cell interactions of outer membrane vesicle-associated virulence factors of enterohemorrhagic Escherichia coli O157: Intracellular delivery, trafficking and mechanisms of cell injury
Source: PLoS Pathog. 2017 Feb 3;13(2):e1006159. doi: 10.1371/journal.ppat.1006159 (PMC5310930; doi:10.1371/journal.ppat.1006159)

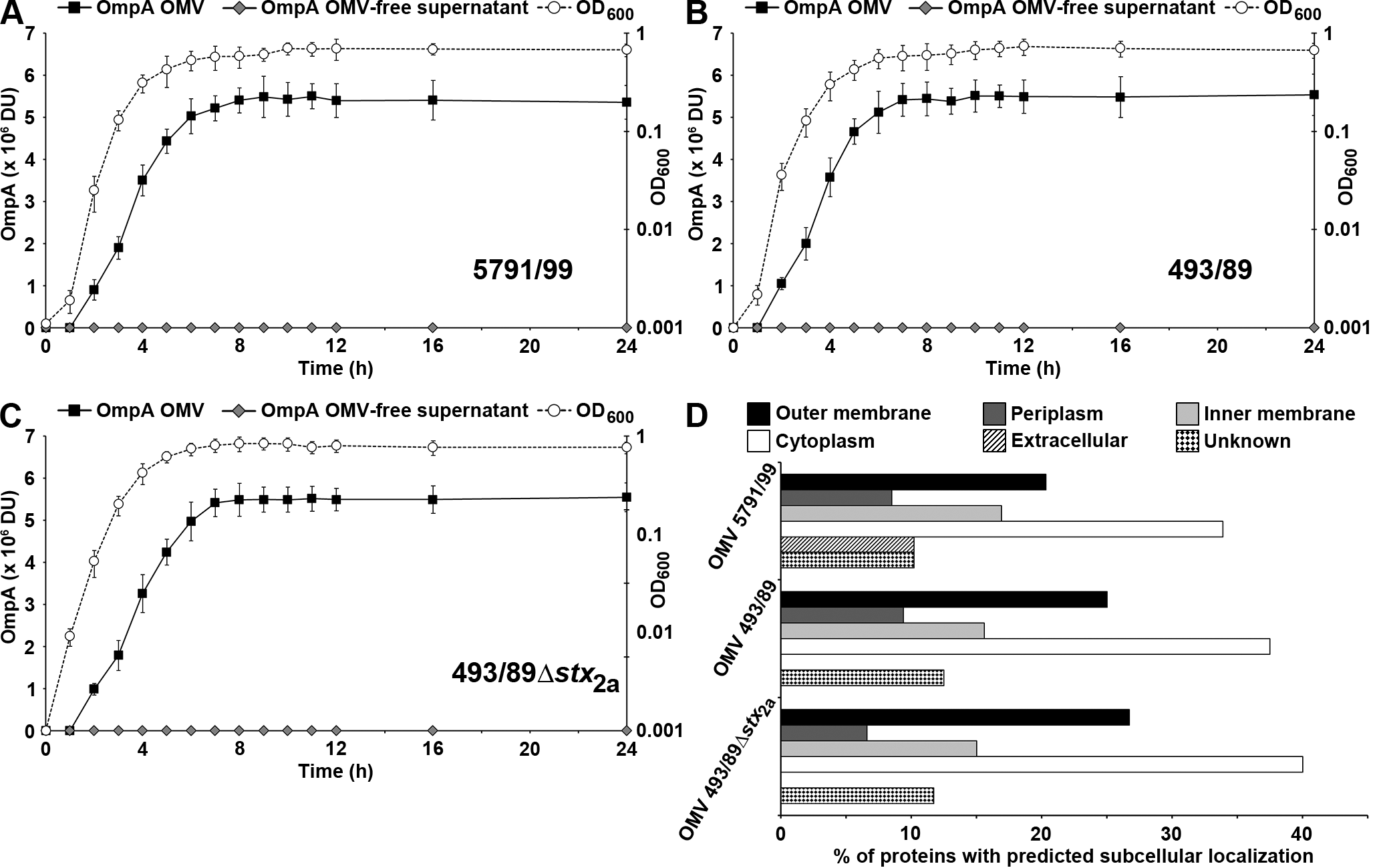

Supplement: S1 Fig — (A, B, C) Strains were grown in LB broth, OMVs were isolated at indicated times, subjected to immunoblot with anti-OmpA antibody, and quantified by densitometry of OmpA signals (expressed in arbitrary densitometric units; DU). OMV-free supernatants served as controls. Bacterial growth was monitored by measuring OD600. Data are means ± standard deviations from three independent experiments. (D) Distribution of OMV-associated proteins identified with nano-LC-MS/MS according to their subcellular localization determined by PsortB prediction tool. (TIF) [file ppat.1006159.s001.tif]

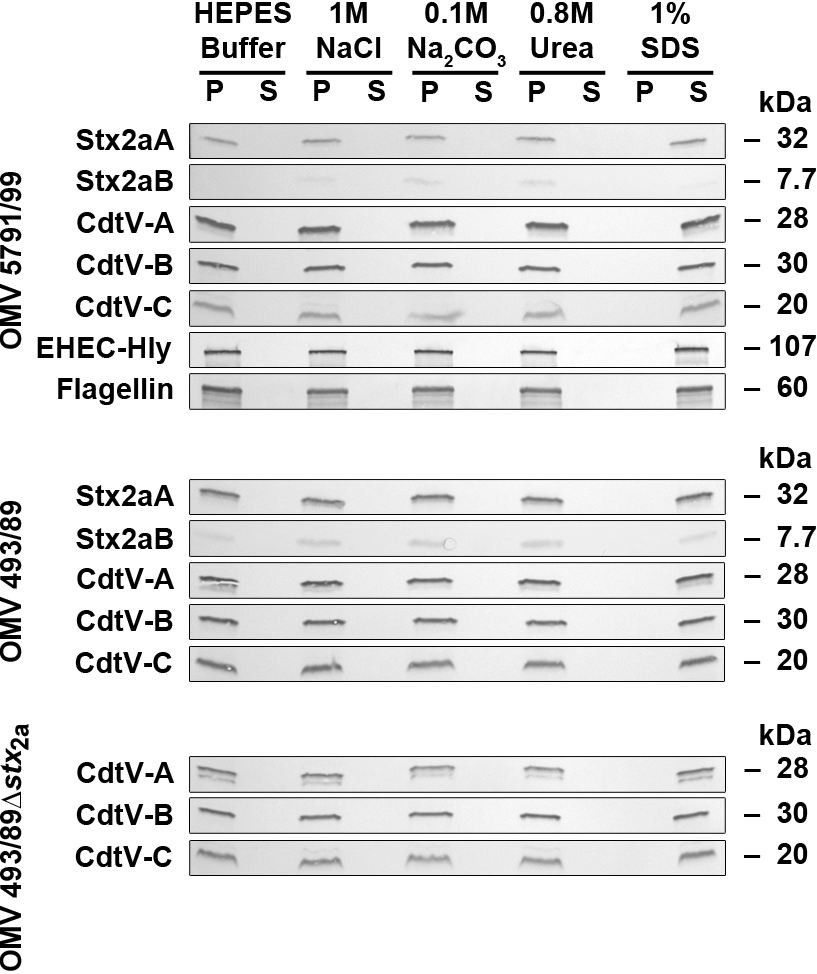

Supplement: S2 Fig — OptiPrep-purified OMVs from strains 5791/99, 493/89, and 493/89Δstx2a were incubated in HEPES buffer alone (control), or in HEPES buffer with the indicated chemicals. After ultracentrifugation, pellets (P; containing OMVs) and supernatants (S; containing proteins released from OMVs) were analyzed by immunoblot with antibodies against the virulence factors or their subunits. (TIF) [file ppat.1006159.s002.tif]

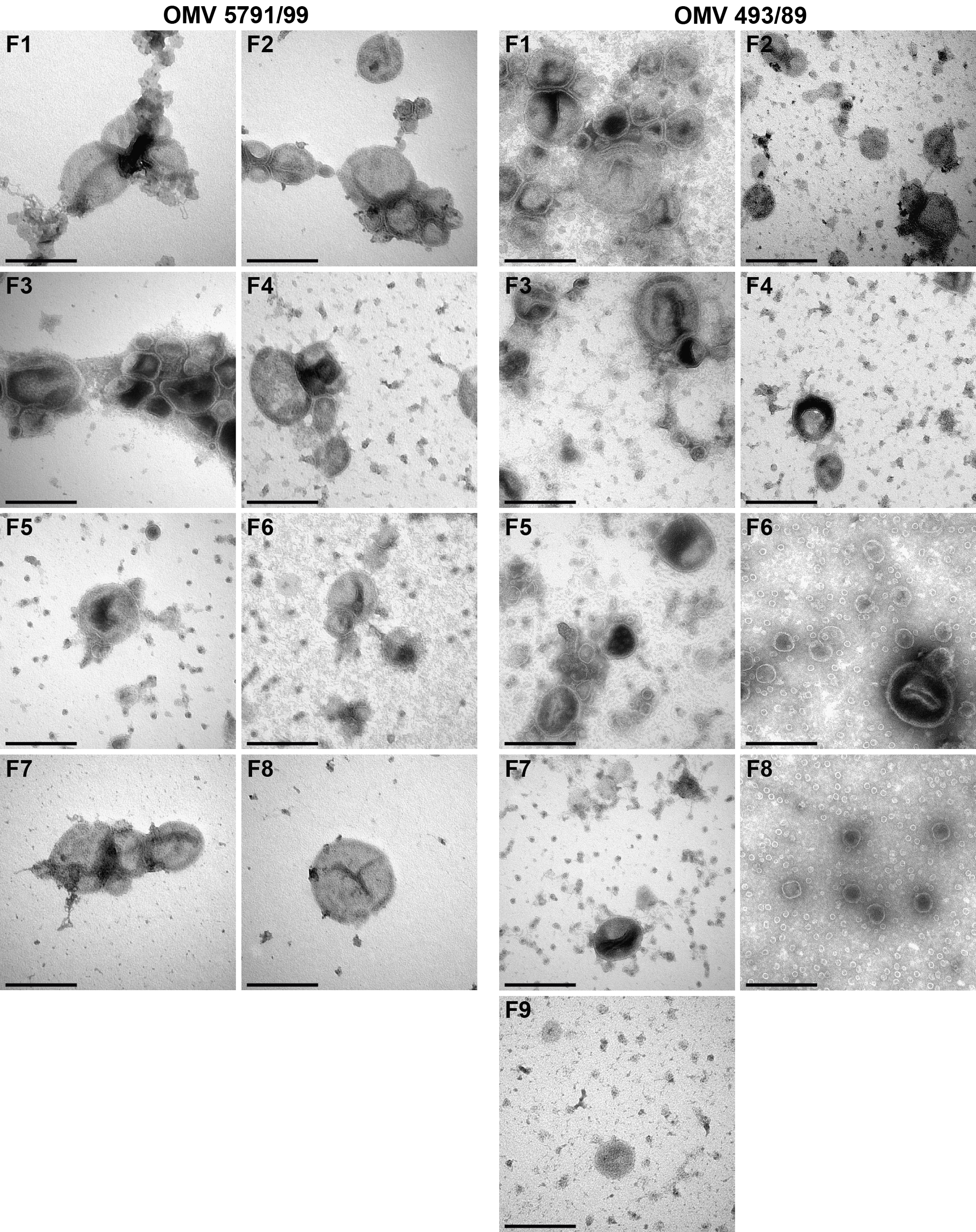

Supplement: S3 Fig — OptiPrep gradient fractions of 5791/99 OMVs (F1 –F8) and 493/89 OMVs (F1 –F9) were negatively stained with 0.5% uranyl acetate and analyzed with a FEI-Tecnai 12 electron microscope. Scale bars are 200 nm. (TIF) [file ppat.1006159.s003.tif]

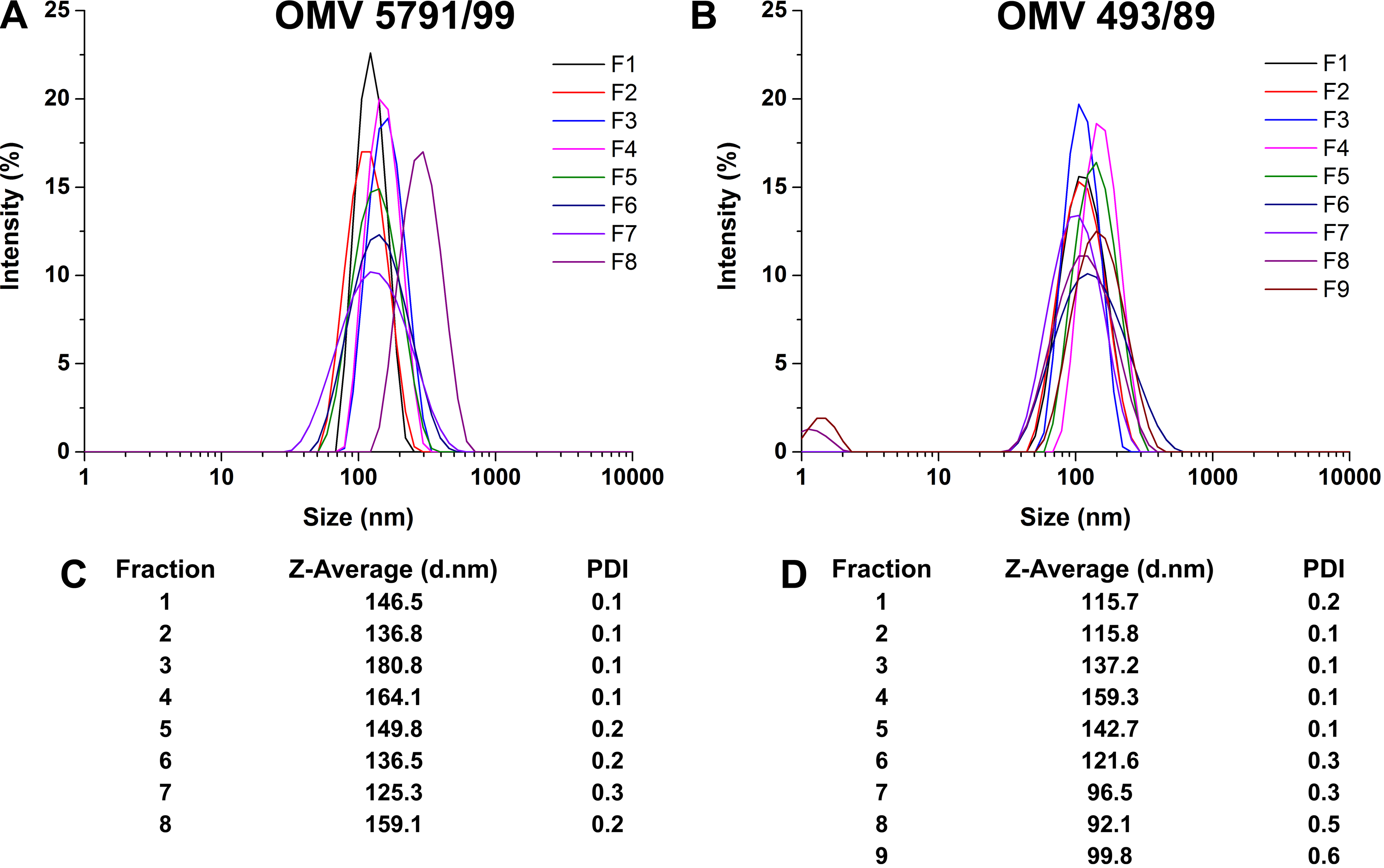

Supplement: S4 Fig — The x-axis displays the OMV size distribution in each fraction and the y-axis the scattered light intensity of the OMVs. In (C) and (D) the average diameter (Z-average) of OMVs and the index of the particle size distribution (polydispersity index; PDI) in each fraction is shown (d.nm, diameter in nm). The Z-averages and PDIs were calculated by the method of cumulants using the Zetasizer software. (TIF) [file ppat.1006159.s004.tif]

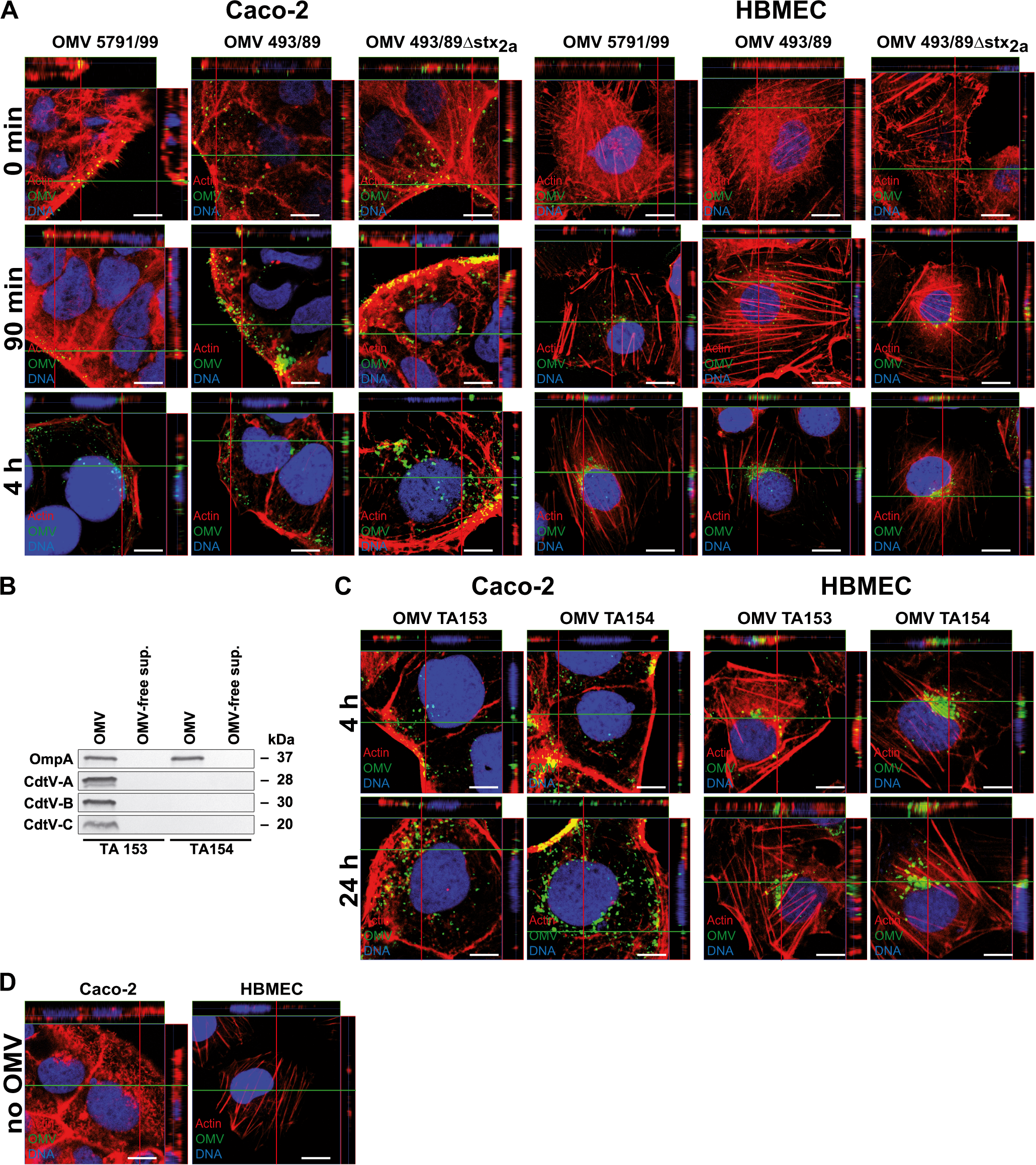

Supplement: S5 Fig — (A, C) CLSM visualization of binding (panels 0 min) and uptake of O157 (A) and TA153 and TA154 OMVs (C) by Caco-2 cells and HBMEC after 90 min, 4 h and 24 h of incubation. OMVs (green) were detected with anti-E. coli O157 LPS (A) or anti-E. coli LPS (C) antibody and Alexa Fluor 488-conjugated goat anti-rabbit IgG, actin (red) with phalloidin-TRITC and nuclei (blue) with DRAQ5. Confocal Z-stack projections are included at upper/right sides. Crosshairs show the position of the xy and yz planes. Scale bars are 10 μm. (D) CLSM of control cells incubated with OMV buffer instead of OMVs for 24 h and stained and processed as described in A and C. (B) Distribution of CdtV-A, CdtV-B, and CdtV-C proteins in OMVs and OMV-free supernatants of strains TA153 (containing the cdtV-ABC operon from strain 493/89 in SuperCos I) and TA154 (vector control) determined by immunoblot with antibodies against OmpA (an OMV marker) and the respective CdtV subunits. (TIF) [file ppat.1006159.s005.tif]

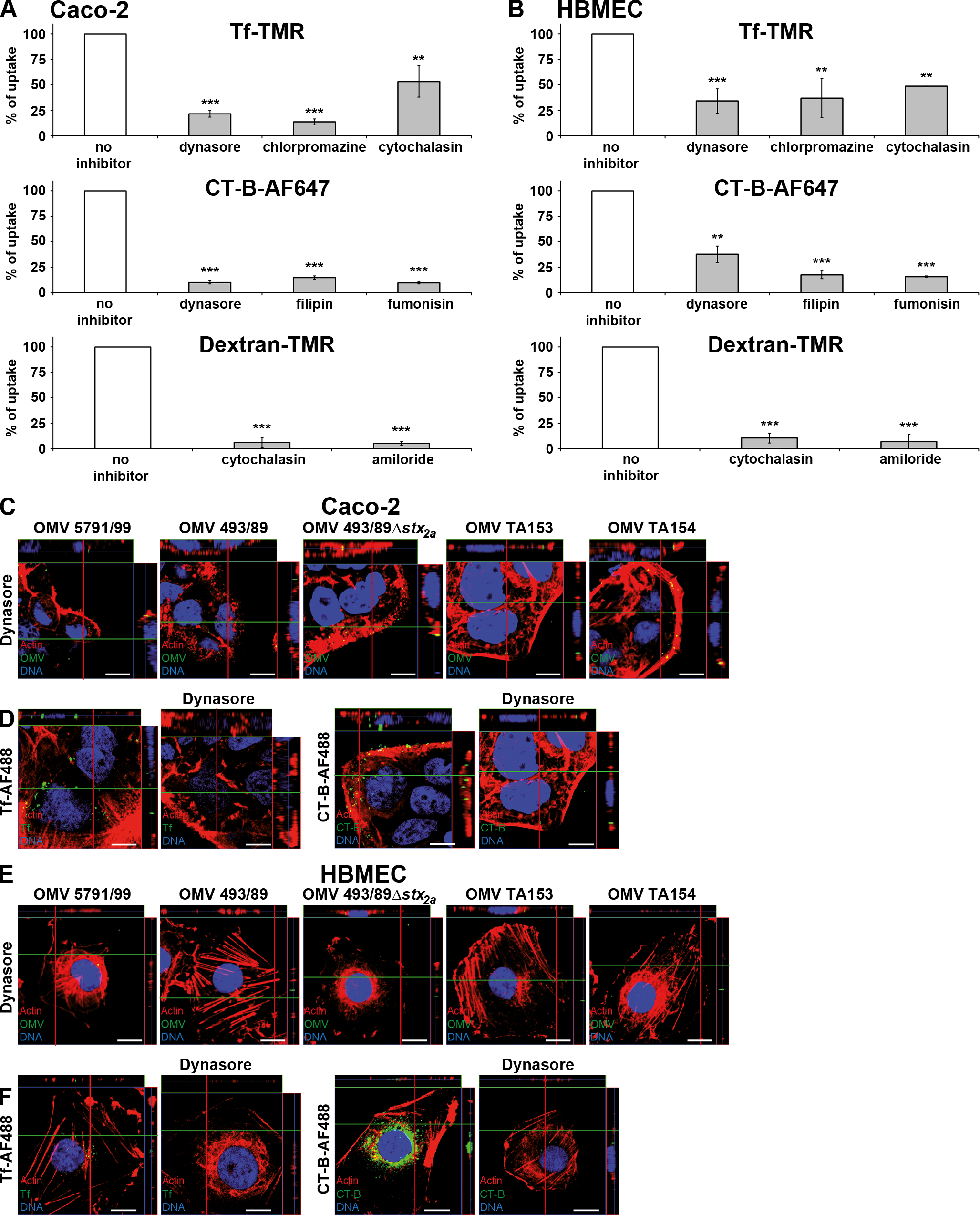

Supplement: S6 Fig — (A, B) Activities of inhibitors of endocytosis used in this study against markers of different endocytosis pathways including clathrin-mediated endocytosis (tetramethylrhodamine-conjugated transferrin; Tf-TMR), lipid rafts/caveolae-mediated endocytosis (Alexa Fluor 647-conjugated cholera toxin B subunit; CT-B-AF647), and macropinocytosis (TMR-conjugated Dextran 10.000; Dextran-TMR). Caco-2 cells (A) and HBMEC (B) either untreated (no inhibitor) or pretreated with the indicated inhibitors were incubated with Tf-TMR, CT-B-AF647 or Dextran-TMR for 4 h and fluorescence was measured with FLUOstar OPTIMA fluorometer. The uptake of each marker in the presence of inhibitors was expressed as the percentage of its uptake by inhibitor-untreated cells (100%). Data are means ± standard deviations from three independent experiments. ** p < 0.01, and *** p < 0.001 compared to inhibitor-untreated cells (one-way ANOVA). (C-F) Effect of dynasore on the uptake of OMVs (C, E) and control endocytosis markers (D, F) by Caco-2 cells (C, D) and HBMEC (E, F) visualized by CLSM after 4 h of incubation of cells with the indicated samples. Panels marked Dynasore show dynasore-pretreated cells. Green, OMVs (C, E) or Alexa Fluor 488-conjugated transferrin (Tf-AF488) or Alexa Fluor 488-conjugated cholera toxin B subunit (CT-B-AF488) (D, F); red, actin; blue, nuclei. Confocal Z-stack projections are included at upper/right sides. Crosshairs show the position of the xy and yz planes. Scale bars are 10 μm. For evaluation of the effect of dynasore on OMV uptake, compare the OMV amounts in dynasore-treated cells (C, E, panels Dynasore) with those of the respective OMVs in dynasore untreated cells (S5A and S5C Fig, panels 4 h). (TIF) [file ppat.1006159.s006.tif]

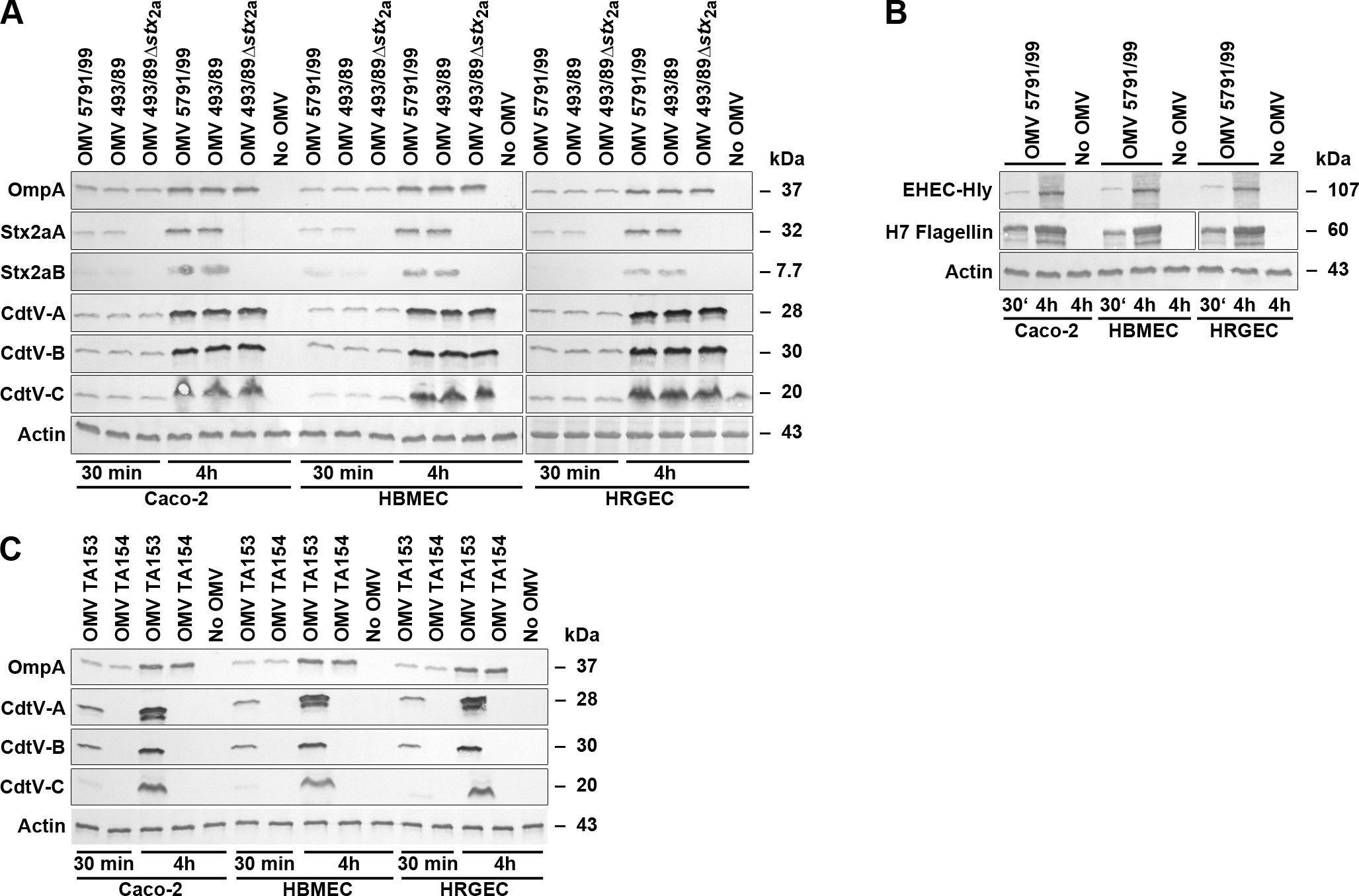

Supplement: S7 Fig — Immunoblot detection of OMVs (anti-OmpA antibody) and OMV-associated virulence factors in lysates of Caco-2 cells, HBMEC, and HRGEC which were incubated with O157 OMVs (A, B) or control CdtV-containing (TA153) or CdtV-lacking (TA154) OMVs (C) for 30 min and 4 h. Untreated cells (no OMV) were negative controls. Actin served as a loading control. (The CdtV-C signal in lane no OMV in the HRGEC lysate in panel A is an artifact resulting from contamination by sample from the previous lane). (TIF) [file ppat.1006159.s007.tif]

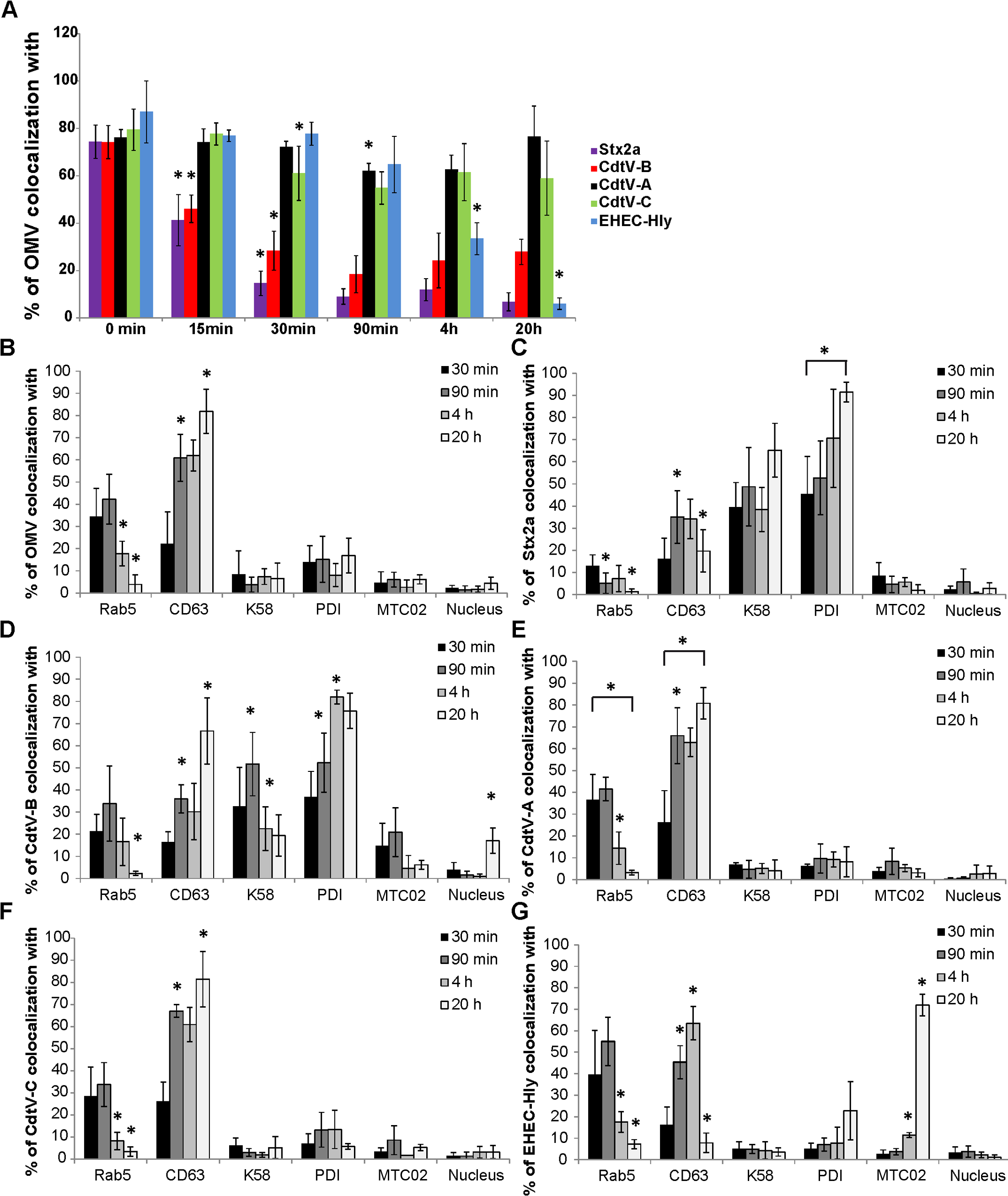

Supplement: S8 Fig — Graphical presentations of CLSM colocalizations between (A) 5791/99 OMVs and OMV-delivered virulence factors, (B) 5791/99 OMVs and subcellular compartments, and (C-G) the indicated OMV-delivered virulence proteins and subcellular compartments during time. The subcellular compartments investigated and their markers were: early endosomes (Rab5), late endosomes/lysosomes (CD63), Golgi complex (K58), endoplasmic reticulum (PDI), mitochondria (MTC02), and nucleus (DNA). The percentages of colocalizations between signals of interest were determined with the BioImageXD6 tool. Data are shown as means ± standards deviations from measurements of at least five (for CdtV-A/CdtV-C of at least three) different samples. *significantly increased or decreased (p < 0.05; two-tailed unpaired Student’s t-test) compared to the previous time interval or between the spanned time intervals. (TIF) [file ppat.1006159.s008.tif]

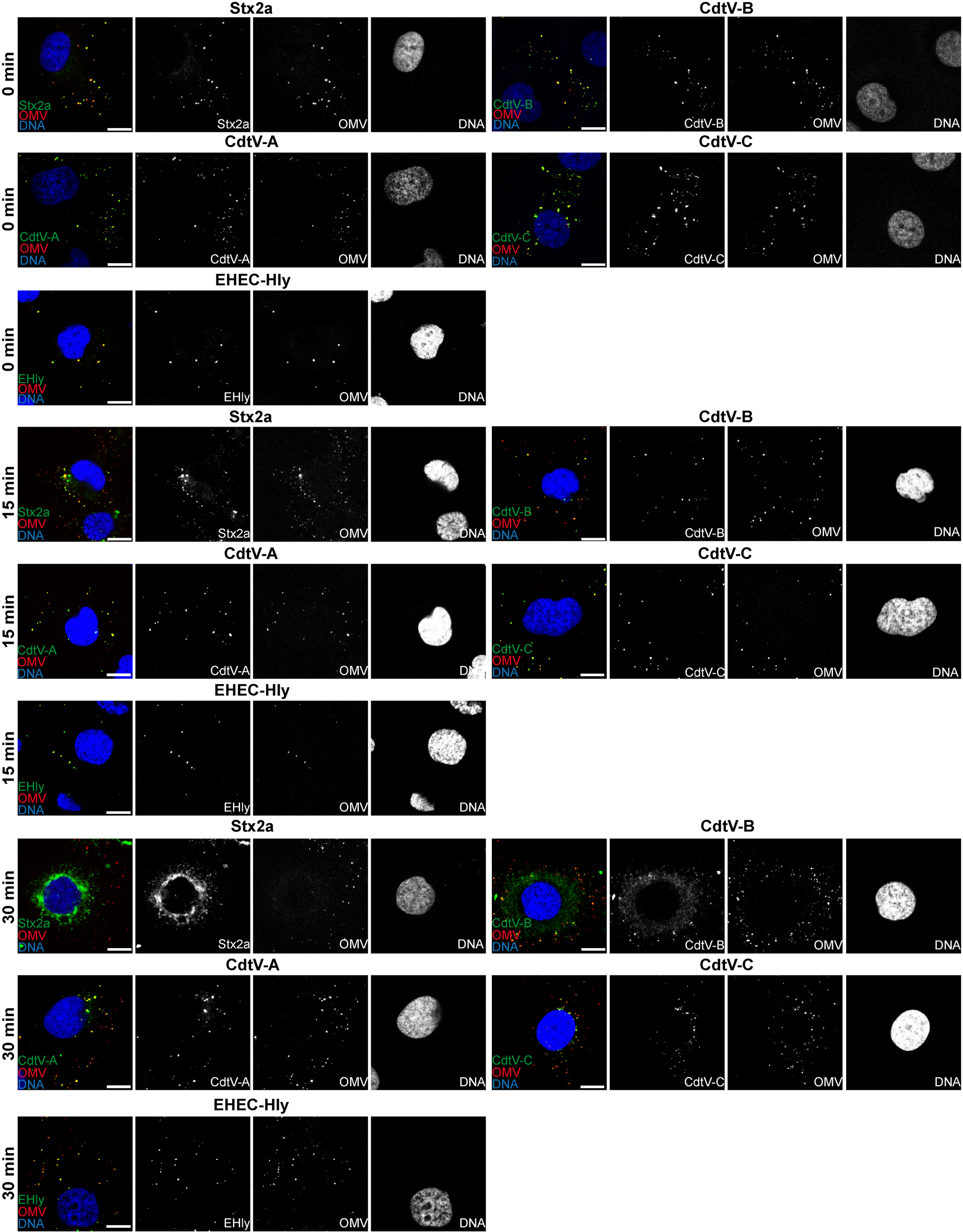

Supplement: S9 Fig — Scale bars are 10 μm. (TIF) [file ppat.1006159.s009.tif]

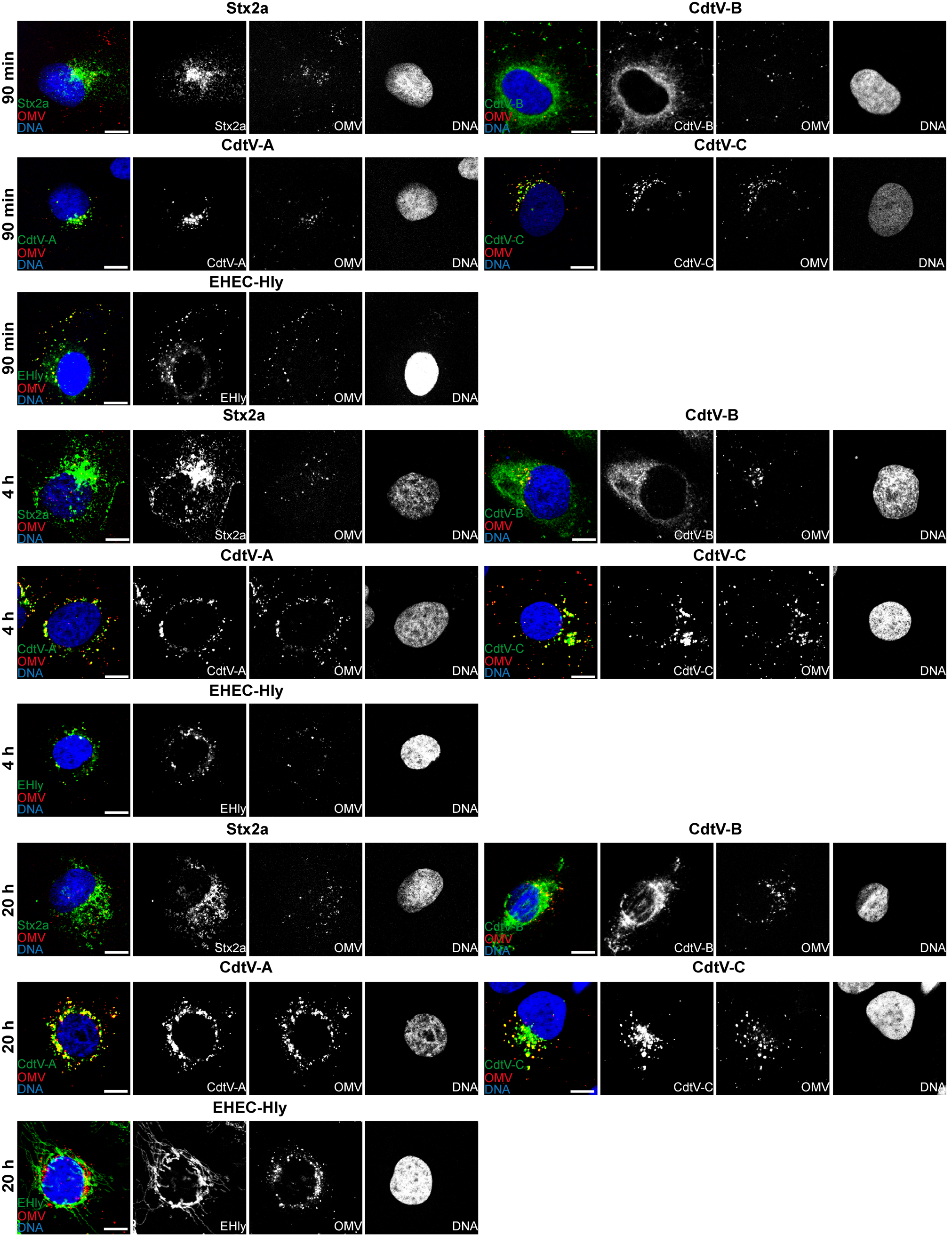

Supplement: S10 Fig — Scale bars are 10 μm. (TIF) [file ppat.1006159.s010.tif]

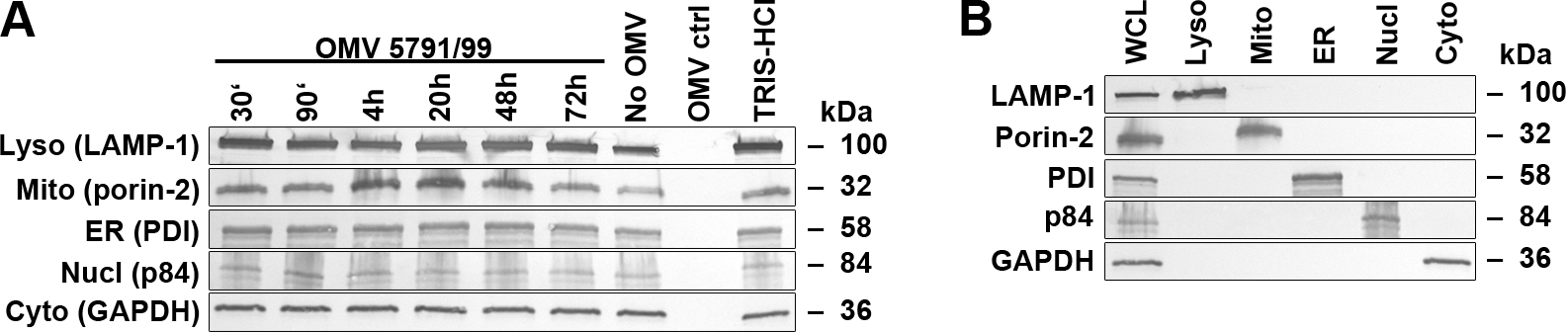

Supplement: S11 Fig — (A) Lysosomal (lyso), mitochondrial (mito), endoplasmic reticulum (ER), nuclear (nucl), and cytosolic (cyto) fractions were isolated from HBMEC which had been incubated for the indicated times with 5791/99 OMVs or for 72 h with OMV buffer (20 mM TRIS-HCl, pH 8.0) or left untreated (no OMV). The fractions were analyzed by immunoblot with antibodies against the indicated compartment-specific marker proteins. 5791/99 OMVs without cells (OMV ctrl) served as a negative control. (B) Whole cell lysates (WCL) (positive control) and subcellular fractions prepared from HBMEC exposed to 5791/99 OMVs for 20 h were analyzed by immunoblot with antibodies against homologous and heterologous compartment-specific marker proteins. (TIF) [file ppat.1006159.s011.tif]

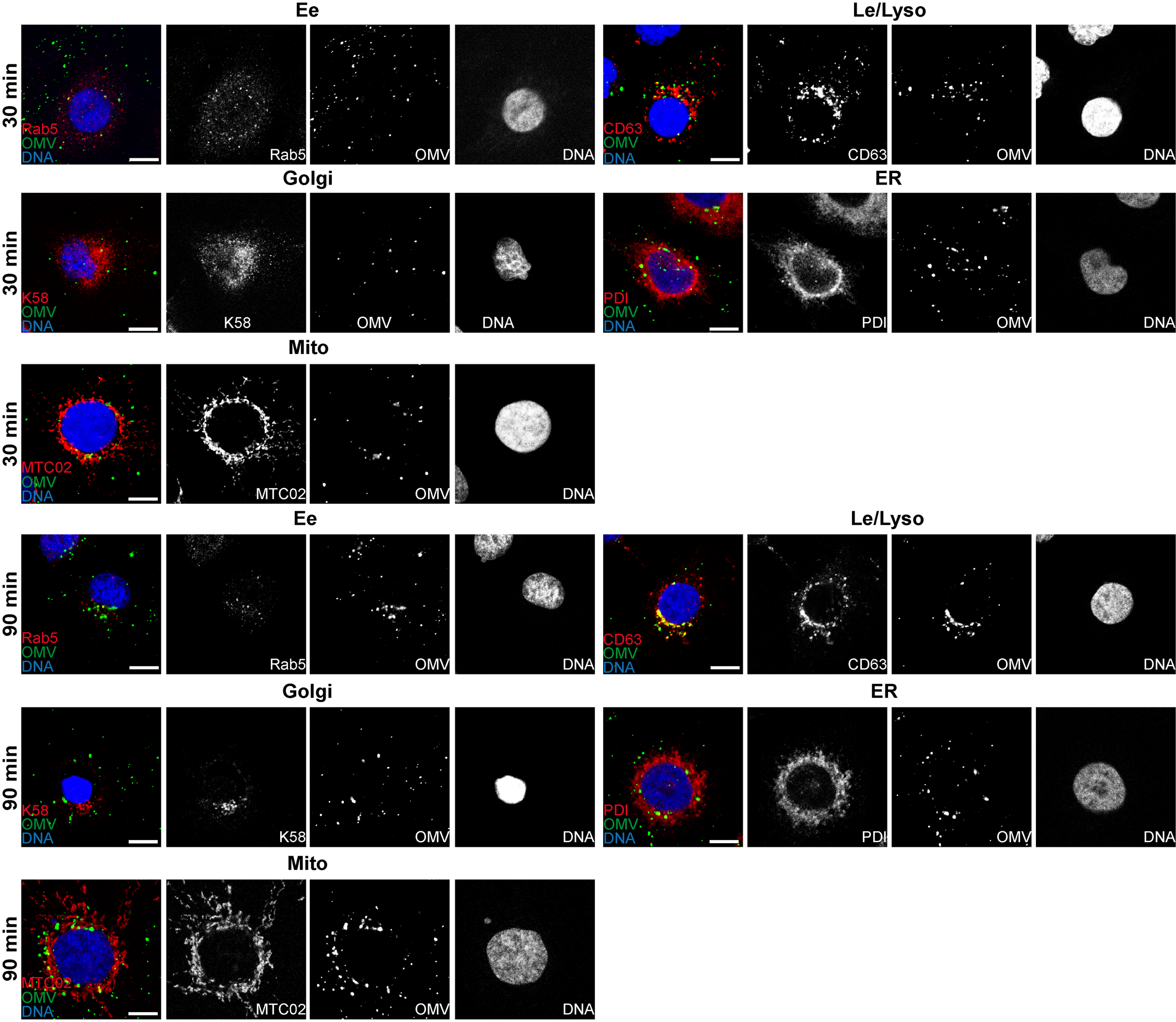

Supplement: S12 Fig — Scale bars are 10 μm. Ee, early endosomes; Le/Lyso, late endosomes/lysosomes; ER, endoplasmic reticulum; Mito, mitochondria. (TIF) [file ppat.1006159.s012.tif]

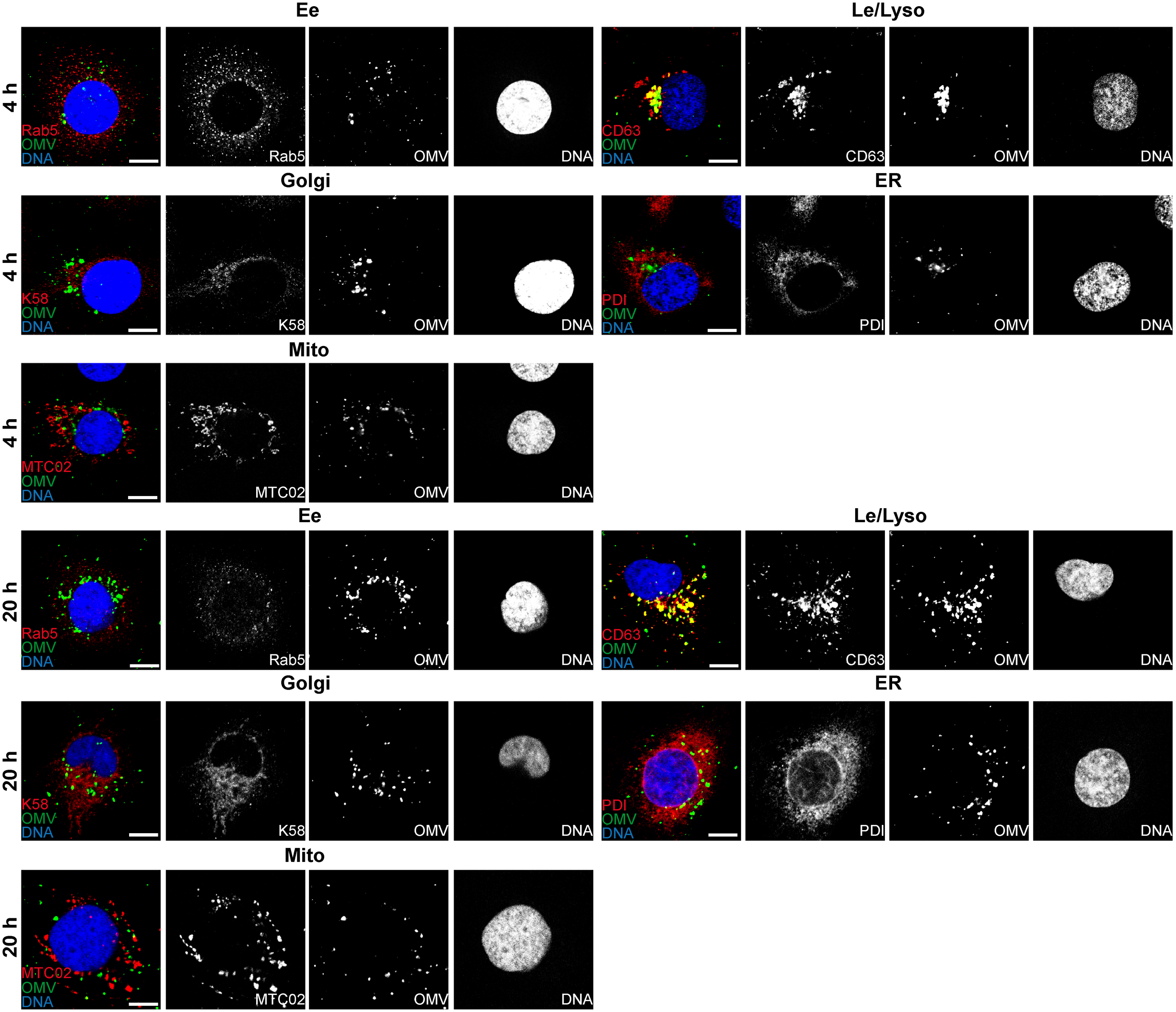

Supplement: S13 Fig — Scale bars are 10 μm. Ee, early endosomes; Le/Lyso, late endosomes/lysosomes; ER, endoplasmic reticulum; Mito, mitochondria. (TIF) [file ppat.1006159.s013.tif]

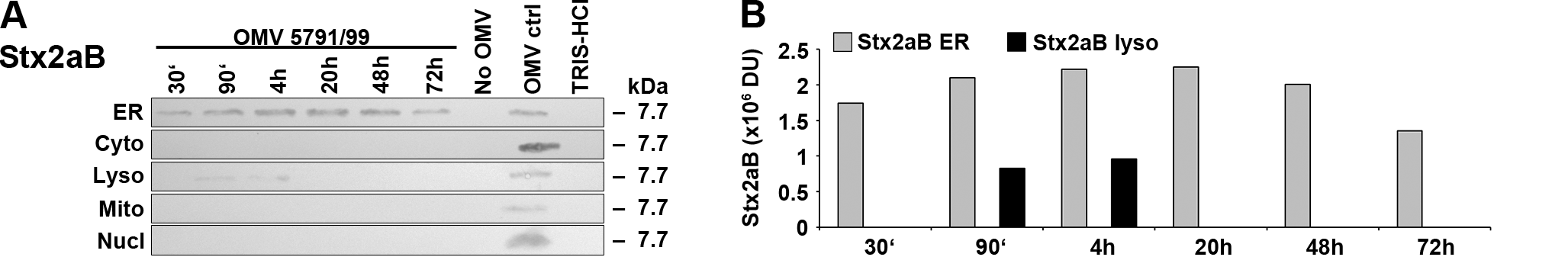

Supplement: S14 Fig — (A) Immunoblot detection of Stx2a B subunit in isolated subcellular fractions of HBMEC which were incubated for the times indicated with 5791/99 OMVs or for 72 h without OMVs or with OMV buffer (20 mM TRIS-HCl) (negative controls); OMVs without cells (OMV ctrl) served as a positive control. (B) Densitometric quantification of Stx2aB signals in endoplasmic reticulum and lysosomal fractions shown in A. Abbreviations used: ER, endoplasmic reticulum; Cyto, cytosol; Lyso, lysosomes; Mito, mitochondria; Nucl, nucleus; DU, densitometric unit. (TIF) [file ppat.1006159.s014.tif]

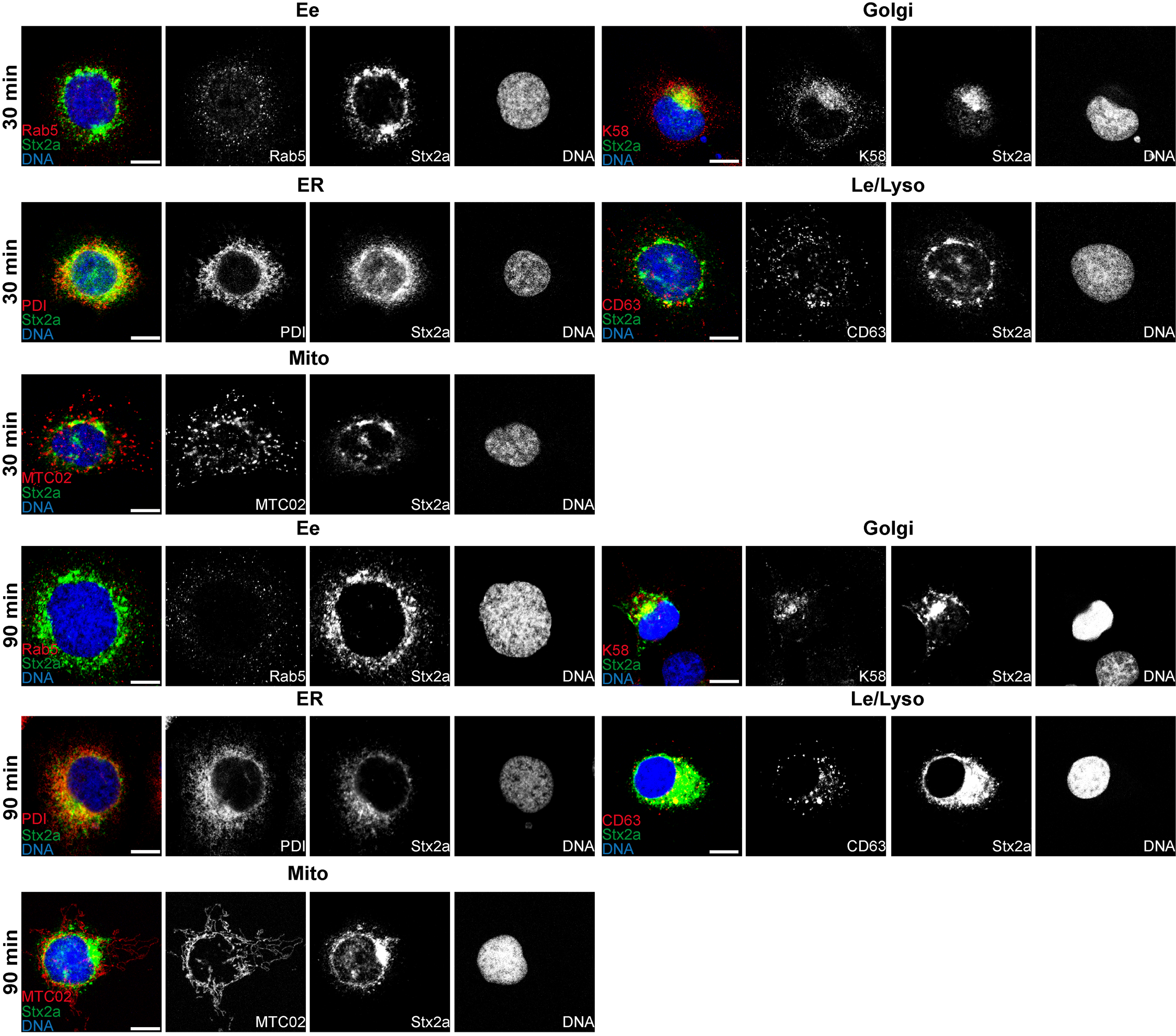

Supplement: S15 Fig — Scale bars are 10 μm. Ee, early endosomes; ER, endoplasmic reticulum; Le/Lyso, late endosomes/lysosomes; Mito, mitochondria. (TIF) [file ppat.1006159.s015.tif]

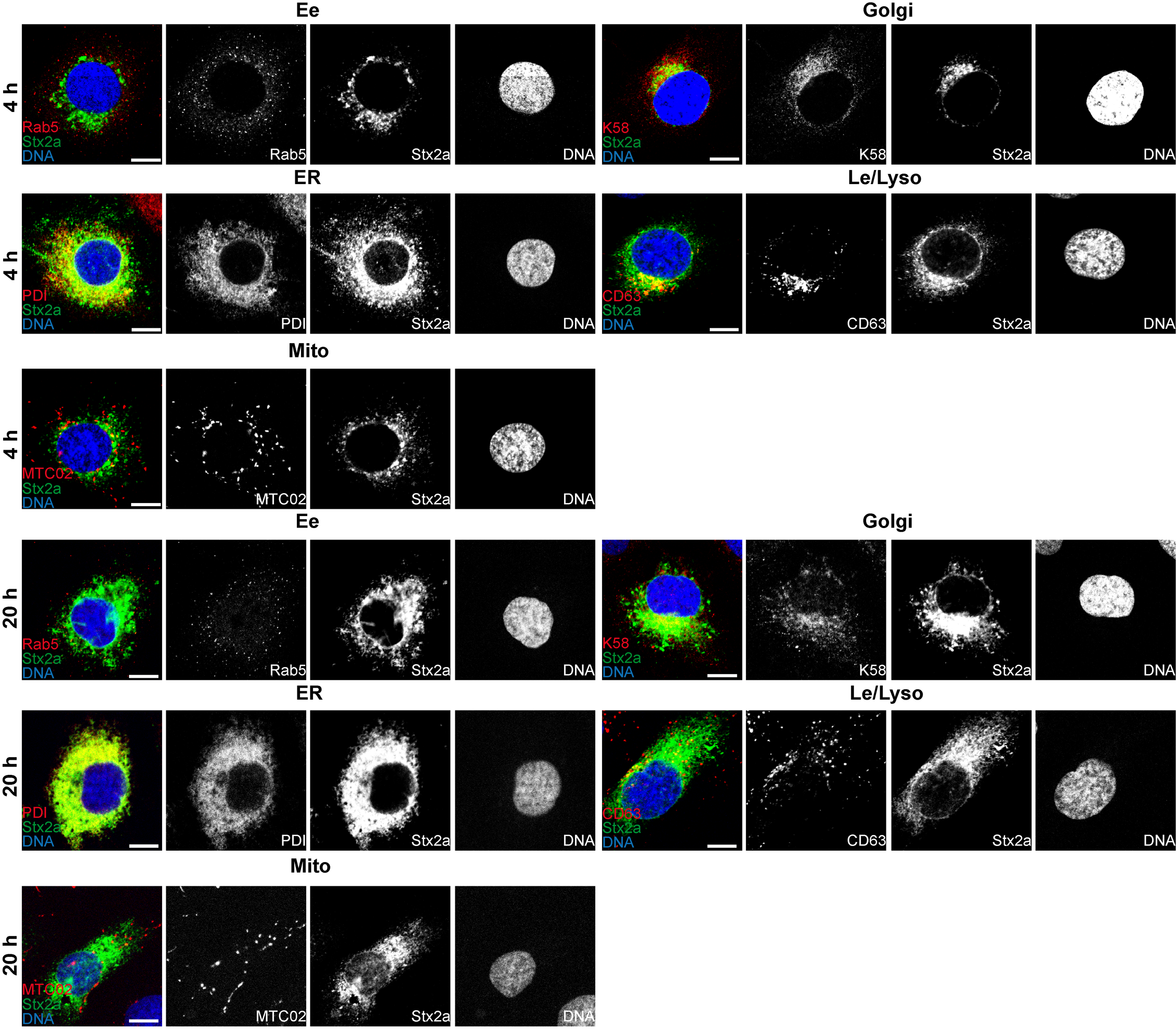

Supplement: S16 Fig — Scale bars are 10 μm. Ee, early endosomes; ER, endoplasmic reticulum; Le/Lyso, late endosomes/lysosomes; Mito, mitochondria. (TIF) [file ppat.1006159.s016.tif]

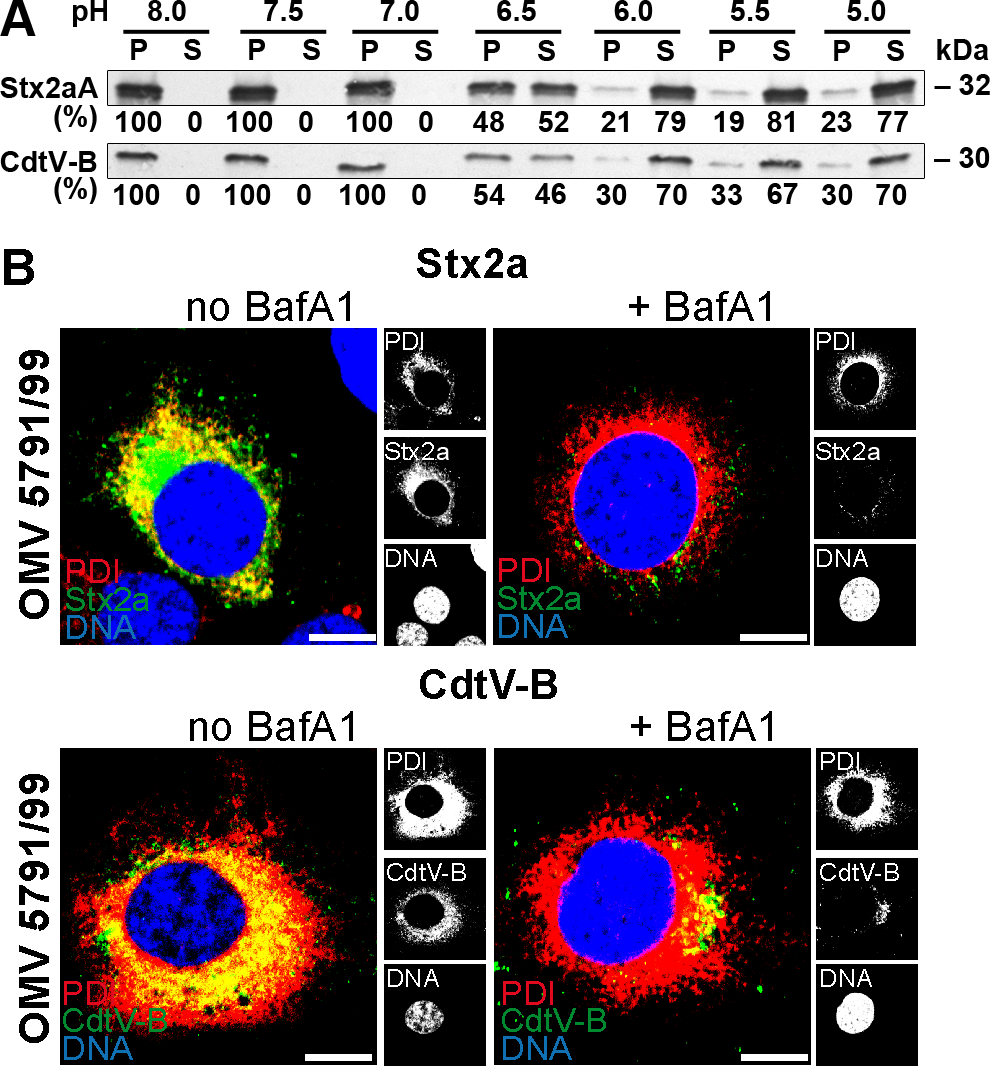

Supplement: S17 Fig — (A) 5791/99 OMVs were incubated in TRIS-HCl with the indicated pH range for 1 h and then ultracentrifuged. The pellets (P) containing OMV-associated proteins and supernatants (S) containing proteins that separated from OMVs were analyzed by immunoblot with antibodies against Stx2a or CdtV-B. The Stx2a and CdtV-B signals were quantified densitometrically and the percentage of each protein present in the P and S fraction at each particular pH was calculated from the total signal. (B) HBMEC were not (no BafA1) or were pretreated (+ BafA1) for 1 h with 100 nM bafilomycin A1 (BafA1), incubated for 4 h with 5791/99 OMVs, and analyzed for the presence of Stx2a or CdtV-B in the endoplasmic reticulum by CLSM. The right panels show the indicated single fluorescence channels, and the left panels the merged images (green, Stx2a or CdtV-B; red, PDI; blue, nuclei; yellow, colocalized green and red signals). Scale bars are 10 μm. Note the differences between the intensities of Stx2a and CdtV-B signals, respectively, in BafA1-untreated and BafA1-treated cells. (TIF) [file ppat.1006159.s017.tif]

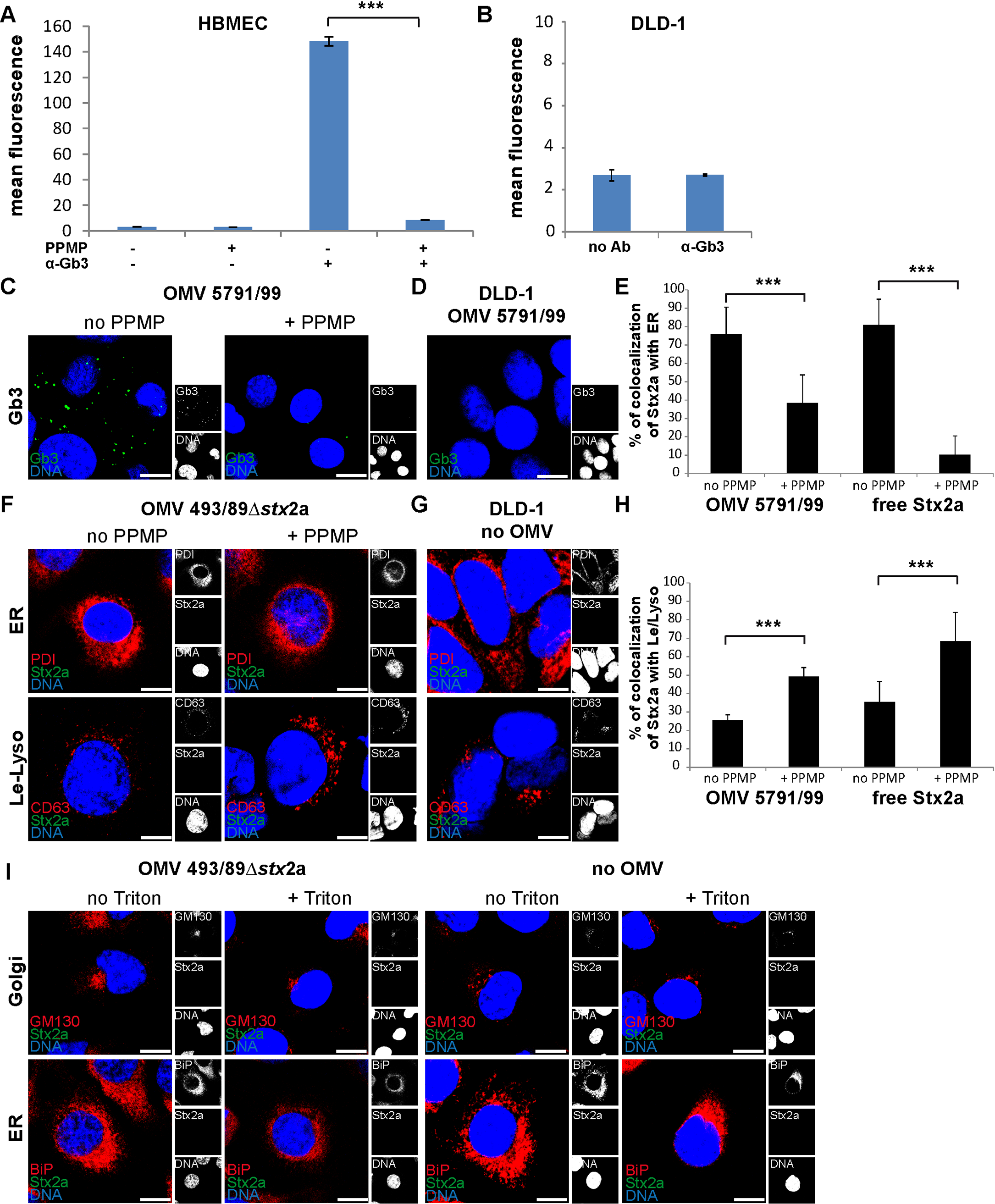

Supplement: S18 Fig — (A, B) Graphical presentations of FACS analyses of Gb3 content in PPMP-untreated (PPMP-) and PPMP-treated (PPMP+) HBMEC (A) and in DLD-1 cells (B) stained with anti-CD77/Gb3-FITC antibody or unstained (control). Geometric mean fluorescence ± standard deviations from three independent experiments are shown. ***p < 0.001 (paired Student´s t-test) for Gb3 content in PPMP-treated compared to PPMP-untreated HBMEC. (C, D) Visualization of Gb3 in PPMP-untreated and PPMP-treated HBMEC (C) and in DLD-1 cells (D) by CLSM. Green, Gb3; blue, nuclei. (F, G) Negative controls to Fig 6B (F) and Fig 6E (G). Note the absence of Stx2a signals (green) in cells exposed to OMVs from Stx2a-negative strain 493/89Δstx2a for 20 h (F) or left untreated (no OMV) (G). (I) Negative controls to Fig 7D. Note the absence of Stx2a signals (green) in cells which had been exposed to OMVs from Stx2a-negative strain 493/89Δstx2a for 90 min or 4 h or left untreated (no OMV) before they were processed for CLSM directly (no Triton) or after 1 min extraction with Triton X-100-containing buffer (+ Triton). In C, D, F, G and I, the indicated single fluorescence channels are shown in the right panels and the merged images in the left panels. Scale bars are 10 μm. (E, H) Graphical presentations of CLSM data shown in Fig 6B. Means ± standard deviations of colocalizations from three different samples are shown. ***p < 0.001 (paired Student´s t-test) for colocalization rates in PPMP-treated compared to PPMP-untreated HBMEC. (TIF) [file ppat.1006159.s018.tif]

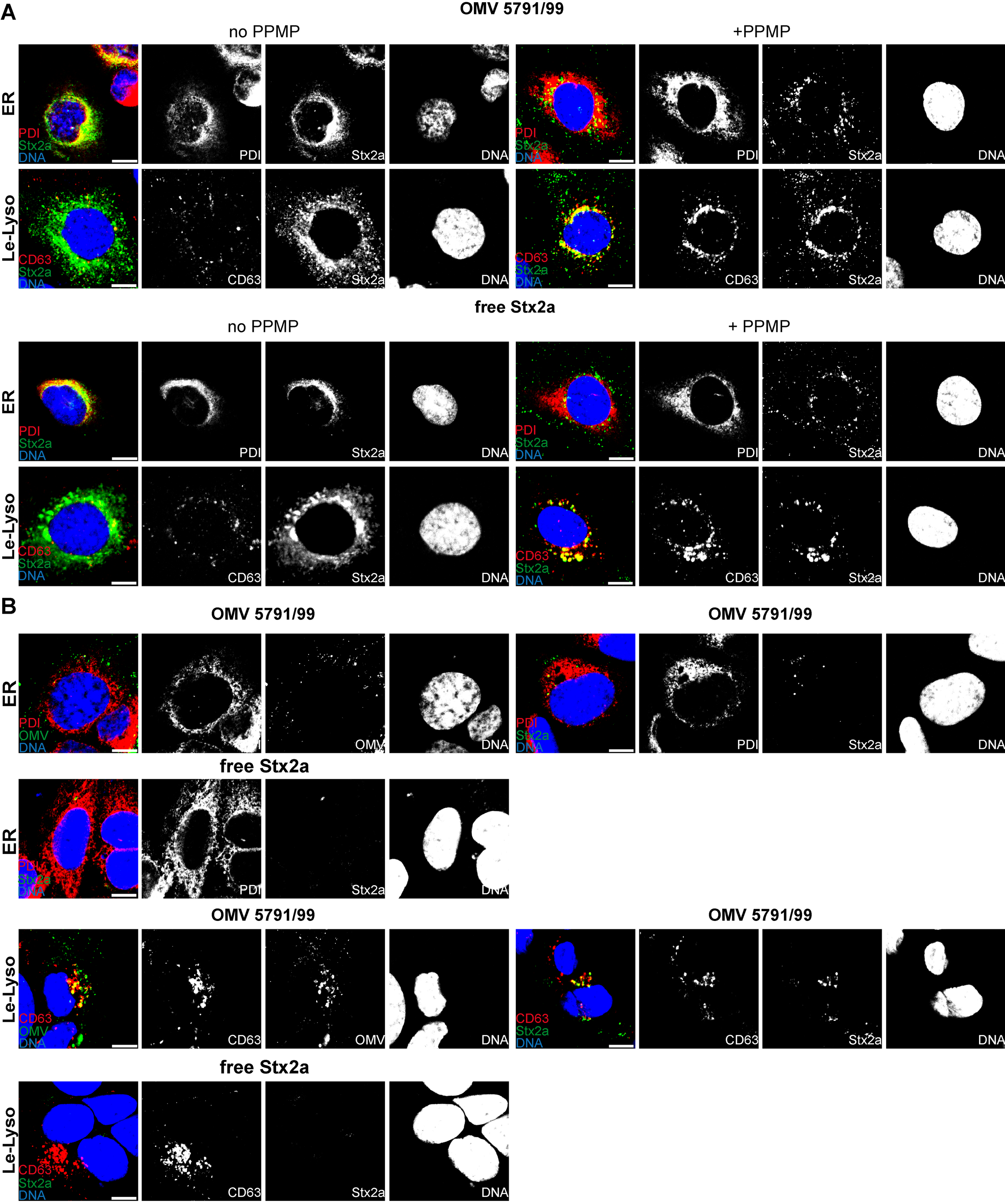

Supplement: S19 Fig — (A) HBMEC (Fig 6B), and (B) DLD-1 cells (Fig 6E). Scale bars are 10 μm. No PPMP, PPMP-untreated HBMEC; +PPMP, PPMP-treated HBMEC. ER, endoplasmic reticulum; Le-Lyso, late endosomes/lysosomes. (TIF) [file ppat.1006159.s019.tif]

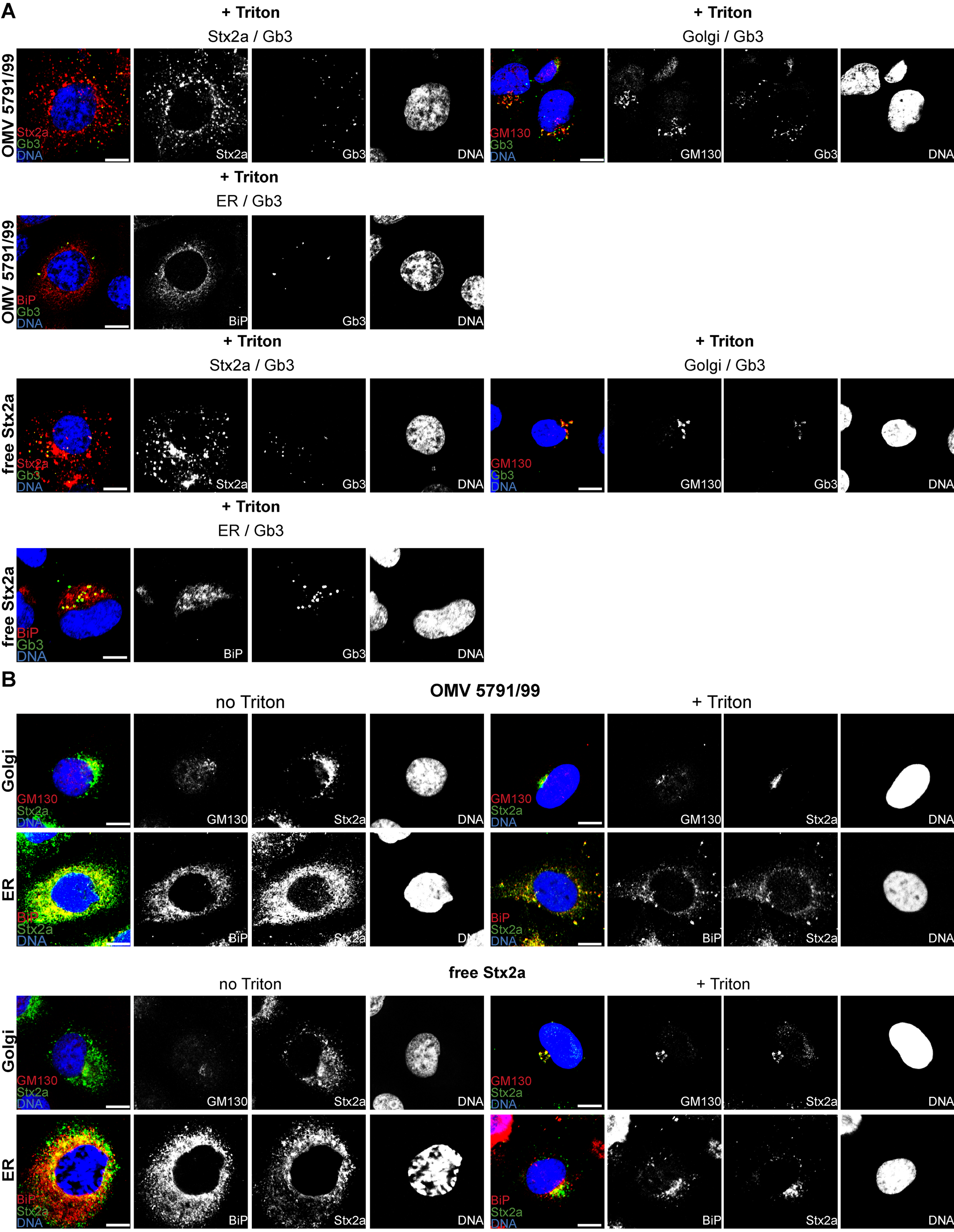

Supplement: S20 Fig — (A) Images shown in Fig 7A, 7B and 7C. (B) Images shown in Fig 7D. HBMEC were processed for CLSM either untreated (no Triton) or after pretreatment with Triton X-100 (+Triton). Scale bars are 10 μm. ER, endoplasmic reticulum. (TIF) [file ppat.1006159.s020.tif]

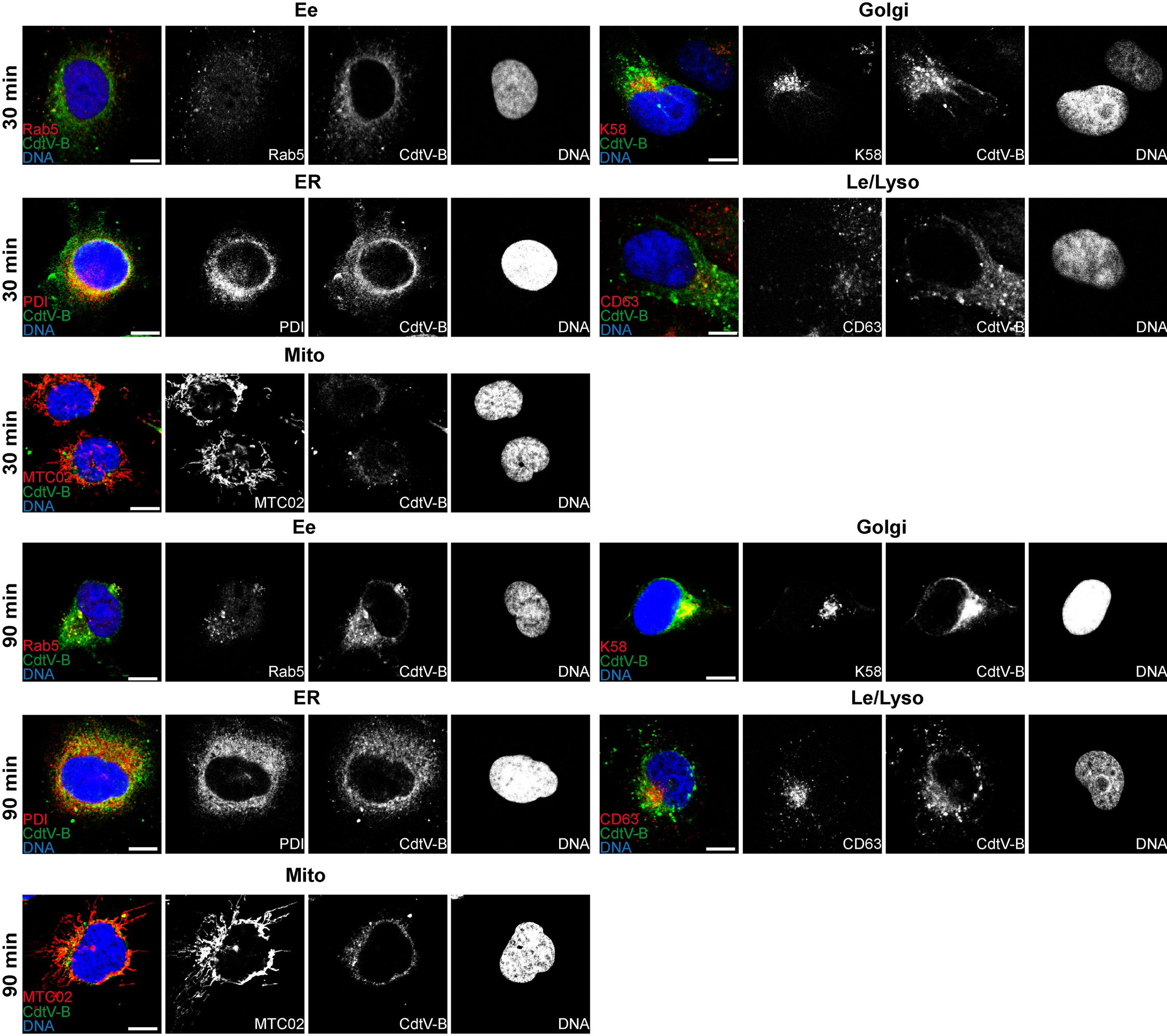

Supplement: S21 Fig — Scale bars are 10 μm. Ee, early endosomes; ER, endoplasmic reticulum; Le/Lyso, late endosomes/lysosomes; Mito, mitochondria. (TIF) [file ppat.1006159.s021.tif]

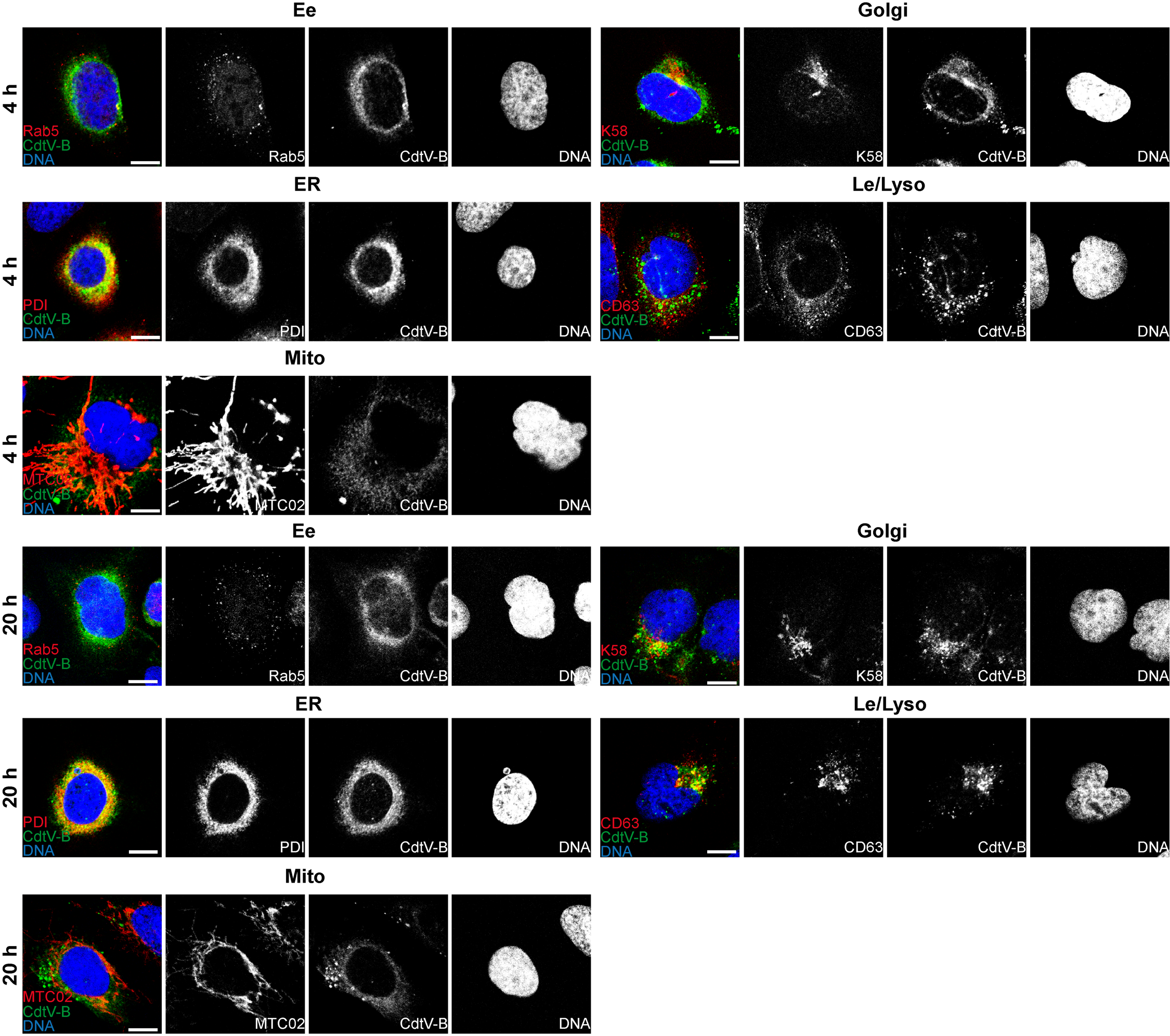

Supplement: S22 Fig — Scale bars are 10 μm. Ee, early endosomes; ER, endoplasmic reticulum; Le/Lyso, late endosomes/lysosomes; Mito, mitochondria. (TIF) [file ppat.1006159.s022.tif]

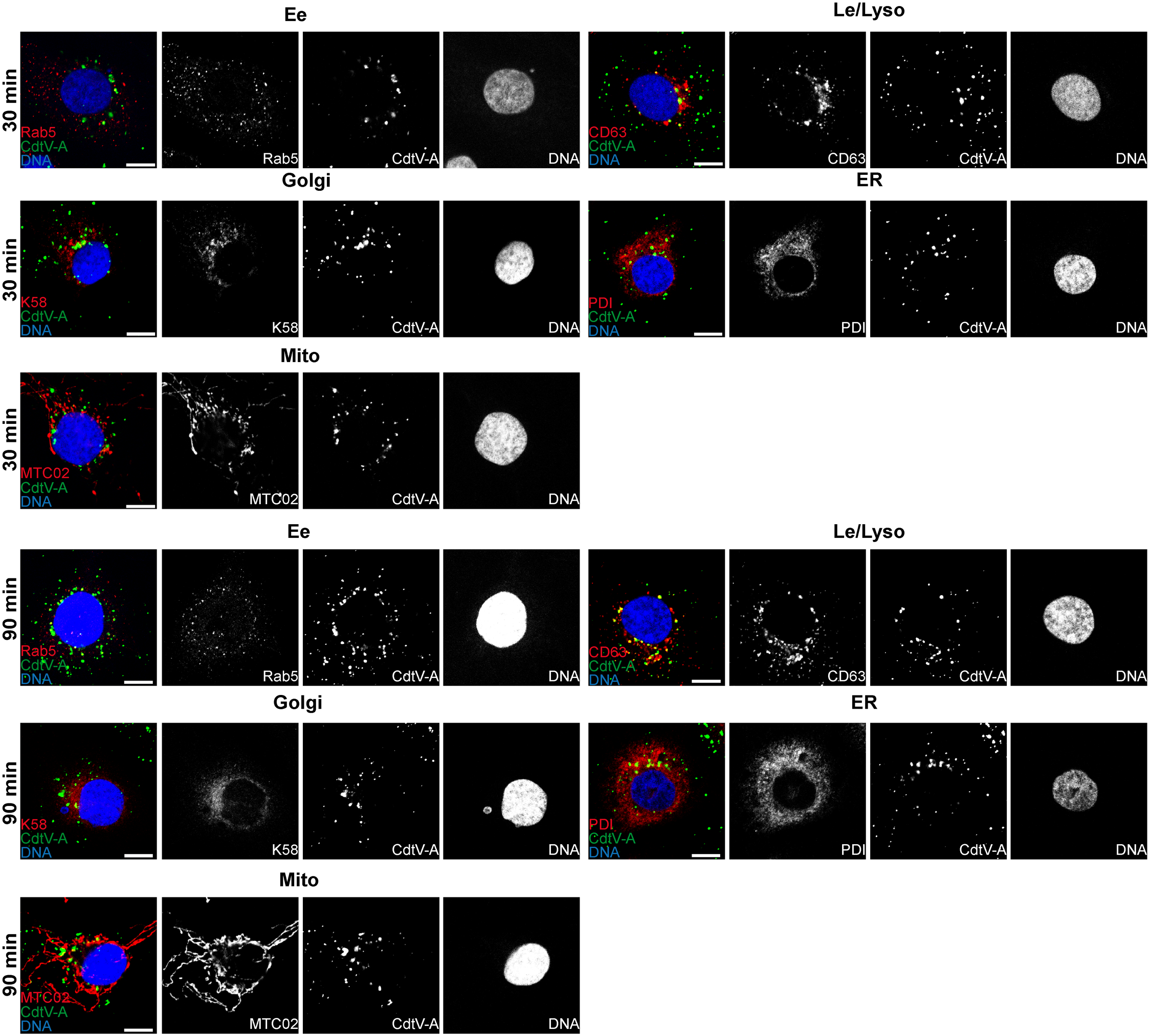

Supplement: S23 Fig — Scale bars are 10 μm. Ee, early endosomes; Le/Lyso, late endosomes/lysosomes; ER, endoplasmic reticulum; Mito, mitochondria. (TIF) [file ppat.1006159.s023.tif]

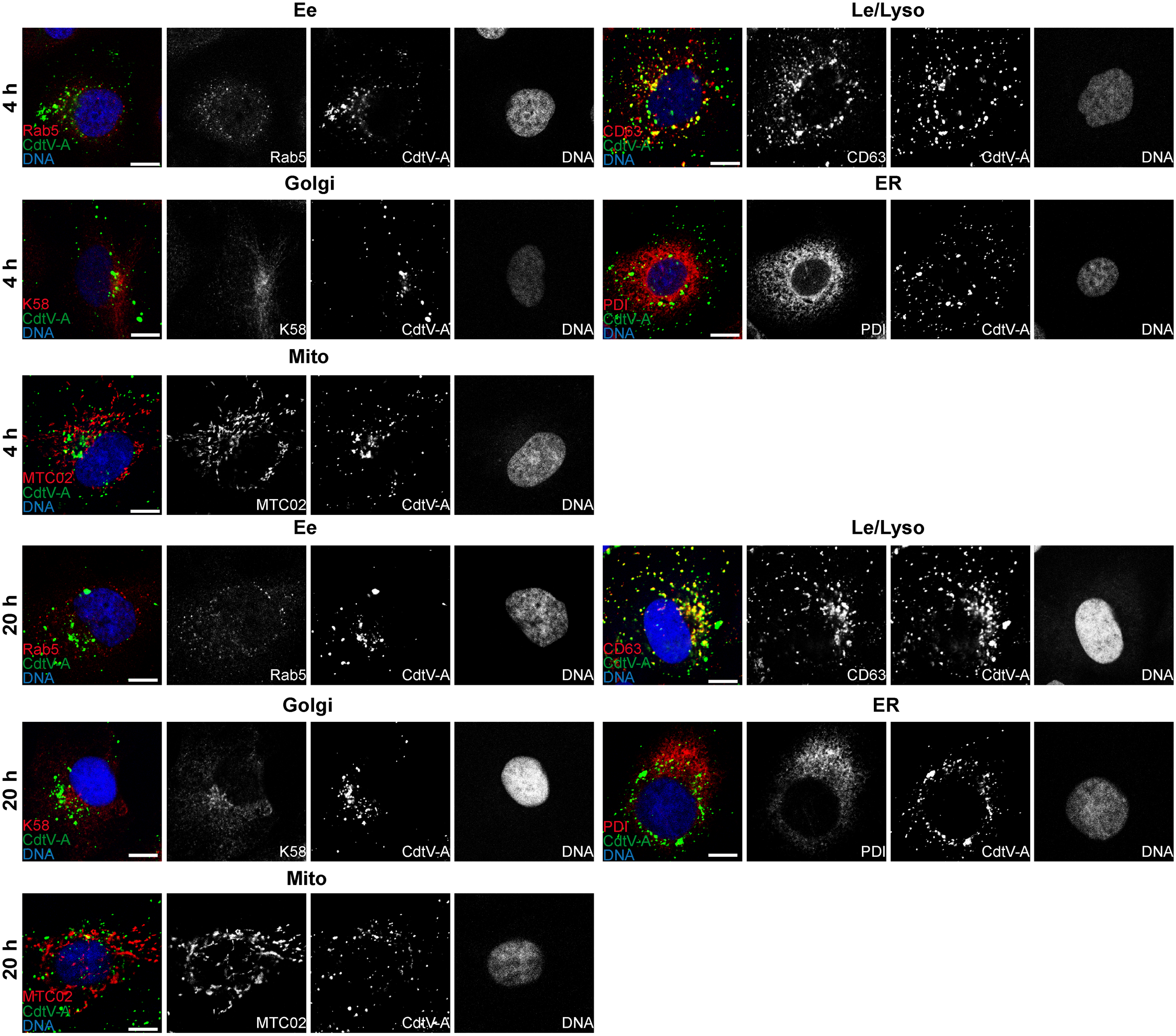

Supplement: S24 Fig — Scale bars are 10 μm. Ee, early endosomes; Le/Lyso, late endosomes/lysosomes; ER, endoplasmic reticulum; Mito, mitochondria. (TIF) [file ppat.1006159.s024.tif]

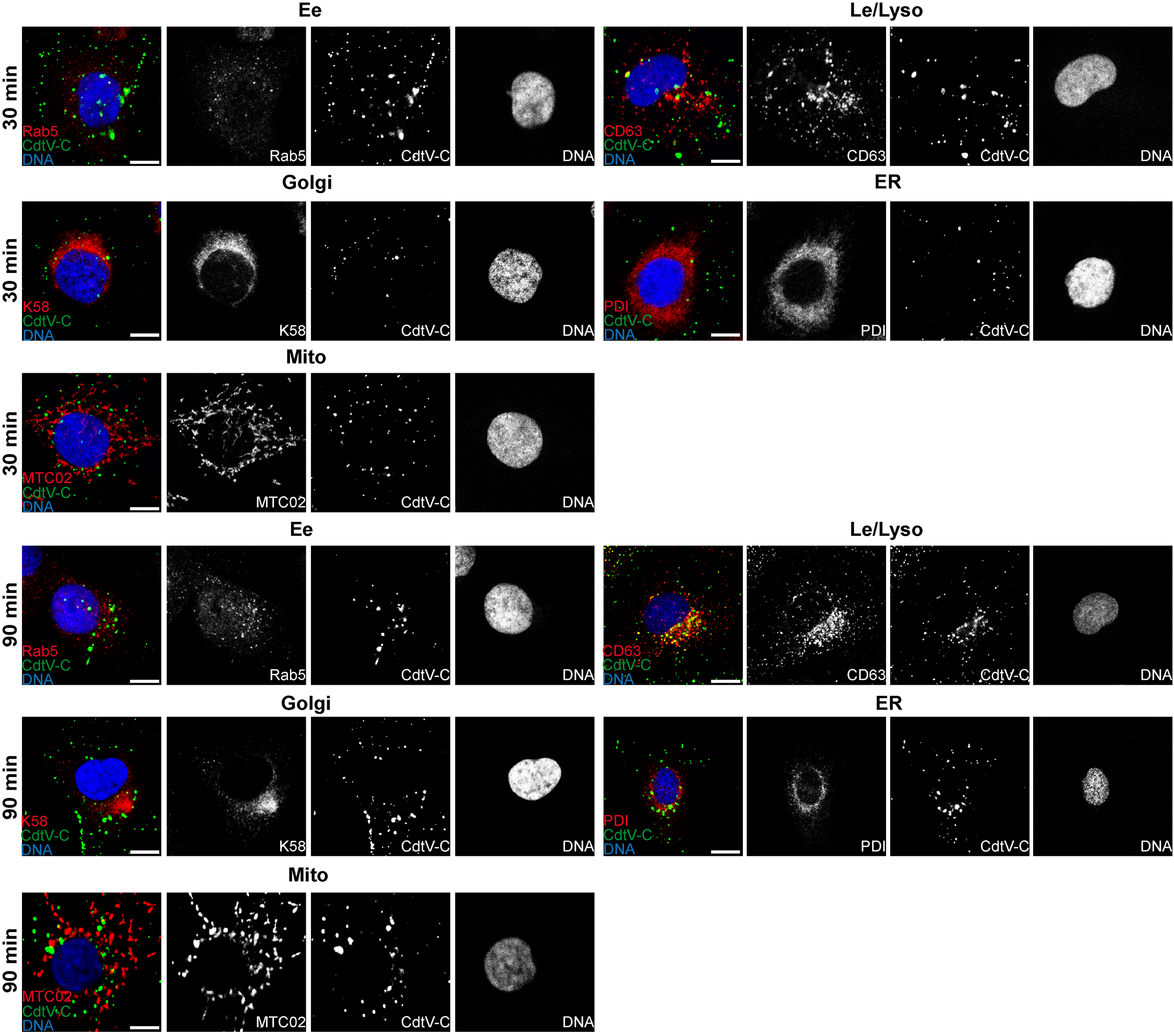

Supplement: S25 Fig — Scale bars are 10 μm. Ee, early endosomes; Le/Lyso, late endosomes/lysosomes; ER, endoplasmic reticulum; Mito, mitochondria. (TIF) [file ppat.1006159.s025.tif]

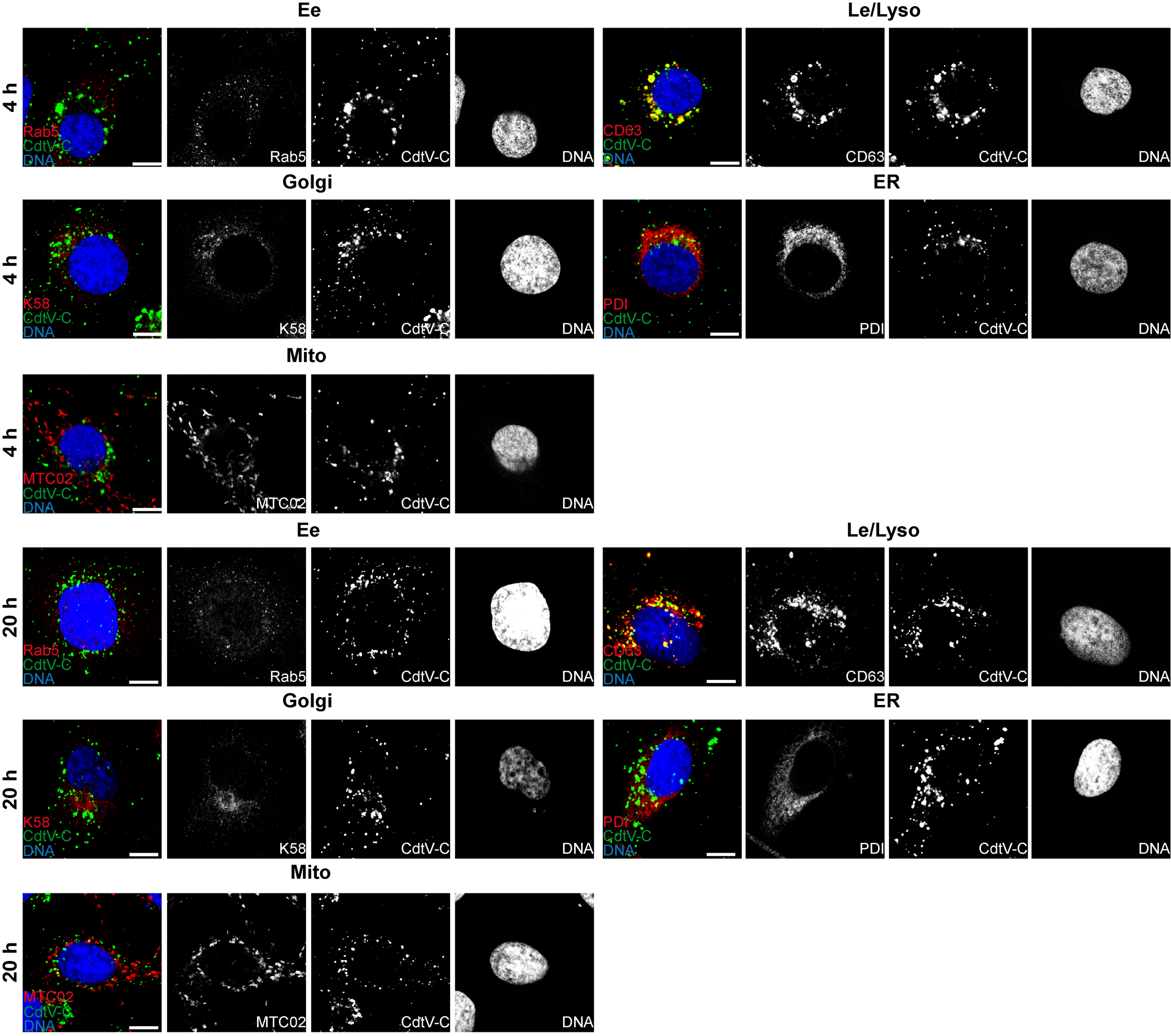

Supplement: S26 Fig — Scale bars are 10 μm. Ee, early endosomes; Le/Lyso, late endosomes/lysosomes; ER, endoplasmic reticulum; Mito, mitochondria. (TIF) [file ppat.1006159.s026.tif]

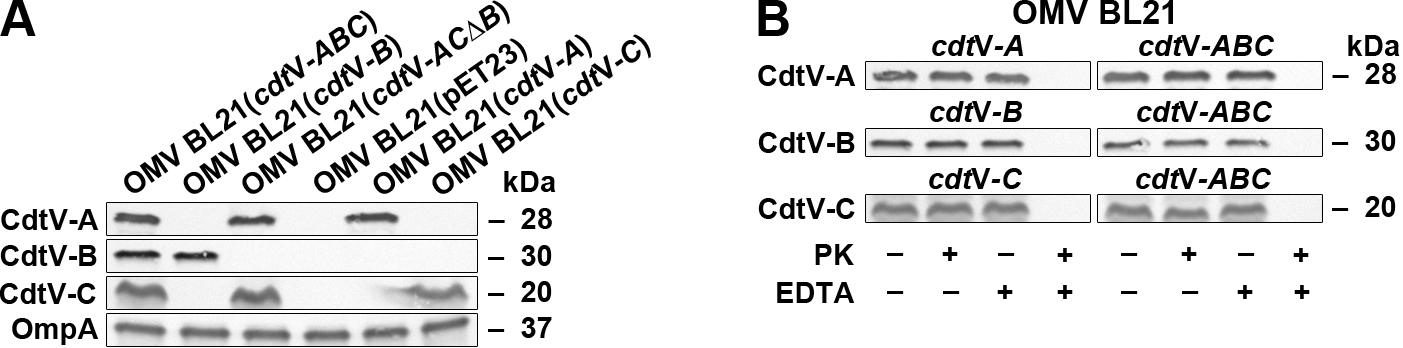

Supplement: S27 Fig — (A) Immunoblot analyses of OMVs from the indicated strains with anti-CdtV-A, anti-CdtV-B, and anti-CdtV-C antibodies. OMVs from BL21(pET23) (vector control) served as a negative control. OmpA is an OMV marker. (B) Intravesicular localization of the recombinant CdtV subunit proteins demonstrated by the proteinase K (PK) assay. PK-untreated (PK-) or PK-treated (PK+) OMVs from the indicated strains, either intact (EDTA-) or lysed with 0.1 M EDTA (EDTA+), were separated by SDS-PAGE and analyzed by immunoblot with the indicated antibodies. BL21(cdtV-ABC) OMVs carrying CdtV holotoxin were used as a control. (TIF) [file ppat.1006159.s027.tif]

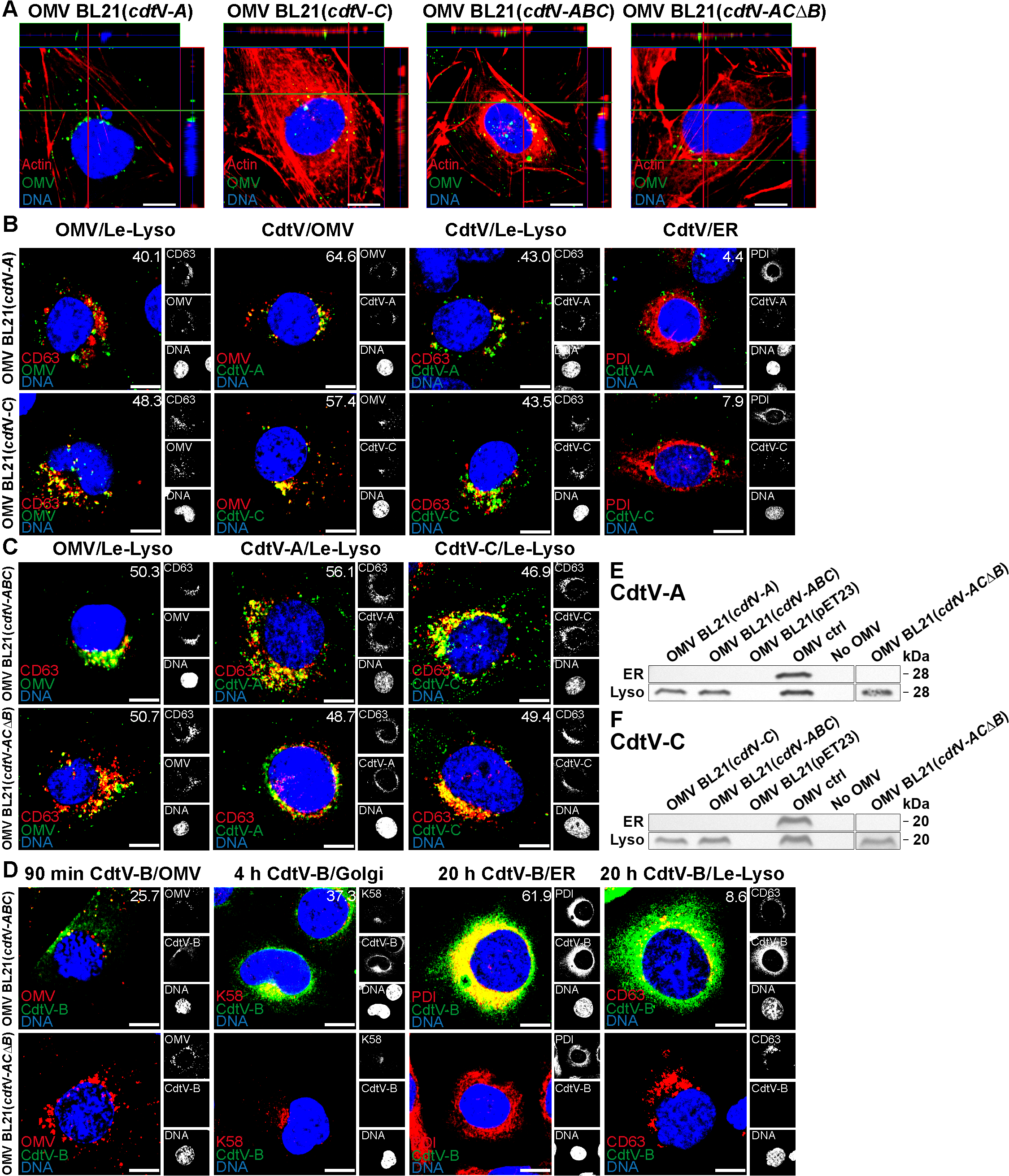

Supplement: S28 Fig — HBMEC were preincubated (30 min, 4°C) with OMVs from the indicated recombinant strains and postincubated at 37°C for 90 min to 20 h. (A) OMV uptake was determined by CLSM after 4 h. Green, OMVs; red, actin; blue, nuclei. Confocal Z-stack projections are included at upper/right sides. Crosshairs show the position of the xy and yz planes. Scale bars are 10 μm. (B, C) Colocalization of OMVs and OMV-delivered CdtV-A and CdtV-C expressed either singly (OMVs BL21(cdtV-A) and BL21(cdtV-C), respectively) or together (OMVs BL21(cdtV-ACΔB)) with the endoplasmic reticulum (ER) and late endosomes/lysosomes (Le-Lyso), and association of CdtV-A and CdtV-C with OMVs after 20 h determined by CLSM. The indicated single fluorescence channels are shown in the right panels and the merged images in the left panels (green, OMV or CdtV-A or CdtV-C, as indicated; red, OMVs or compartment-specific marker proteins, as indicated; blue, nuclei; yellow, colocalized green and red signals). The percentages of colocalizations of the respective signals (white numbers) were calculated with the BioImageXD6 tool (means of colocalizations from three different samples are shown). Scale bars are 10 μm. (D) Controls to CLSM data shown in Fig 11C. Separation of CdtV-B from OMVs, and detection of CdtV-B in the Golgi complex, endoplasmic reticulum (ER) and late endosomes/lysosomes (Le-Lyso) of HBMEC postincubated for the times indicated with OMVs BL21(cdtV-ABC) carrying CdtV holotoxin (positive control), or with CdtV-B lacking OMVs from cdtV-B deletion mutant BL21(cdtV-ACΔB) (negative control). The CLSM data were analyzed and are presented as given in B and C. Green, CdtV-B; red, OMV or compartment-specific marker proteins, as indicated; blue, nuclei; yellow, colocalized green and red signals (the percentages of colocalizations are shown by white numbers). (E, F) Immunoblot detection of CdtV-A (E) and CdtV-C (F) in isolated endoplasmic reticulum (ER) and lysosomes (Lyso) of HBMEC incubated with the indicated [file ppat.1006159.s028.tif]

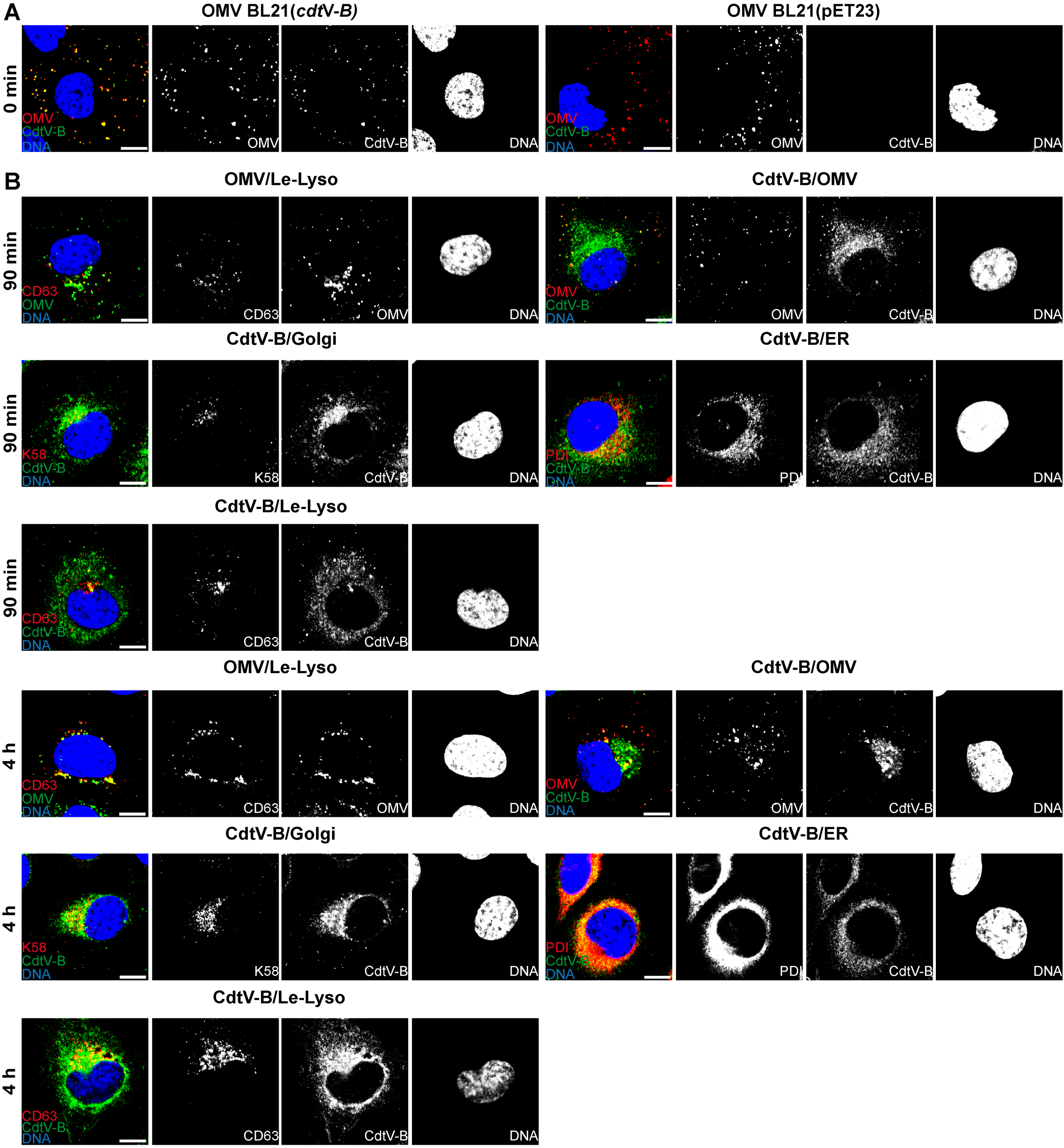

Supplement: S29 Fig — (A) Images shown in Fig 11B. (B) Images shown in Fig 11C, time points 90 min and 4 h. Scale bars are 10 μm. Le-Lyso, late endosomes/lysosomes; ER, endoplasmic reticulum. (TIF) [file ppat.1006159.s029.tif]

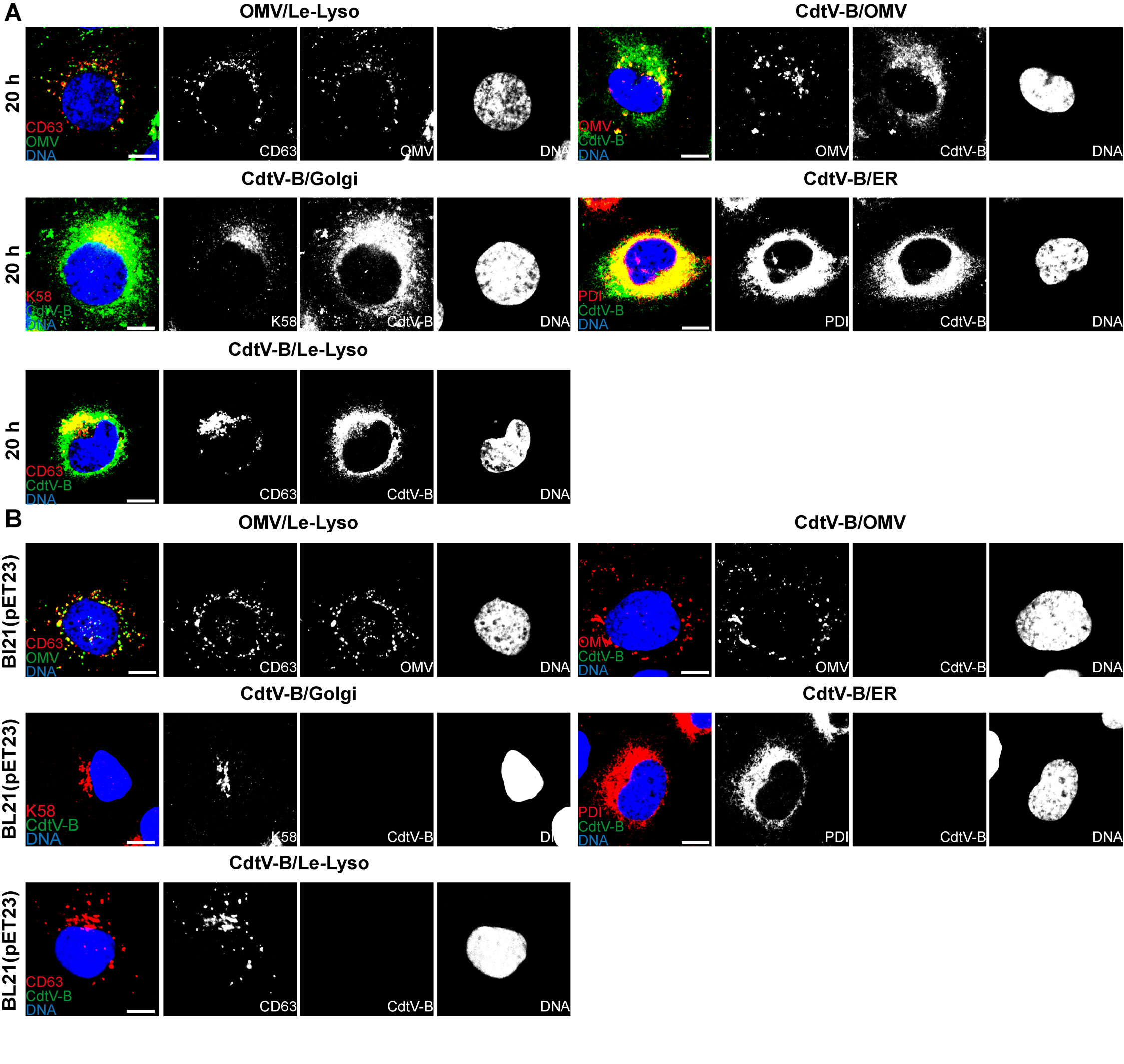

Supplement: S30 Fig — (A) Images shown in Fig 11C, time point 20 h. (B) Images shown in Fig 11D. Scale bars are 10 μm. Le-Lyso, late endosomes/lysosomes; ER, endoplasmic reticulum. (TIF) [file ppat.1006159.s030.tif]

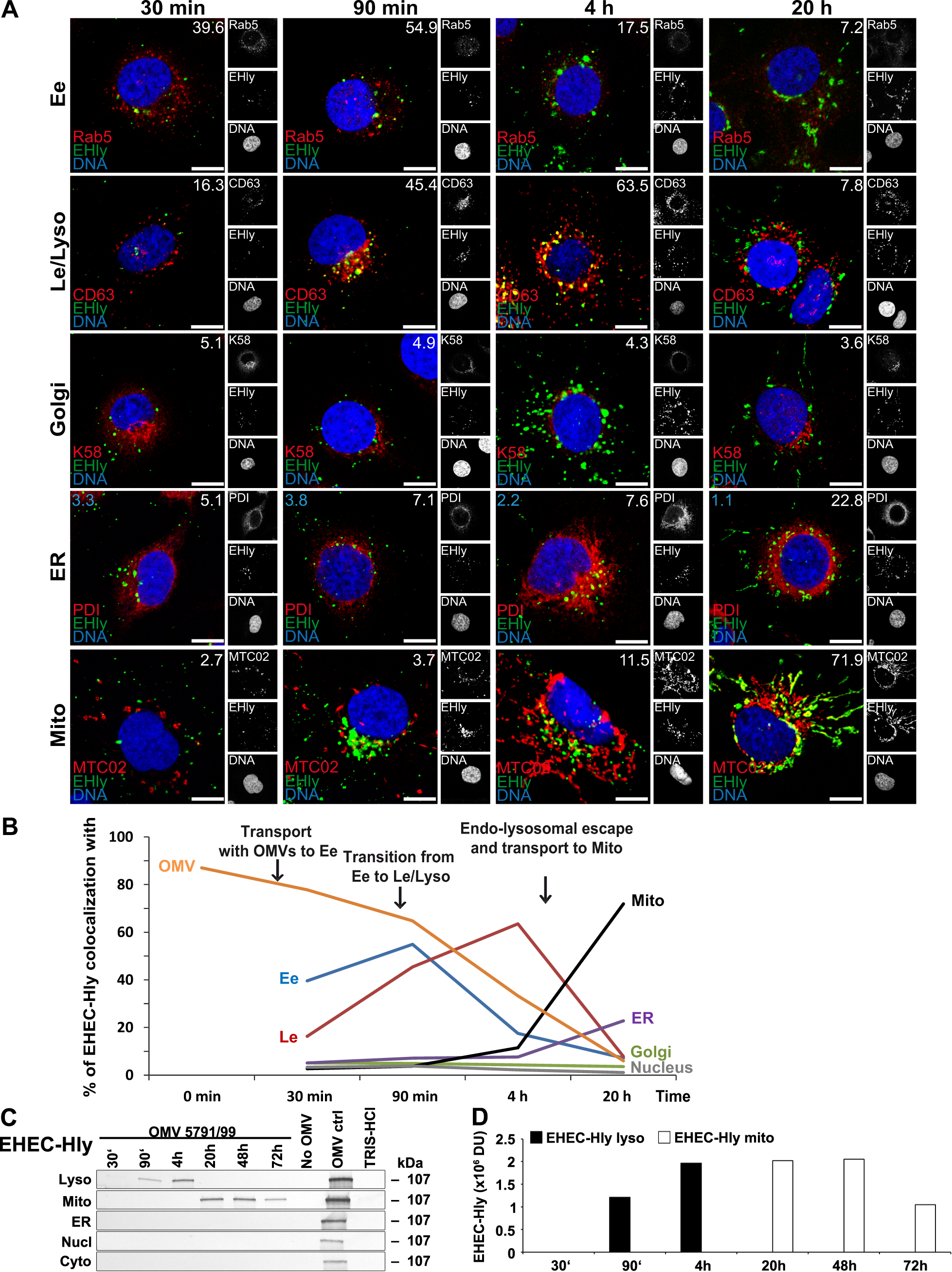

Supplement: S31 Fig — (A) CLSM of HBMEC preincubated with OMVs 5791/99 for 30 min at 4°C, and postincubated at 37°C for the times indicated. The indicated single fluorescence channels are shown in the right panels and the merged images in the left panels (green, EHEC-Hly (EHly); red, compartment-specific marker proteins; blue, nuclei; yellow, colocalized green and red signals). The percentages of EHEC-Hly colocalizations with compartment-specific marker proteins (white numbers) and with nucleus (blue numbers in panels ER) were calculated with the BioImageXD6 tool. Scale bars are 10 μm. (B) Graphical summary of EHEC-Hly colocalizations with subcellular compartments based on CLSM data shown in A, and with OMVs (based on data shown in Fig 3A). (Means of colocalizations from at least five different samples are shown in A and B; for standard deviations and significance analysis see S8G Fig). (C) Immunoblot detection of EHEC-Hly in isolated subcellular fractions of HBMEC which were incubated for the times indicated with 5791/99 OMVs, or for 72 h without OMVs or with TRIS-HCl OMV buffer (negative controls); 5791/99 OMVs without cells were a positive control. (D) Densitometric quantification of EHEC-Hly signals shown in C. Abbreviations: Ee, early endosomes; Le/Lyso, late endosomes/lysosomes; ER, endoplasmic reticulum; Mito, mitochondria; Nucl, nucleus; Cyto, cytoplasm. (TIF) [file ppat.1006159.s031.tif]

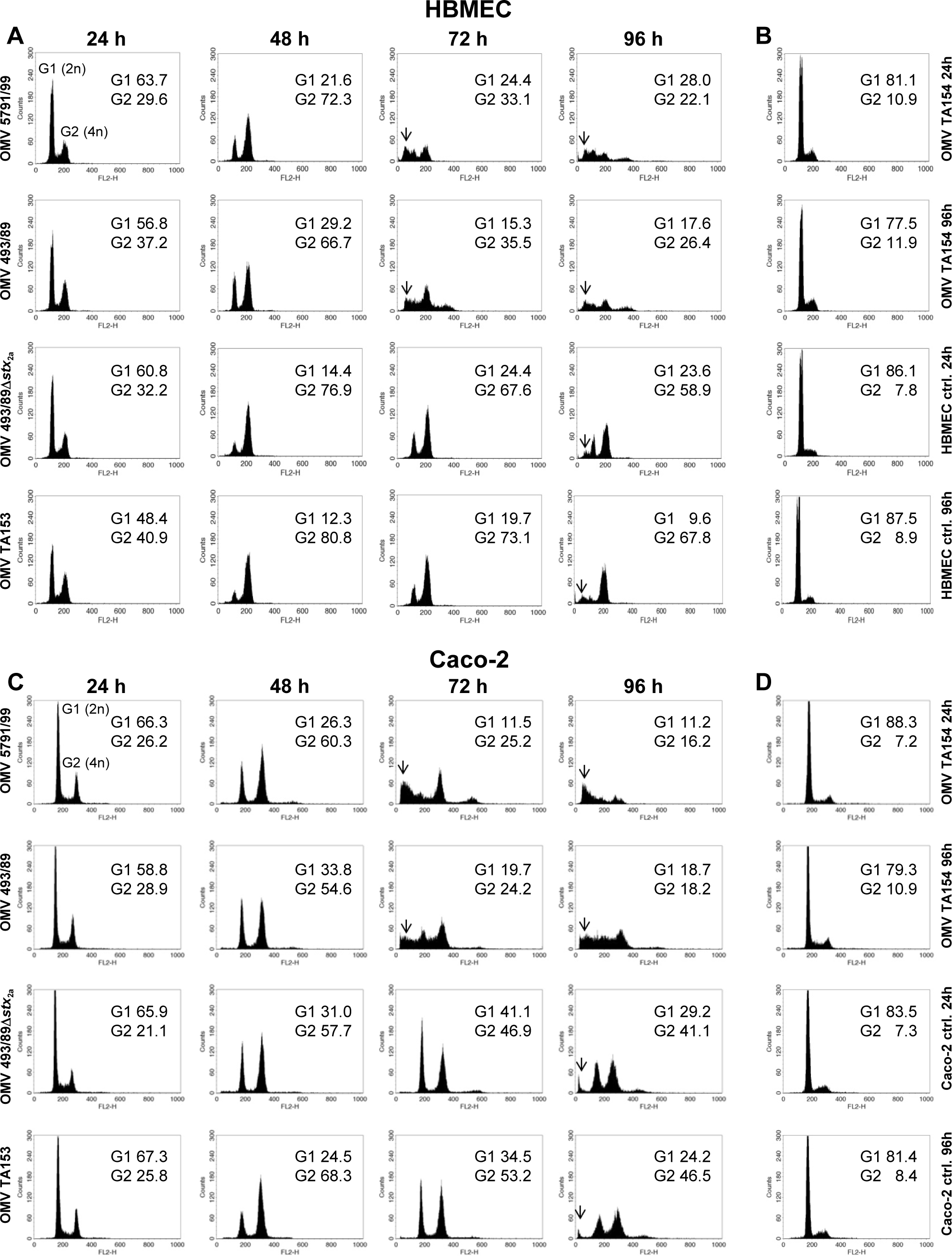

Supplement: S32 Fig — Flow cytometry histograms of (A) HBMEC and (C) Caco-2 cells treated for 24 h to 96 h with O157 OMVs or OMVs from CdtV-positive control strain TA153. (B) HBMEC and (D) Caco-2 cells treated for 24 h or 96 h with CdtV-negative OMVs from strain TA154 (vector control) or left untreated (negative controls). Positions of the G1 (2n DNA) and G2 (4n DNA) peaks are indicated in the first histograms in A and C. The proportions (%) of cells in G1 and G2 cell cycle phase, respectively, are shown in all histograms. Arrows depict sub-G1 populations (apoptotic cells). (TIF) [file ppat.1006159.s032.tif]

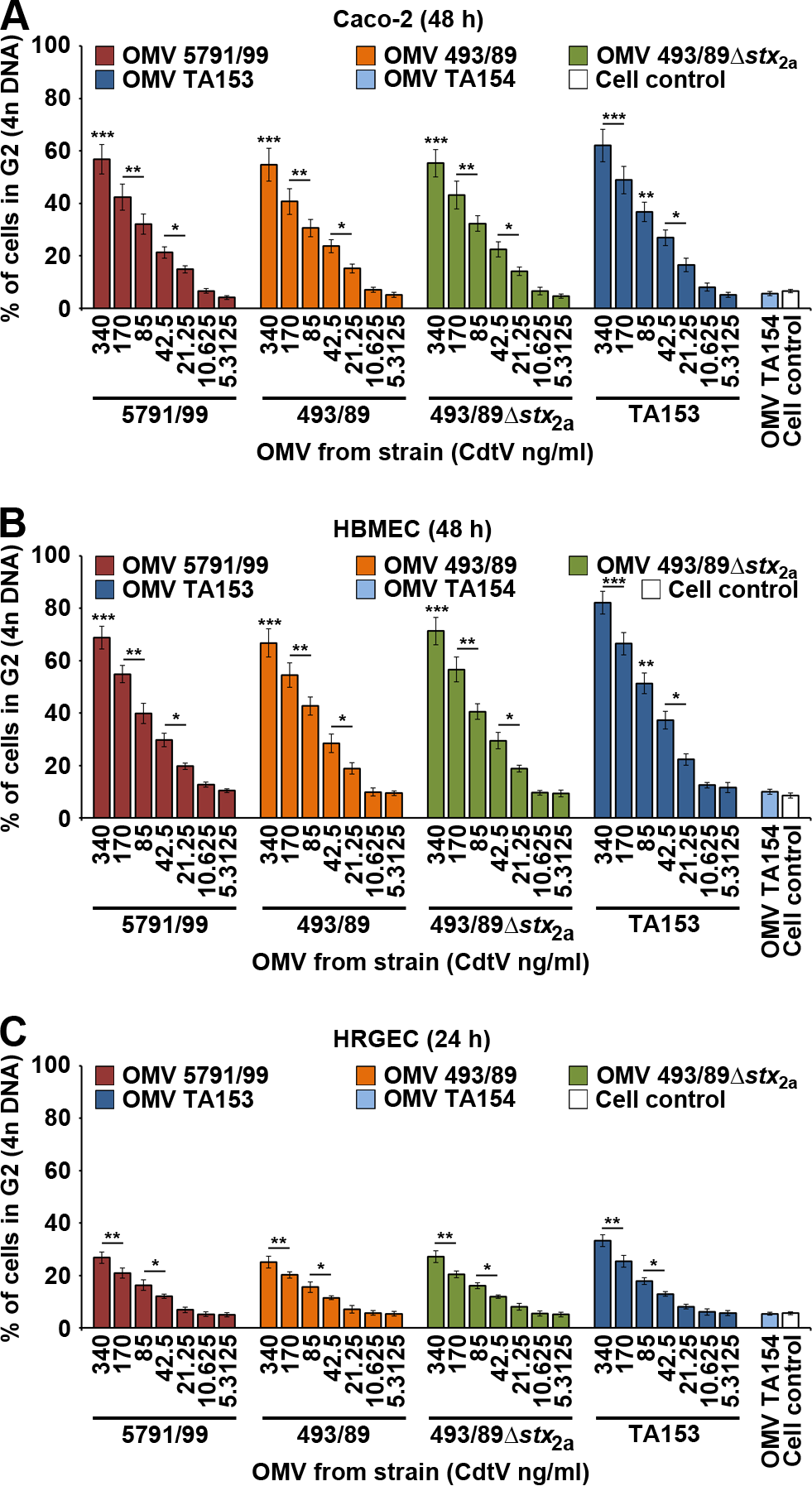

Supplement: S33 Fig — (A) Caco-2 cells, (B) HBMEC, and (C) HRGEC were incubated for 24 h (HRGEC) or 48 h (Caco-2, HBMEC) with two-fold dilutions of O157 (5791/99, 493/89, 493/89Δstx2a) or TA153 OMVs containing the indicated amounts of CdtV. Proportions of cells in G2 arrest (4n DNA content) were determined by flow cytometry. Data are means ± standard deviations from three independent experiments. *p < 0.05, **p < 0.01 or ***p < 0.001 (one-way ANOVA) for G2 arrest caused by the indicated CdtV doses compared to untreated cells (cell control). (TIF) [file ppat.1006159.s033.tif]

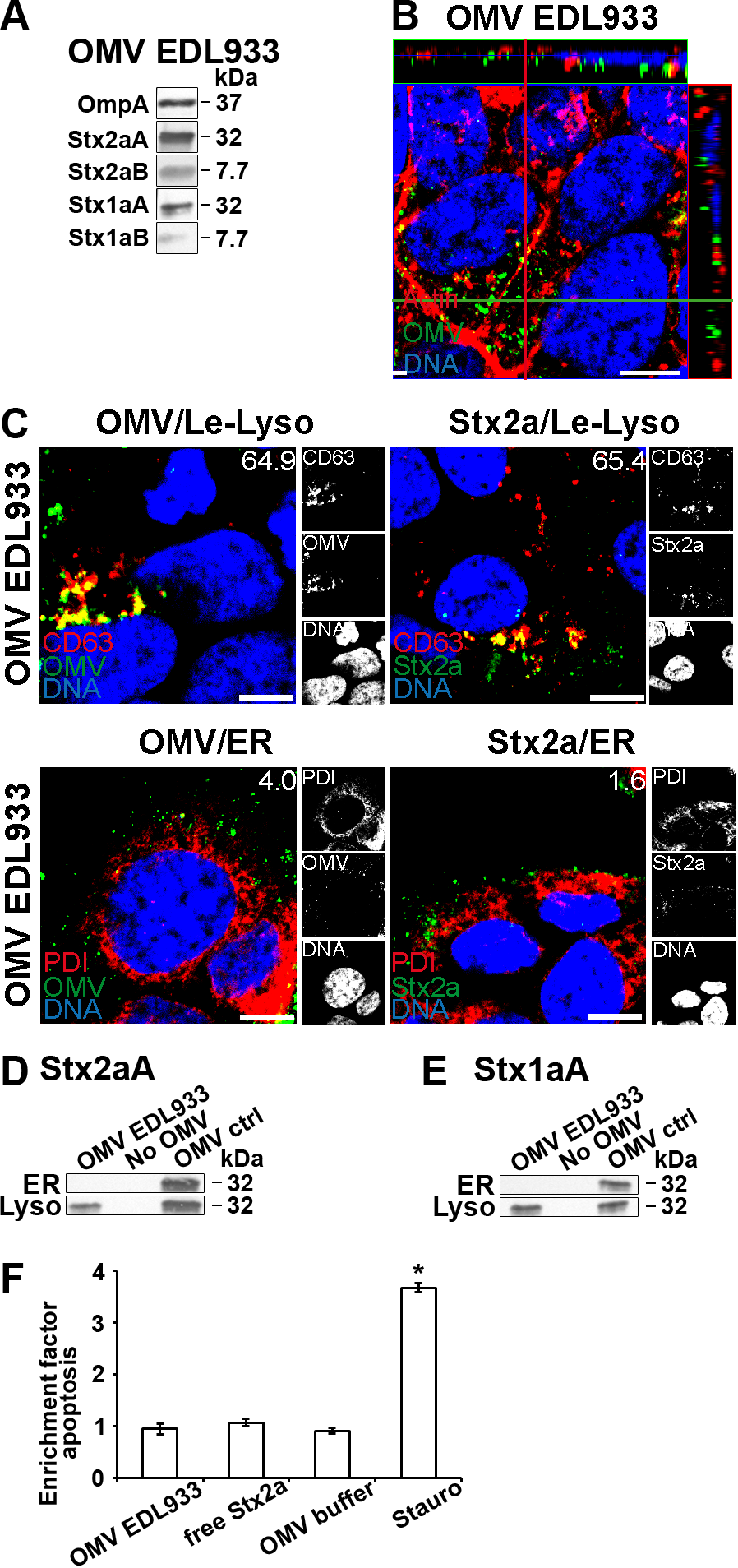

Supplement: S34 Fig — (A) Presence of Stx2a and Stx1a in EDL933 OMVs as detected by immunoblot. (B) EDL933 OMVs are internalized by Gb3-negative DLD-1 cells after 4 h of incubation as determined by CLSM. Green, OMVs; red, actin; blue, nuclei. Confocal Z-stack projections are included at upper/right sides. Crosshairs show the position of the xy and yz planes. (C) Colocalizations of EDL933 OMVs and OMV-delivered Stx2a with late endosomes/lysosomes (Le-Lyso) and the endoplasmic reticulum (ER) after 4 h of incubation of the OMVs with DLD-1 cells. The indicated single fluorescence channels are shown in the right panels and the merged images in the left panels (green, Stx2a or OMVs, as indicated; red, compartment-specific marker proteins, as indicated; blue, nuclei; yellow, colocalized green and red signals). The percentages of colocalization of the respective signals (white numbers) were calculated with the BioImageXD6 tool (means of colocalizations from three different samples are shown). Scale bars in B and C are 10 μm. (D, E) Immunoblot detection of Stx2a (D) and Stx1a (E) in isolated endoplasmic reticulum (ER) and lysosomal (Lyso) fractions of DLD-1 cells which were incubated for 4 h with EDL933 OMVs or left untreated (no OMV) (negative control). EDL933 OMVs without cells were a positive control (OMV ctrl). (F) Apoptosis caused in DLD-1 cells by the indicated samples after 96 h of incubation as determined by Cell Death Detection ELISA. Enrichment factors were calculated by dividing OD405 absorbance values of sample-treated cells with those of untreated cells. Staurosporine (1 μM) was a positive control and OMV buffer and free Stx2a which is not internalized by DLD-1 cells (Fig 6E) negative controls. *p < 0.05, apoptosis significantly higher than that caused by OMV buffer (one-way ANOVA). (TIF) [file ppat.1006159.s034.tif]

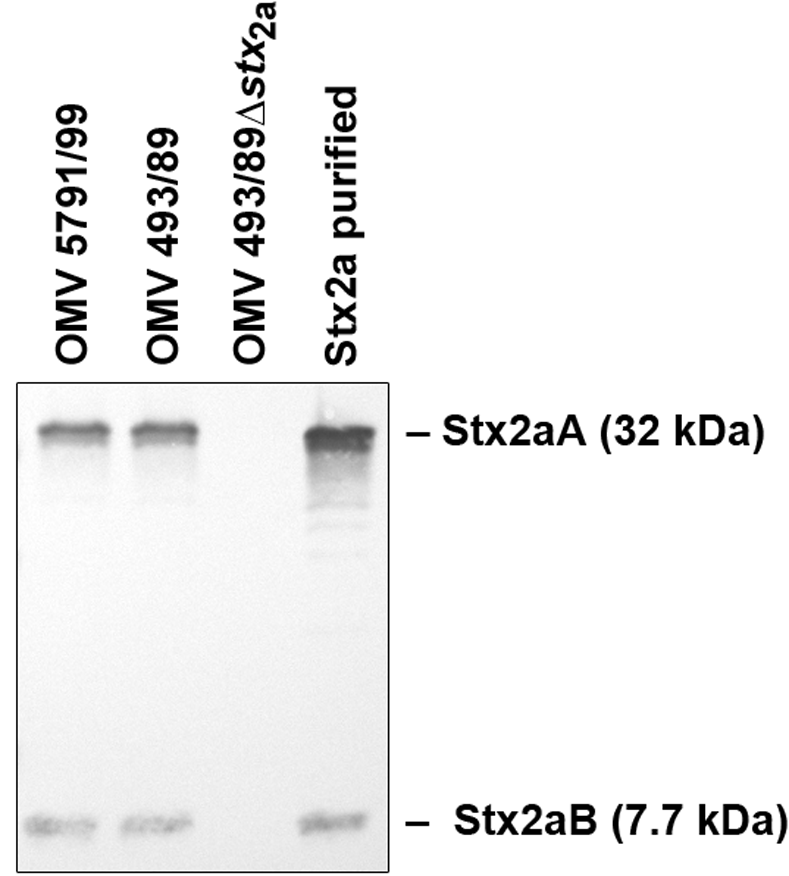

Supplement: S35 Fig — Samples were separated by SDS-PAGE and subjected to immunoblot with rabbit anti-Stx2a antibody (He et al., 2013). Sizes of Stx2a A and B subunits are indicated on the right side. (TIF) [file ppat.1006159.s035.tif]

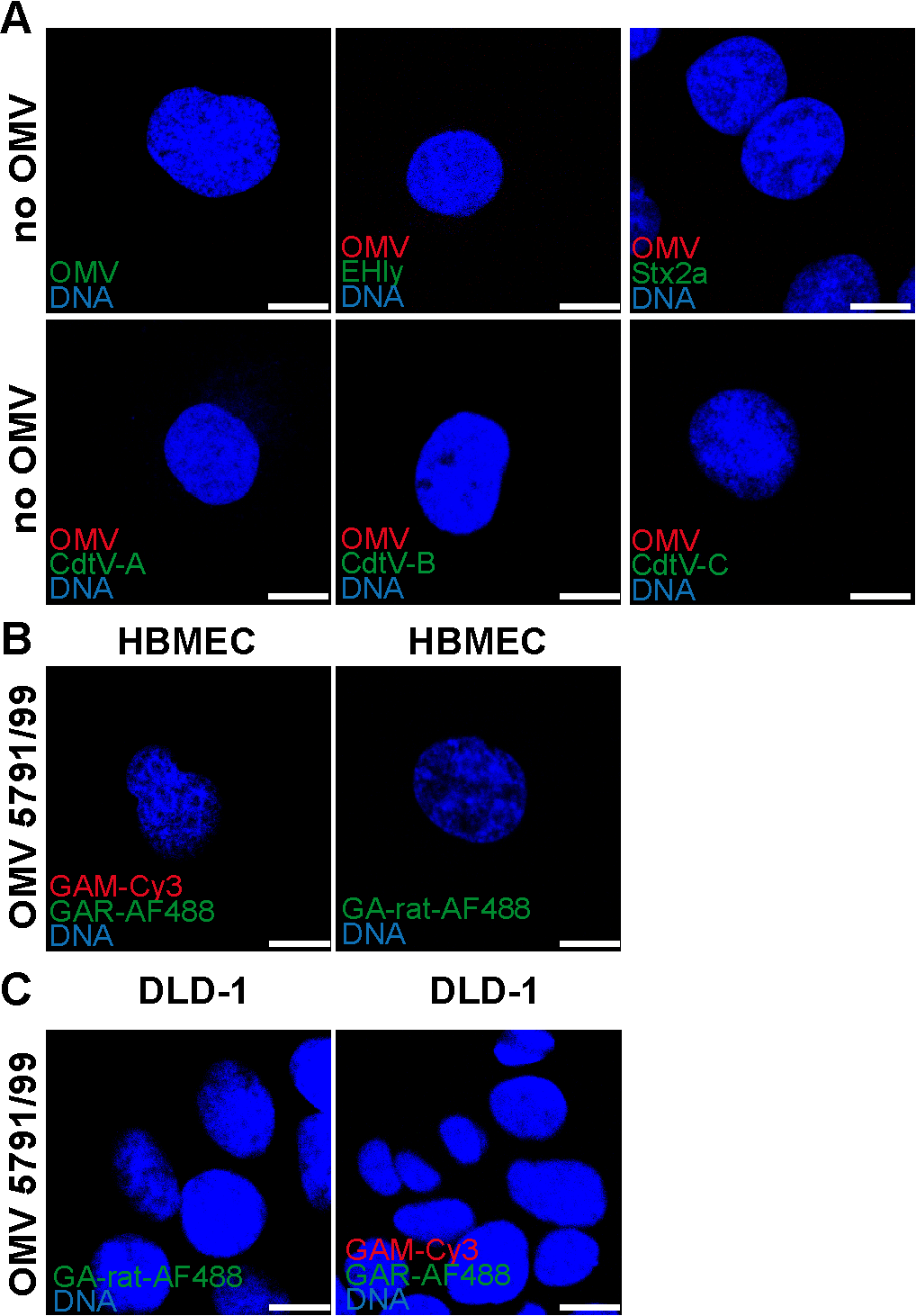

Supplement: S36 Fig — (A) HBMEC were incubated for 20 h with OMV buffer (20 mM TRIS-HCl) instead of OMVs. Cells were stained with rabbit anti-E. coli O157 LPS antibody and Alexa Fluor 488-conjugated goat anti-rabbit IgG or with mouse anti-E. coli O157 antibody and Cy3-conjugated goat anti-mouse IgG to detect OMVs. To detect virulence proteins, cells were stained with anti-Stx2a, anti-CdtV-A, -B, -C, or anti-EHEC-Hly rabbit antibodies and Alexa Fluor 488-conjugated goat anti-rabbit IgG. (B) HBMEC were incubated with 5791/99 OMVs for 20 h and stained with Cy3-conjugated goat anti-mouse IgG and with Alexa Fluor 488-conjugated goat anti-rabbit IgG, or with Alexa Fluor 488-conjugated goat anti-rat IgM in the absence of first antibodies. (C) DLD-1 cells were incubated for 4 h with 5791/99 OMVs and stained with Alexa Fluor 488-conjugated goat anti-rat IgM or with Cy3-conjugated goat anti-mouse IgG and Alexa Fluor 488-conjugated goat anti-rabbit IgG in the absence of first antibodies. Nuclei in all panels were stained with DRAQ5. Scale bars are 10 μm. (TIF) [file ppat.1006159.s036.tif]
