# Supplementary material for: Host cell interactions of outer membrane vesicle-associated virulence factors of enterohemorrhagic Escherichia coli O157: Intracellular delivery, trafficking and mechanisms of cell injury
Source: PLoS Pathog. 2017 Feb 3;13(2):e1006159. doi: 10.1371/journal.ppat.1006159 (PMC5310930; doi:10.1371/journal.ppat.1006159)
Supplement: S1 Table — (PDF) [file ppat.1006159.s037.pdf]

S1 Table. Wild-type and recombinant *E. coli* strains used in this study

Strain designation (reference or source)	Strain description[a]	Clinical diagnosis or source	Relevant virulence genes	Phenotypes[b]			
				Stx titer[c]	CdtV titer[d]	EHEC -Hly[e]	EspPα[f]
5791/99 (Friedrich et al., 2006)	O157:H7 (NSF)	HUS	*stx*$_{2a}$ *cdt*V-*ABC* EHEC-*hlyA* *espP*α *fliC*$_{H7}$	1024	1:8	+	+
493/89 (Karch et al., 1993	O157:H^{-} (SF)	HUS	*stx*$_{2a}$ *cdt*V-*ABC* EHEC-*hlyA* *fliC*$_{H7}$	512	1:8	-	n.t.
493/89Δ*stx*$_{2a}$ (this study)	O157:H^{-} (SF)	493/89 that lost *stx*$_{2a}$ during laboratory passages	*cdt*V-*ABC* EHEC-*hlyA* *fliC*$_{H7}$	<2	1:8	-	n.t.
EDL933 (Strockbine et al., 1986; Schmidt et al., 1995; Brockmeyer et al., 2007)	O157:H7 (NSF)	Hamburger meat	*stx*$_{1a}$, *stx*$_{2a}$ EHEC-*hlyA* *espP*α *fliC*$_{H7}$	1024	<2	+	+
TA153 (TA[g])	*E. coli* MC1061/SuperCos I *cdt*V-*ABC*$_{493/89}$ (AJ508930.1)	Laboratory strain	*cdt*V-*ABC*	<2	1:16	-	n.t.
TA154 (TA[g])	*E. coli* MC1061/SuperCos I	Laboratory strain	none	<2	<2	-	n.t.
BL21(*cdt*V-*ABC*) (this study)	*E. coli* BL21(DE3)/pET23b(+) *cdt*V-*ABC*$_{493/89}$	Laboratory strain	*cdt*V-*ABC*	<2	1:8	-	n.t.
BL21(*cdt*V-*A*) (this study)	*E. coli* BL21(DE3)/pET23b(+) *cdt*V-*A*$_{493/89}$	Laboratory strain	*cdt*V-*A*	<2	<2	-	n.t.
BL21(*cdt*V-*B*) (this study)	*E. coli* BL21(DE3)/pET23b(+) *cdt*V-*B*$_{493/89}$	Laboratory strain	*cdt*V-*B*	<2	1:4	-	n.t.
BL21(*cdt*V-*C*) (this study)	*E. coli* BL21(DE3)/pET23b(+) *cdt*V-*C*$_{493/89}$	Laboratory strain	*cdt*V-*C*	<2	<2	-	n.t.
BL21(*cdt*V-*ACΔB*) (this study)	*E. coli* BL21(DE3)/pET23b(+) *cdt*V-*ACΔB*$_{493/89}$	Laboratory strain	*cdt*V-*AC*	<2	<2	-	n.t.
BL21(pET23) (this study)	*E. coli* BL21(DE3)/pET23b(+)	Laboratory strain	none	<2	<2	-	n.t.

[a] NSF, non-sorbitol-fermenting; SF, sorbitol-fermenting; H^{-}, non-motile (although they possess flagellin-encoding *fliC*$_{H7}$, SF O157 strains do not express flagellin due to a deletion in the transcriptional regulator *flhC* that is required for transcription of genes involved in flagellum biosynthesis) (Karch et al., 2005).
[b] Stx, Shiga toxin; CdtV, cytolethal distending toxin V; EHEC-Hly, EHEC hemolysin; EspPα, serine protease EspPα.

[c] Determined by Vero cell assay (Bauwens et al., 2011); the highest dilution of the bacterial supernatant which killed 50% Vero cells after 3 days.
[d] Determined by Chinese hamster ovary cell (CHO) assay (Janka et al., 2003); the highest dilution of the bacterial supernatant which caused distension in 50% CHO cells after 3 days.
[e] Tested on enterohemolysin agar (Bielaszewska et al., 2013); +, presence of enterohemolytic phenotype; -, no enterohemolytic phenotype (EHEC-*hlyA* is usually not expressed by SF EHEC O157:H^-) (Karch et al., 2005).
[f] Determined by the ability of the bacterial supernatant to cleave the para-nitroaniline-conjugated oligopeptide substrate alanine-alanine-proline-leucine (Brockmeyer et al., 2007); +, the substrate is cleaved; n.t., not tested.
[g] Provided by Thomas Aldick (Institute of Hygiene, University of Münster).
