# Supplementary material for: Host cell interactions of outer membrane vesicle-associated virulence factors of enterohemorrhagic Escherichia coli O157: Intracellular delivery, trafficking and mechanisms of cell injury
Source: PLoS Pathog. 2017 Feb 3;13(2):e1006159. doi: 10.1371/journal.ppat.1006159 (PMC5310930; doi:10.1371/journal.ppat.1006159)
Supplement: S2 Table — (PDF) [file ppat.1006159.s038.pdf]

S2 Table. Proteins identified in OMVs from NSF *E. coli* O157:H7 strain 5791/99 using nano-LC-MS/MS

Accession no.	Protein description and subcellular localization
	**Outer membrane (n = 12)**
gi|15830295	Outer membrane protein A [*Escherichia coli* O157:H7 str. Sakai]
gi|15832358	Outer membrane protein C [*Escherichia coli* O157:H7 str. Sakai]
gi|15831010	Outer membrane protein W [*Escherichia coli* O157:H7 str. Sakai]
gi|15830146	Outer membrane protein X [*Escherichia coli* O157:H7 str. Sakai]
gi|15830917	Outer membrane protease OmpT [*Escherichia coli* O157:H7 str. Sakai]
gi|15832196	Outer membrane protein Lom [*Escherichia coli* O157:H7 str. Sakai]
gi|71162373	Outer membrane lipoprotein SlyB [*Escherichia coli* O157:H7]
gi|13363851	Outer membrane protein Slp [*Escherichia coli* O157:H7 str. Sakai]
gi|13363396	Outer membrane channel TolC [*Escherichia coli* O157:H7 str. Sakai]
gi|13362697	Long-chain fatty acid transport protein FadL [*Escherichia coli* O157:H7 str. Sakai]
gi|15830030	Peptidoglycan-associated outer membrane lipoprotein [*Escherichia coli* O157:H7 str. Sakai]
gi|412970523	Small membrane protein A [*Escherichia coli*]
	**Periplasm (n = 5)**
**gi|15830459**	**Shiga toxin 2 subunit A [*Escherichia coli* O157:H7 str. Sakai]**
**gi|13360666**	**Shiga toxin 2 subunit B [*Escherichia coli* O157:H7 str. Sakai]**
**gi|23574039**	**Cytolethal distending toxin B [*Escherichia coli* O157:H- str. 493/89]**[a]
gi|390666152	Cytochrome c-552 [*Escherichia coli* O157 FRIK1990]
gi|15834228	Major head subunit Mu-like prophage [*Escherichia coli* O157:H7 str. Sakai]
	**Cytoplasmic membrane (n =10)**
**gi|23574040**	**Cytolethal distending toxin C [*Escherichia coli* O157:H- str. 493/89]**[a]
gi|15830022	Cytochrome d terminal oxidase polypeptide subunit I [*Escherichia coli* O157:H7 str. Sakai]
gi|15833932	ATP synthase F0F1 subunit B [*Escherichia coli* O157:H7 str. Sakai]
gi|13361996	Mannose-specific PTS enzyme IID [*Escherichia coli* O157:H7 str. Sakai]
gi|83286919	Acriflavine resistance protein A precursor [*Escherichia coli* O157:H7]
gi|15830003	Succinate dehydrogenase iron-sulfur subunit [*Escherichia coli* O157:H7 str. Sakai]
gi|13359952	Putative protease maturation protein [*Escherichia coli* O157:H7 str. Sakai]
gi|25285105	NADH dehydrogenase I chain A [*Escherichia coli* (strain O157:H7 str. EDL933]
gi|15833360	Cytochrome d ubiquinol oxidase subunit III [*Escherichia coli* O157:H7 str. Sakai]
gi|15831351	Acid sensitivity protein [*Escherichia coli* O157:H7 str. Sakai]
	**Cytoplasm (n = 20)**
gi|15834378	Molecular chaperone GroEL [*Escherichia coli* O157:H7 str. Sakai]
gi|25283746	Hypothetical protein adhE [*Escherichia coli* O157:H7 str. EDL933]

gi|38704198	Tryptophanase [*Escherichia coli* O157:H7 str. Sakai]
gi|15829372	Pyruvate dehydrogenase subunit E1 [*Escherichia coli* O157:H7 str. Sakai]
gi|15833930	ATP synthase F0F1 subunit alpha [*Escherichia coli* O157:H7 str. Sakai]
gi|13364383	50S ribosomal subunit protein L1 [*Escherichia coli* O157:H7 str. Sakai]
gi|15834433	50S ribosomal protein L9 [*Escherichia coli* O157:H7 str. Sakai]
gi|13364384	50S ribosomal subunit protein L10 [*Escherichia coli* O157:H7 str. Sakai]
gi|15833415	30S ribosomal protein S4 [*Escherichia coli* O157:H7 str. Sakai]
gi|15833422	30S ribosomal protein S5 [*Escherichia coli* O157:H7 str. Sakai]
gi|15833446	30S ribosomal protein S7 [*Escherichia coli* O157:H7 str. Sakai]
gi|15834157	Elongation factor Tu [*Escherichia coli* O157:H7 str. Sakai]
gi|15833445	Elongation factor G [*Escherichia coli* O157:H7 str. Sakai]
gi|15834046	Glutamine synthetase [*Escherichia coli* O157:H7 str. Sakai]
gi|13360203	Citrate synthase [*Escherichia coli* O157:H7 str. Sakai]
gi|38704234	Aspartate ammonia-lyase [*Escherichia coli* O157:H7 str. Sakai]
gi|15834187	Isocitrate lyase [*Escherichia coli* O157:H7 str. Sakai]
gi|15830002	Succinate dehydrogenase flavoprotein subunit [*Escherichia coli* O157:H7 str. Sakai]
gi|15834165	DNA-directed RNA polymerase subunit beta [*Escherichia coli* O157:H7 str. Sakai]
gi|12515069	Unknown protein encoded within prophage CP-933O [*Escherichia coli* O157:H7 str. EDL933
**Extracellular (n = 6)**	
**gi|15831916**	**Flagellin [*Escherichia coli* O157:H7 str. Sakai]**
gi|15830714	Flagellar hook-associated protein FlgK [*Escherichia coli* O157:H7 str. Sakai]
gi|15830715	Flagellar hook-associated protein FlgL [*Escherichia coli* O157:H7 str. Sakai]
gi|15831917	Flagellar capping protein FliD [*Escherichia coli* O157:H7 str. Sakai]
**gi|4388764**	**Hemolysin [*Escherichia coli* O157:H7 str. EDL933] (EHEC-hemolysin)**
gi|209157354	Phage tail collar domain protein [*Escherichia coli* O157:H7 str. EC4115]
**Unknown localization (n = 6)**	
**gi|23574038**	**Cytolethal distending toxin A [*Escherichia coli* O157:H- str. 493/89]**[a]
gi|373245023	Major tail sheath protein [Escherichia coli 4_1_47FAA]
gi|209157292	Phage lysis timing protein LysB [*Escherichia coli* O157:H7 str. EC4115]
gi|83288109	Uncharacterized lipoprotein YifL [*Escherichia coli* O157:H7]
gi|12513293	Hypothetical protein Z0537 [*Escherichia coli* O157:H7 str. EDL933]
gi|15833743	Hypothetical protein ECs4489 [*Escherichia coli* O157:H7 str. Sakai]

Known virulence factors of *E. coli* O157:H7/H$^-$ are shown in bold.
[a] Cytolethal distending toxin A, B, and C are three components of CdtV holotoxin.
