# Supplementary material for: Host cell interactions of outer membrane vesicle-associated virulence factors of enterohemorrhagic Escherichia coli O157: Intracellular delivery, trafficking and mechanisms of cell injury
Source: PLoS Pathog. 2017 Feb 3;13(2):e1006159. doi: 10.1371/journal.ppat.1006159 (PMC5310930; doi:10.1371/journal.ppat.1006159)
Supplement: S3 Table — (PDF) [file ppat.1006159.s039.pdf]

S3 Table. Proteins identified in OMVs from SF *E. coli* O157:H[-] strains 493/89 and 493/89Δ*stx*$_{2a}$ using nano-LC-MS/MS

Accession no.	Protein description and subcellular localization	OMV 493/89	OMV 493/89Δ*stx*$_{2a}$
	**Outer membrane (n = 16)**		
gi|320642950	Outer membrane protein A [*Escherichia coli* O157:H- str. 493-89]	+[a]	+
gi|320646557	Outer membrane porin protein C [*Escherichia coli* O157:H- str. 493-89]	+	+
gi|320642923	Outer membrane protein F [*Escherichia coli* O157:H- str. 493-89]	+	+
gi|320642714	Outer membrane protein W [*Escherichia coli* O157:H- str. 493-89]	+	+
gi|320642803	Outer membrane protein X [*Escherichia coli* O157:H- str. 493-89]	+	+
gi|320643931	Putative outer membrane precursor Lom [*Escherichia coli* O157:H- str. 493-89]	+	+
gi|320647313	Outer membrane lipoprotein SlyB [*Escherichia coli* O157:H- str. 493-89]	+	+
gi|320646387	Long-chain fatty acid outer membrane transporter [*Escherichia coli* O157:H- str. 493-89]	+	+
gi|320643395	Peptidoglycan-associated outer membrane lipoprotein [*Escherichia coli* O157:H- str. 493-89]	+	+
gi|412970523	Small membrane protein A [*Escherichia coli*]	+	+
gi|320644161	Maltoporin [*Escherichia coli* O157:H- str. 493-89]	+	+
gi|320647348	Murein lipoprotein [*Escherichia coli* O157:H- str. 493-89]	+	+
gi|12518203	Outer membrane protein induced after carbon starvation [*Escherichia coli* O157:H7 str. EDL933]	+	+
gi|320643337	Acyl-CoA thioesterase [*Escherichia coli* O157:H- str. 493-89]	+	+
gi|12513752	Putative outer membrane protein of prophage CP-933K [*Escherichia coli* O157:H7 str. EDL933]	+	+
gi|320643783	Nucleoside-specific channel-forming protein Tsx [*Escherichia coli* O157:H- str. 493-89]	+	+
	**Periplasm (n = 6)**		
**gi|23320677**	**Shiga toxin 2 subunit A [*Escherichia coli* O157:H-] (strain 493/89)**	**+**	**-**
**gi|23320678**	**Shiga toxin 2 subunit B [*Escherichia coli* O157:H-] (strain 493/89)**	**+**	**-**
**gi|23574039**	**Cytolethal distending toxin B [*Escherichia coli* O157:H- str. 493/89]**[b]	**+**	**+**
gi|320643829	Putative lipoprotein [*Escherichia coli* O157:H- str. 493-89]	+	+
gi|320645598	Malate dehydrogenase [*Escherichia coli* O157:H- str. 493-89]	+	+
gi|320644754	Periplasmic chaperone [*Escherichia coli* O157:H- str. 493-89]	+	+

	**Cytoplasmic membrane (n = 10)**		
**gi|23574040**	**Cytolethal distending toxin C [*Escherichia coli* O157:H- str. 493/89]**[b]	+	+
gi|209776448	Cytochrome d terminal oxidase polypeptide subunit I [*Escherichia coli* O157 strain 493-89]	+	+
gi|320644904	F0F1 ATP synthase subunit B [*Escherichia coli* O157:H- str. 493-89]	+	+
gi|320644900	F0F1 ATP synthase subunit beta [*Escherichia coli* O157:H- str. 493-89]	+	+
gi|320644286	Fumarate reductase flavoprotein subunit [*Escherichia coli* O157:H- str. 493-89]	+	+
gi|320644279	Entericidin B membrane lipoprotein [*Escherichia coli* O157:H- str. 493-89]	+	+
gi|147531	Lipoprotein 28, partial [*Escherichia coli*]	+	-
gi|446314564	Membrane protein [Escherichia coli]	+	+
gi|377929587	Hypothetical protein ECDEC3F_1490 [*Escherichia coli* O157:H7 DEC3F]	+	+
gi|320645595	Hypothetical protein ECO9389_08217 [*Escherichia coli* O157:H- str. 493-89]	+	+
	**Cytoplasm (n = 25)**		
gi|320644273	Chaperonin GroEL [*Escherichia coli* O157:H- str. 493-89]	+	+
gi|25283746	Hypothetical protein adhE [*Escherichia coli* O157:H7 str. EDL933]	+	+
gi|320644876	Tryptophanase/L-cysteine desulfhydrase, PLP-dependent [*Escherichia coli* O157:H- str. 493-89]	+	+
gi|320647514	Pyruvate dehydrogenase subunit E1 [*Escherichia coli* O157:H- str. 493-89]	+	+
gi|320644903	F0F1 ATP synthase subunit delta [*Escherichia coli* O157:H- str. 493-89]	+	+
gi|13364383	50S ribosomal subunit protein L1 [*Escherichia coli* O157:H7 str. Sakai]	+	+
gi|320644469	50S ribosomal protein L10 [*Escherichia coli* O157:H- str. 493-89]	+	+
gi|320645106	30S ribosomal protein S5 [*Escherichia coli* O157:H- str. 493-89]	+	+
gi|320645130	30S ribosomal protein S7 [*Escherichia coli* O157:H- str. 493-89]	+	+
gi|320645128	Elongation factor Tu [*Escherichia coli* O157:H- str. 493-89]	+	+
gi|12513735	Putative protease encoded in prophage CP-933K [*Escherichia coli* O157:H7 str. EDL933]	+	+
gi|320647515	Pyruvate dehydrogenase dihydrolipoyltransacetylase [*Escherichia coli* O157:H- str. 493-89]	+	+
gi|320647516	Dihydrolipoamide dehydrogenase [*Escherichia coli* O157:H- str. 493-89]	+	-
gi|320642698	Bifunctional acetaldehyde-CoA/alcohol dehydrogenase [*Escherichia coli* O157:H- str. 493-89]	+	+
gi|320643291	Putative protease/scaffold protein [*Escherichia coli* O157:H- str. 493-89]	+	+
gi|320643369	Dihydrolipoamide succinyltransferase [*Escherichia coli* O157:H- str. 493-89]	+	+

gi|320646990	Glyceraldehyde-3-phosphate dehydrogenase A [*Escherichia coli* O157:H- str. 493-89]	+	+
gi|320644596	Glutamine synthetase [*Escherichia coli* O157:H- str. 493-89]	+	+
gi|320646248	Bifunctional malic enzyme oxidoreductase/phospho-transacetylase [*Escherichia coli* O157:H- str. 493-89]	+	+
gi|320642801	DNA starvation/stationary phase protection protein Dps [*Escherichia coli* O157:H- str. 493-89]	+	+
gi|12515069	Unknown protein encoded within prophage CP-933O [*Escherichia coli* O157:H7 str. EDL933]	+	+
gi|320644107	Hypothetical protein ECO9389_13203 [*Escherichia coli* O157:H- str. 493-89]	-	+
gi|85675373	Hypothetical protein [*Escherichia coli* str. K12 substr. W3110]	+	+
gi|320647115	Ferritin [*Escherichia coli* O157:H- str. 493-89]	+	+
gi|320644107	Hypothetical protein ECO9389_13203 [*Escherichia coli* O157:H- str. 493-89]	+	+
**Unknown localization (n = 9)**			
**gi|23574038**	**Cytolethal distending toxin A [*Escherichia coli* O157:H- str. 493/89]**[b]	+	+
gi|320646946	DNA-binding transcriptional activator OsmE [*Escherichia coli* O157:H- str. 493-89]	+	-
gi|320645468	Hypothetical protein ECO9389_07582 [*Escherichia coli* O157:H- str. 493-89]	+	+
gi|446531864	Hypothetical protein [*Escherichia coli*]	+	+
gi|408567512	Hypothetical protein EC80569_2863 [*Escherichia coli* O157:H7 strain 8.0569]	+	-
gi|320646024	Hypothetical protein ECO9389_21050 [*Escherichia coli* O157:H- str. 493-89]	+	+
gi|320644274	Hypothetical protein ECO9389_13978 [*Escherichia coli* O157:H- str. 493-89]	+	+
gi|320646484	Hypothetical protein ECO9389_00362 [*Escherichia coli* O157:H- str. 493-89]	+	+
gi|320643292	Hypothetical protein ECO9389_18210 [*Escherichia coli* O157:H- str. 493-89]	-	+

Known virulence factors of *E. coli* O157:H7/H^- are shown in bold.

[a] +, protein present; -, protein absent.

[b] Cytolethal distending toxin A, B, and C are three components of CdtV holotoxin.
