# Supplementary material for: Host cell interactions of outer membrane vesicle-associated virulence factors of enterohemorrhagic Escherichia coli O157: Intracellular delivery, trafficking and mechanisms of cell injury
Source: PLoS Pathog. 2017 Feb 3;13(2):e1006159. doi: 10.1371/journal.ppat.1006159 (PMC5310930; doi:10.1371/journal.ppat.1006159)
Supplement: S4 Table — (PDF) [file ppat.1006159.s040.pdf]

S4 Table. Concentrations of the total protein and OMV-associated virulence factors in OptiPrep-purified OMVs from *E. coli* O157:H7/H^{-} strains

OMVs from strain	Total protein (µg/ml)	Stx2a (µg/ml)	CdtV (µg/ml)	EHEC-Hly (µg/ml)	H7 flagellin (µg/ml)
5791/99	449±53[a]	51±18	38±11	2±0.8	73±8
493/89	438±21	50±14	37±10	0	0
493/89Δstx_{2a}	422±34	0	36±8	0	0

[a] All values are means ± standard deviations from measurements of five different batches of each OMV preparation.
