# Supplementary material for: Host cell interactions of outer membrane vesicle-associated virulence factors of enterohemorrhagic Escherichia coli O157: Intracellular delivery, trafficking and mechanisms of cell injury
Source: PLoS Pathog. 2017 Feb 3;13(2):e1006159. doi: 10.1371/journal.ppat.1006159 (PMC5310930; doi:10.1371/journal.ppat.1006159)
Supplement: S5 Table — (PDF) [file ppat.1006159.s041.pdf]

S5 Table. PCR primers used for restriction-free cloning of *cdt*V genes and construction of *cdt*V-*B* deletion mutant

Primer designation	Sequence 5´- 3´	*cdt*V amplicon (size)
F-cdtA-p23	TTTGTTTAACTTTAAGAAGGAGATATACATATGG<br>CTAATAAATACACACCTATTTTTATA	*cdt*V-*A*<br>(776 bp)
R-cdtA-p23	TTCCTTTCGGGCTTTGTTAGCAGCCGGATCTCAT<br>TGTTCGCCTCCTGCTGAATGCCTATC	
F-cdtB-p23	TTTGTTTAACTTTAAGAAGGAGATATACATATGA<br>AAAAATATATTATATCTCTGATAGTA	*cdt*V-*B*<br>(809 bp)
R-cdtB-p23	TTCCTTTCGGGCTTTGTTAGCAGCCGGATCTTAT<br>CGTCTGGAAACGCCAACAGGGTAATG	
F-cdtC-p23	TTTGTTTAACTTTAAGAAGGAGATATACATATGA<br>AGAGATTAATAATTATTGTAACTATG	*cdt*V-*C*<br>(545 bp)
R-cdtC-p23	TTCCTTTCGGGCTTTGTTAGCAGCCGGATCTTAA<br>ATAATAGGCGATTCAGTATTTAATGG	
F-cdtA-p23	TTTGTTTAACTTTAAGAAGGAGATATACATATGG<br>CTAATAAATACACACCTATTTTTATA	*cdt*V-*ABC* operon<br>(2142 bp)
R-cdtC-p23	TTCCTTTCGGGCTTTGTTAGCAGCCGGATCTTAA<br>ATAATAGGCGATTCAGTATTTAATGG	
F-del-cdtB	CCGGACGTCAAGAGGCTATCATAATGAAGA	*cdt*V-*ACΔB*<br>(1333 bp)
R-del-cdtB	CCGGACGTCCTATCTGGTTCTATTGGTAGTG	

Underlined are sequences targeting the insertion region of pET23b(+) vector; non-underlined sequences target the indicated *cdt*V genes.
