# Supplementary material for: Host cell interactions of outer membrane vesicle-associated virulence factors of enterohemorrhagic Escherichia coli O157: Intracellular delivery, trafficking and mechanisms of cell injury
Source: PLoS Pathog. 2017 Feb 3;13(2):e1006159. doi: 10.1371/journal.ppat.1006159 (PMC5310930; doi:10.1371/journal.ppat.1006159)
Supplement: S1 Text — (PDF) [file ppat.1006159.s042.pdf]

S1 Text. Expression and purification of free CdtV-B

To construct the CdtV-B expressing clone M15[pREP4](pQE60/*cdtV-B*$_{493/89}$), a 775 bp fragment containing *cdt*V-*B* gene was amplified from strain 493/89 using primers cdtBpQE60-F (ACCGCCATGGCGGATTTAACTG) and cdtBpQE60-R (ATTCGGATCC TCGTCTGGAAACGC), with overhangs homologous to *Nco*I and *BamH*I restriction site, respectively (underlined). The amplicon was purified with QIAquick PCR purification kit (Qiagen) and ligated into *Nco*I/*BamH*I (New England Biolabs)-digested pQE60 vector (Qiagen) using T4 DNA Ligase (Fermentas). The construct was transformed into *E. coli* M15[pREP4] host using the QIAexpressionist kit (Qiagen) according to the manufacturer´s instructions. Clones were selected on LB agar with kanamycin (25 µg/ml) and ampicillin (100 µg/ml) after overnight incubation and the correct orientation of the insert was verified by sequencing. To express CdtV-B, the strain was grown in Super Broth (SB) (Difco) with 100µg/ml of ampicillin and 25µg/ml of kanamycin (37°C, 250 rpm) until OD_{600} ~0.6. The culture was induced with 0.4 mM isopropyl β-D-1-thiogalactopyranoside (Sigma) and incubated for 14 h (37°C, 250 rpm). After centrifugation (5000 x g, 15 min, 4°C), the pellet was used to purify CdtV-B using Protino-Ni-TED 1000 packed columns kit (Macherey-Nagel) under native conditions as recommended by the manufacturer. Briefly, 1 g of the pellet was resuspended in LEW buffer and treated with lysozyme (Sigma) (1 mg/ml) for 30 min on ice. 1 mM phenylmethylsulfonyl fluoride (Sigma) was added and the suspension was sonicated (Sonopuls; Bandelin Electronic) with ten 15 s bursts, on ice. The lysate was centrifuged (10000 x g, 30 min, 4°C) and the supernatant was loaded on Ni-TED 1000 column which had been equilibrated with LEW buffer. CdtV-B was eluted from the column with 125 mM imidazole elution buffer. The purity of the protein was verified using SDS-PAGE and Coomassie blue staining. Protein concentration of the purified CdtV-B was determined using Nanoquant reagent (Carl Roth).
